# Supplementary figures and images for: Caenorhabditis elegans Dicer acts with the RIG-I-like helicase DRH-1 and RDE-4 to cleave dsRNA
Source: eLife. 2024 May 15;13:RP93979. doi: 10.7554/eLife.93979 (PMC11095941; doi:10.7554/eLife.93979)

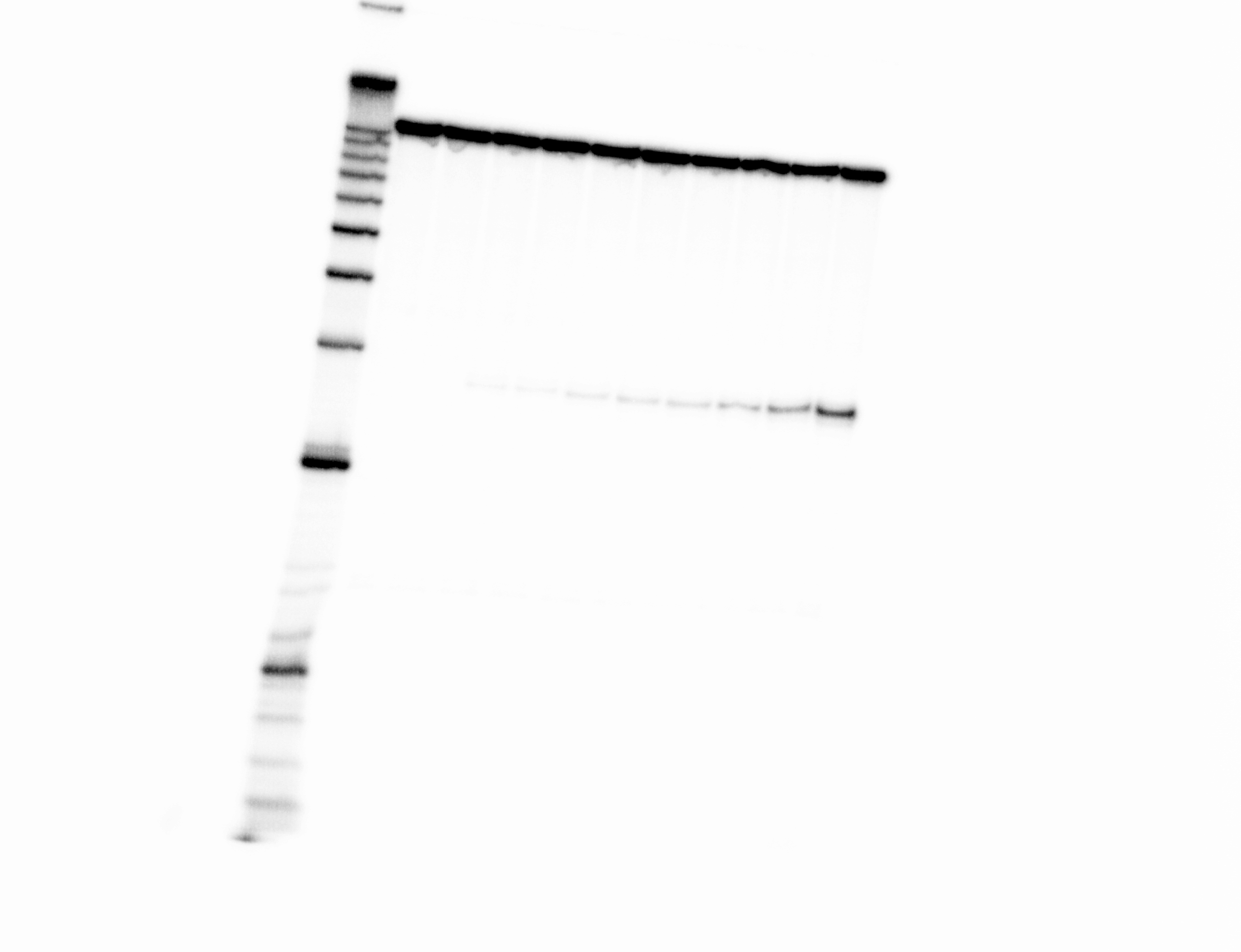

Supplement: Figure 1—source data 1. [file elife-93979-fig1-data1.zip › FIGURE 1 - SOURCE DATA 1.bmp]

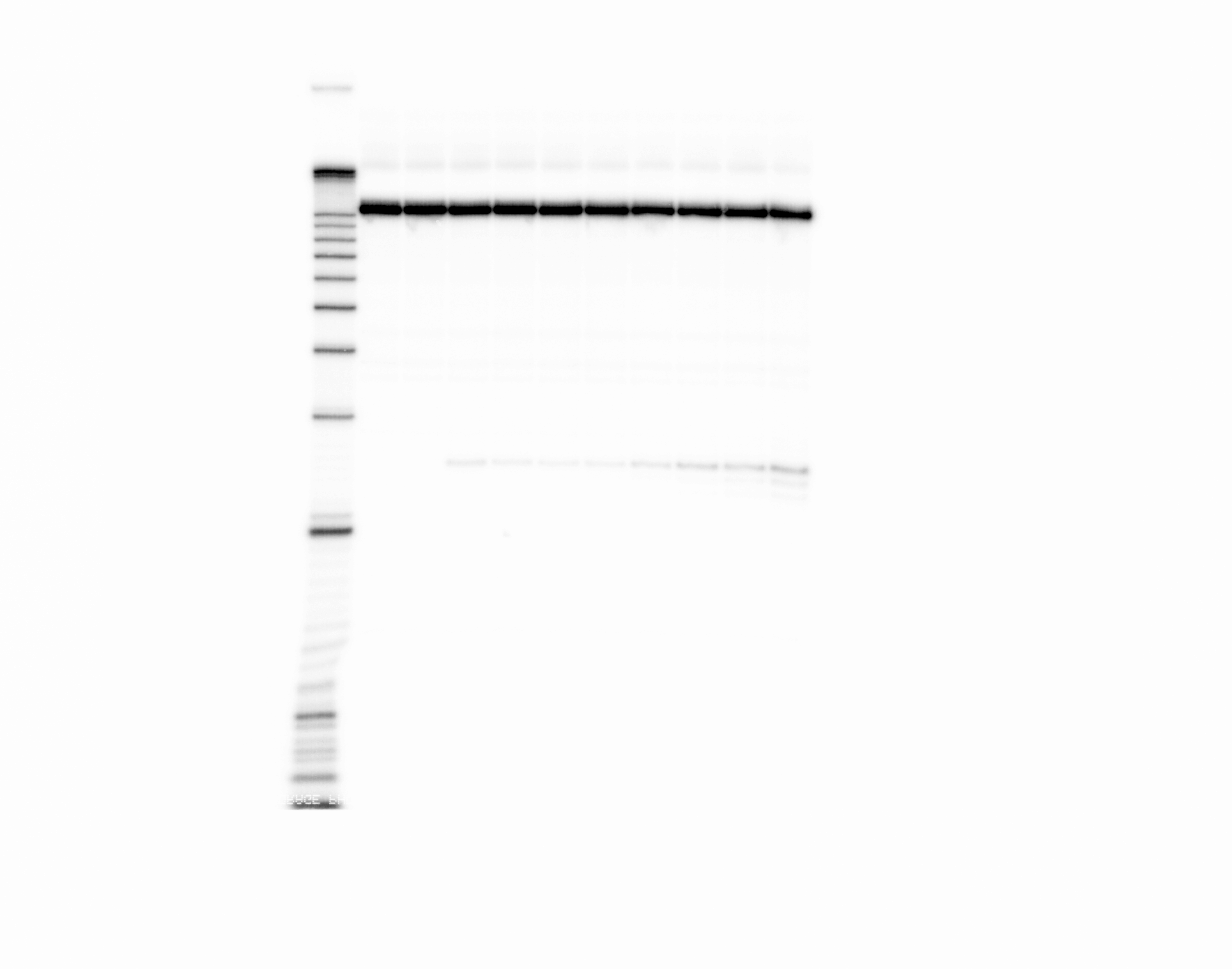

Supplement: Figure 1—source data 2. [file elife-93979-fig1-data2.zip › FIGURE 1 - SOURCE DATA 2.bmp]

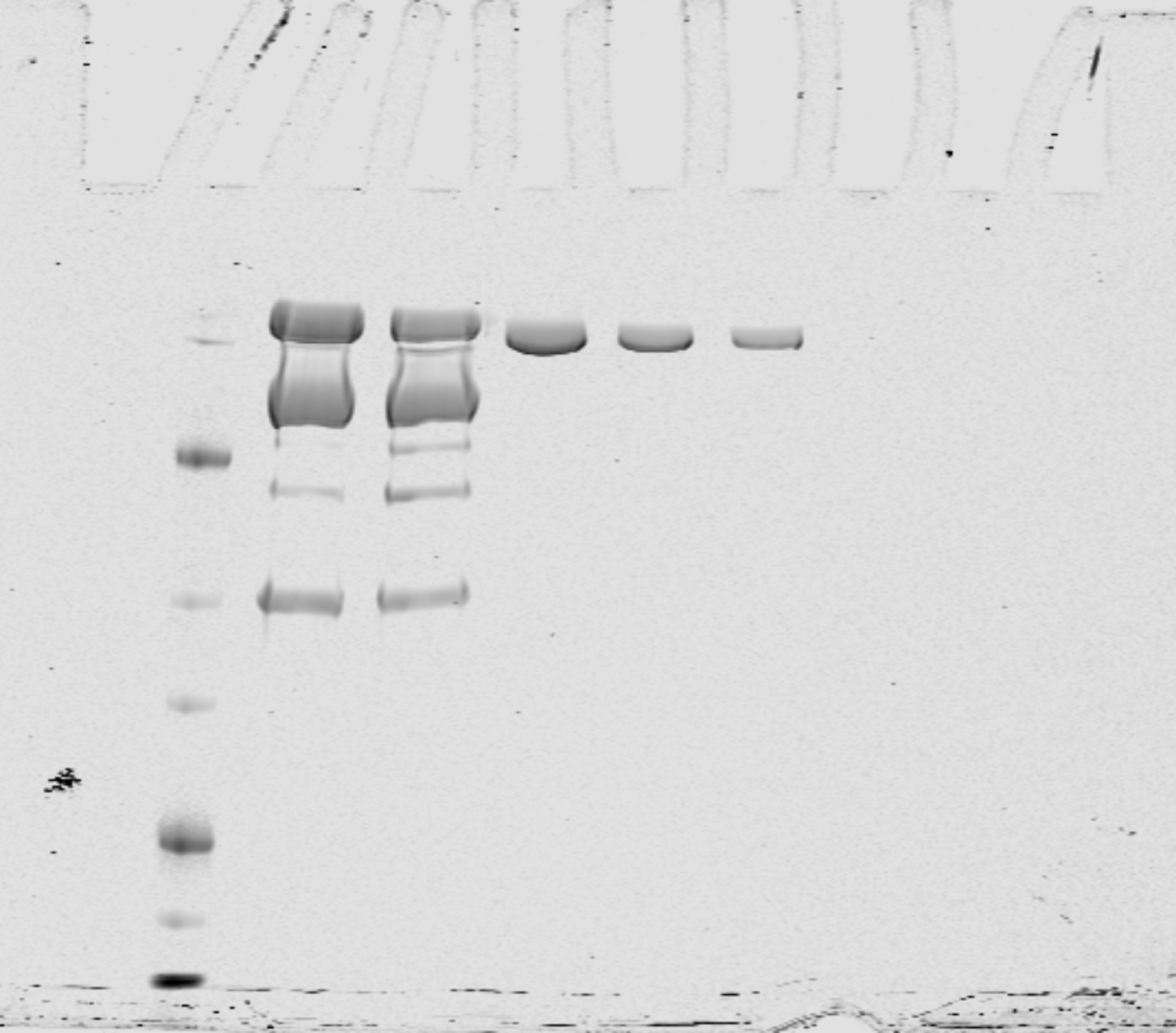

Supplement: Figure 1—figure supplement 1—source data 1. [file elife-93979-fig1-figsupp1-data1.zip › Figure 1 - figure supplement 1 - source data 1.tif]

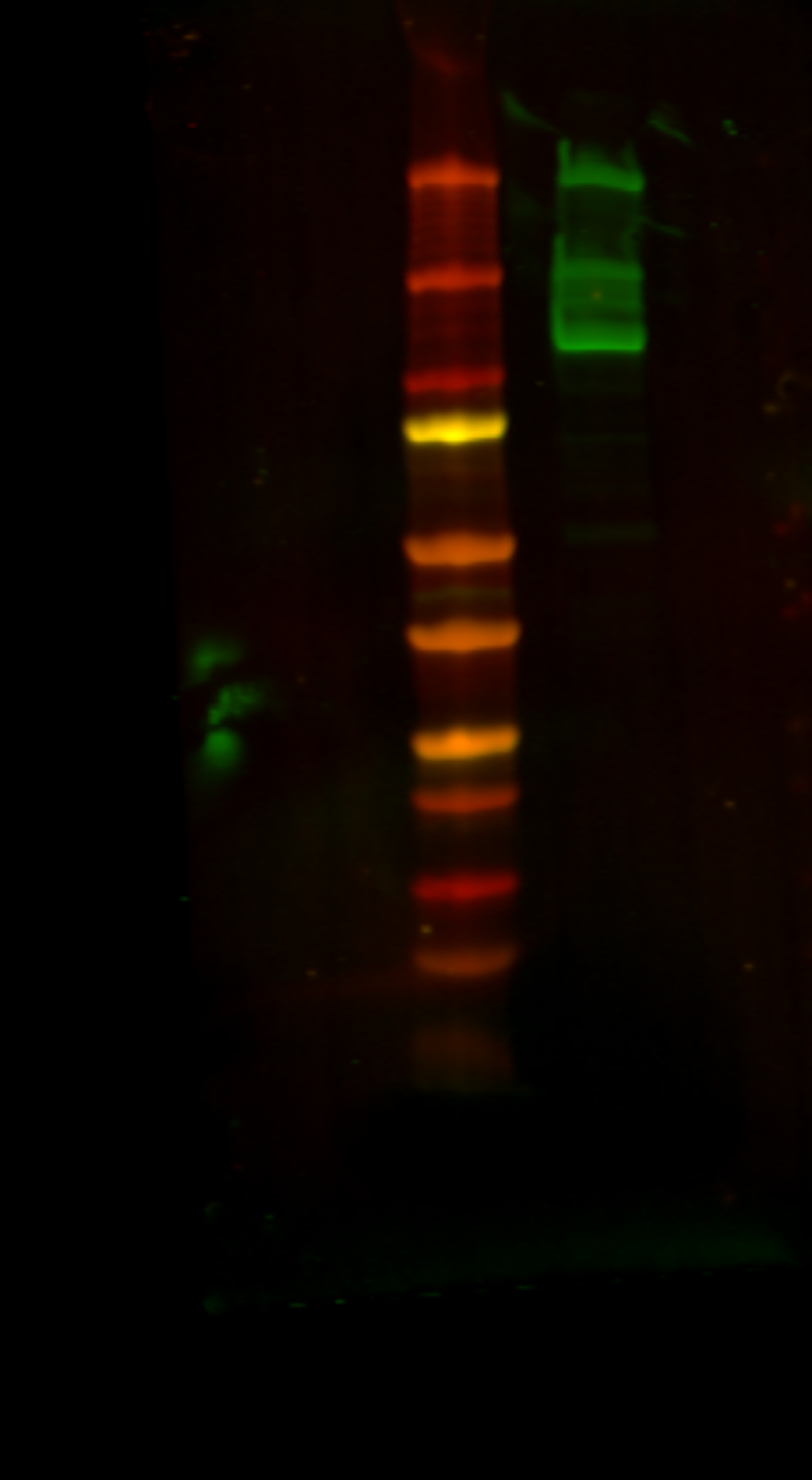

Supplement: Figure 1—figure supplement 1—source data 2. [file elife-93979-fig1-figsupp1-data2.zip › Figure 1 - figure supplement 1 - source data 2.tif]

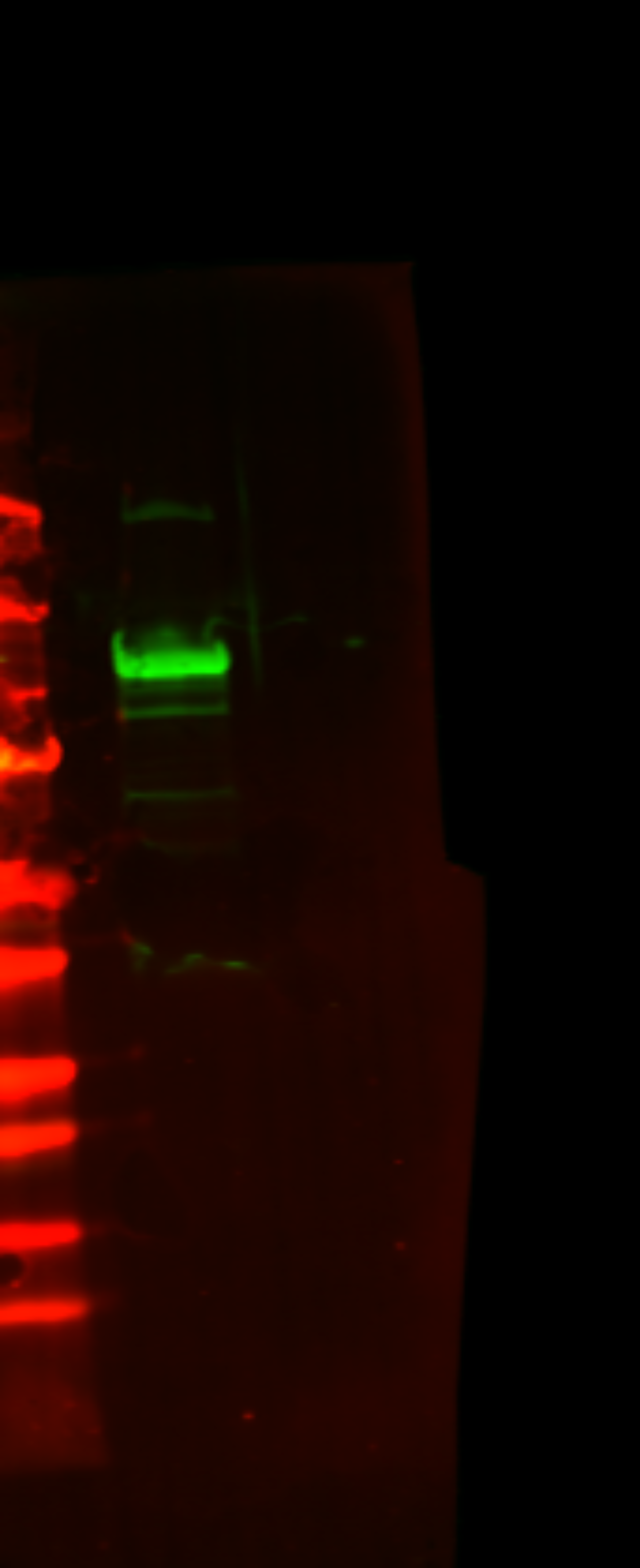

Supplement: Figure 1—figure supplement 1—source data 3. [file elife-93979-fig1-figsupp1-data3.zip › Figure 1 - figure supplement 1 - source data 3.tif]

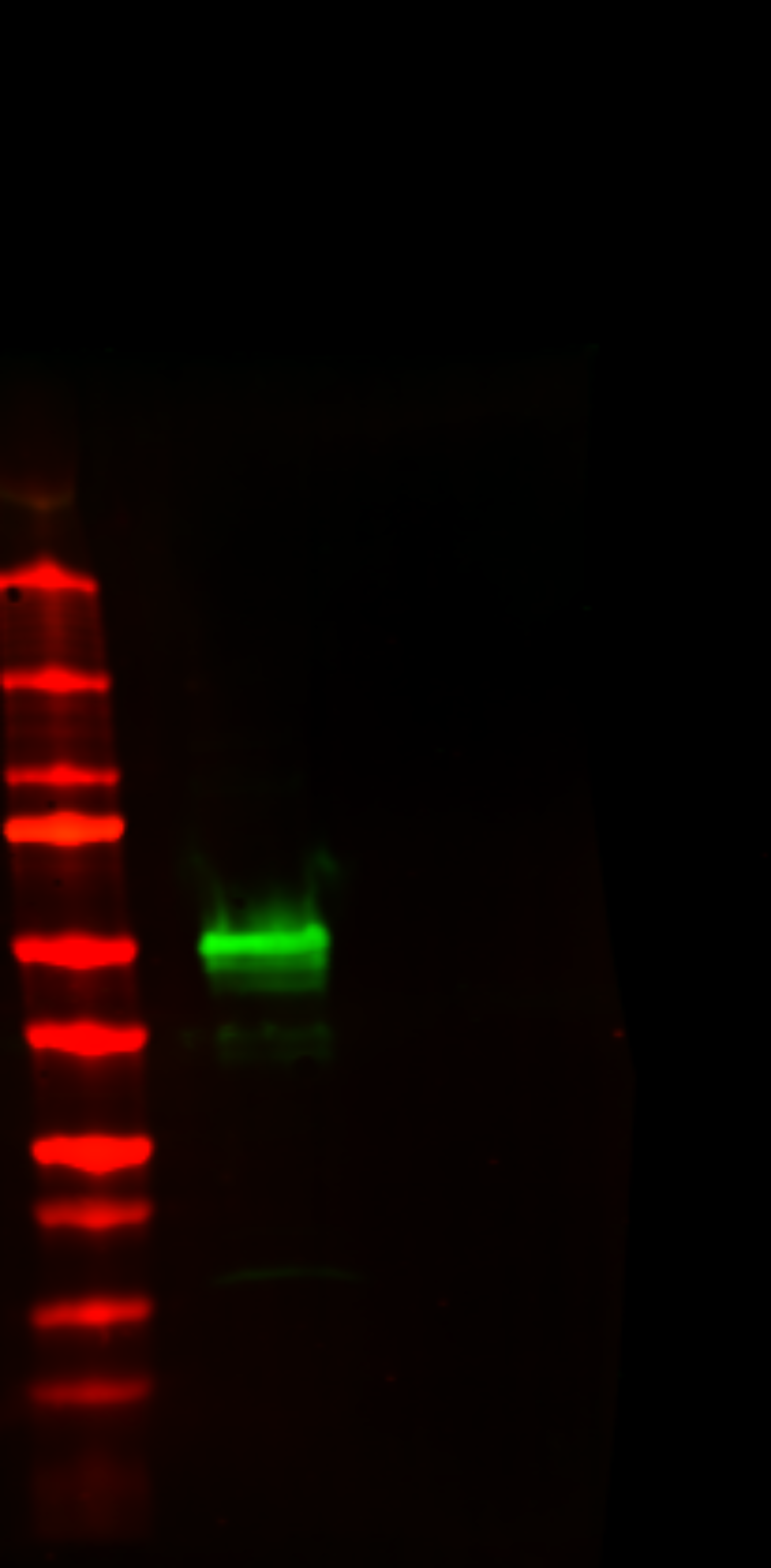

Supplement: Figure 1—figure supplement 1—source data 4. [file elife-93979-fig1-figsupp1-data4.zip › Figure 1 - figure supplement 1 - source data 4.tif]

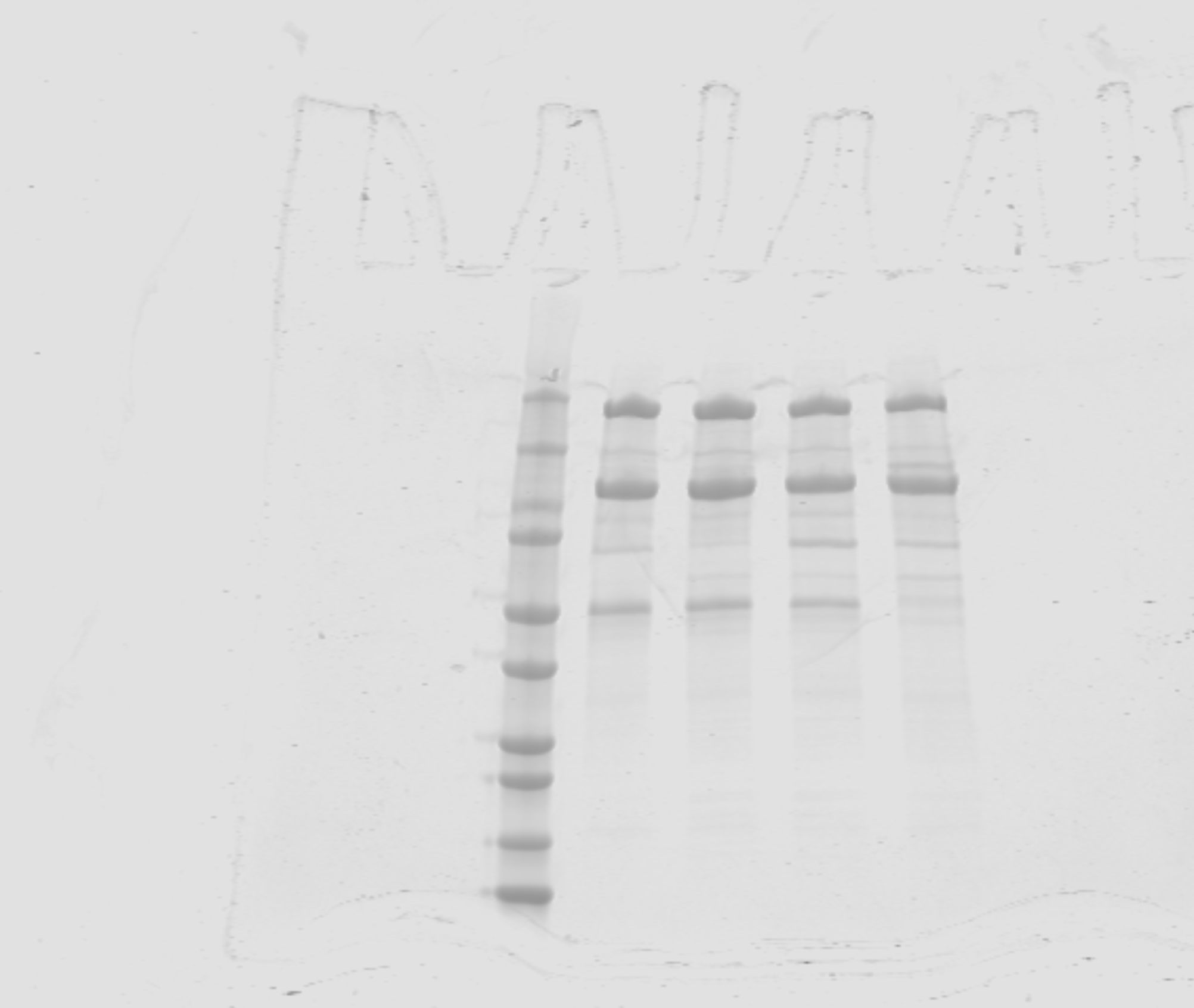

Supplement: Figure 1—figure supplement 1—source data 5. [file elife-93979-fig1-figsupp1-data5.zip › Figure 1 - figure supplement 1 - source data 5.tif]

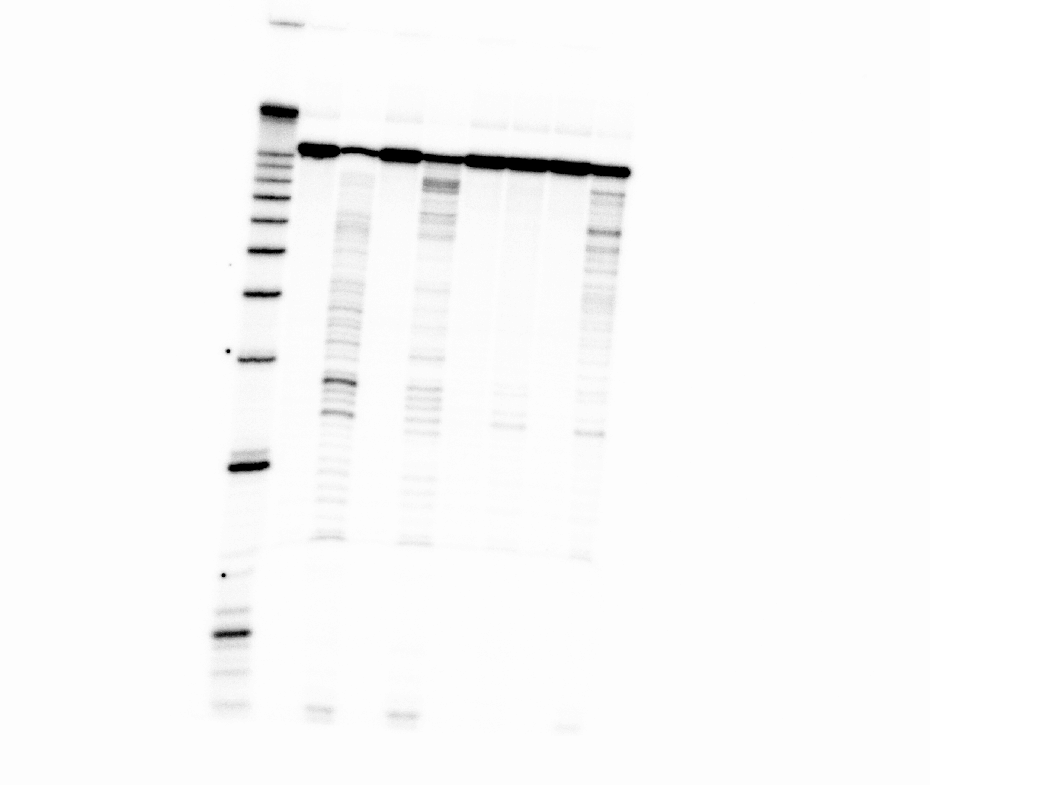

Supplement: Figure 1—figure supplement 2—source data 1. [file elife-93979-fig1-figsupp2-data1.zip › Figure 1 - figure supplement 2 - source data 1.bmp]

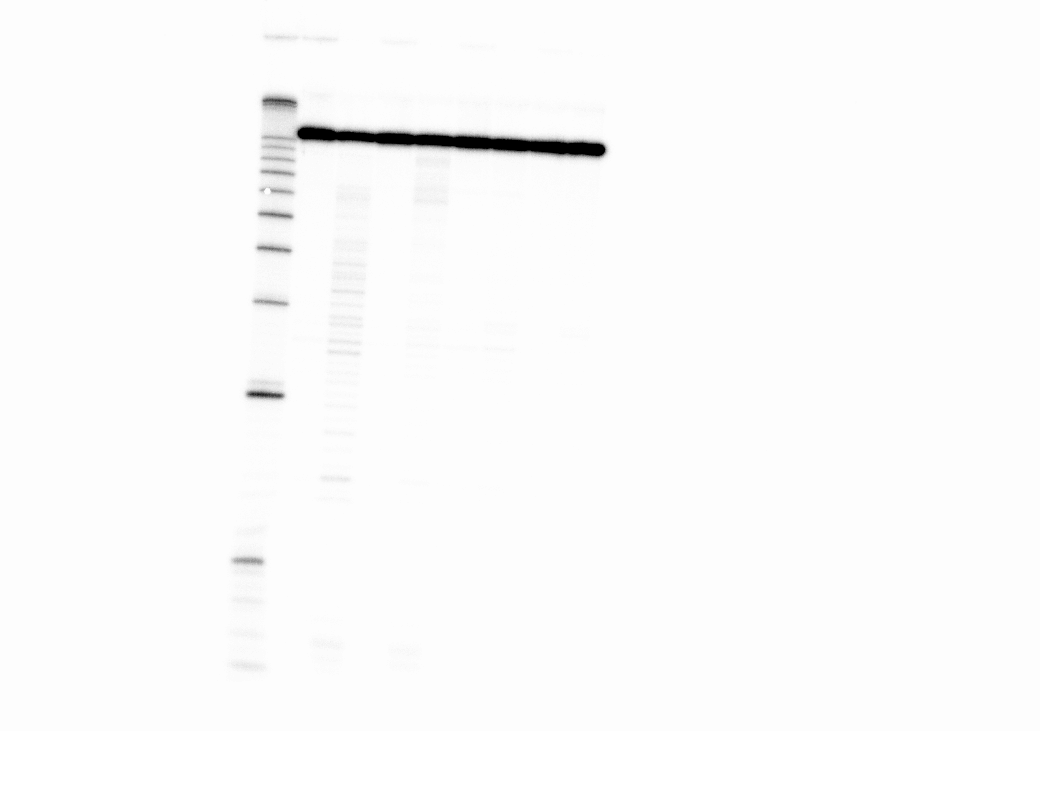

Supplement: Figure 1—figure supplement 2—source data 2. [file elife-93979-fig1-figsupp2-data2.zip › Figure 1 - figure supplement 2 - source data 2.bmp]

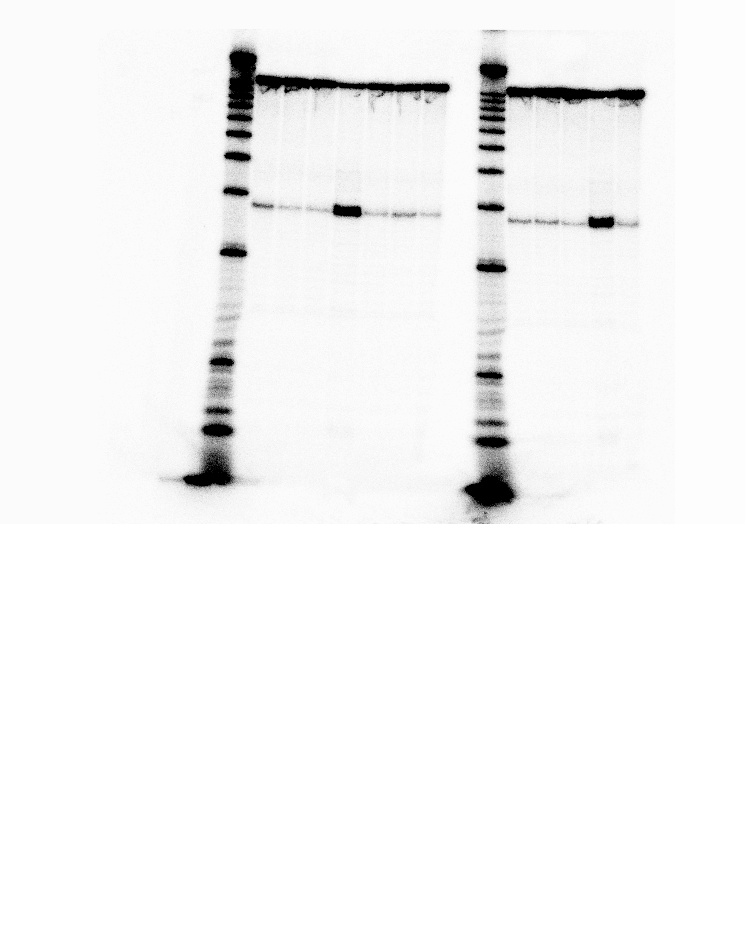

Supplement: Figure 1—figure supplement 3—source data 1. [file elife-93979-fig1-figsupp3-data1.zip › Figure 1 - figure supplement 3 - source data 1.bmp]

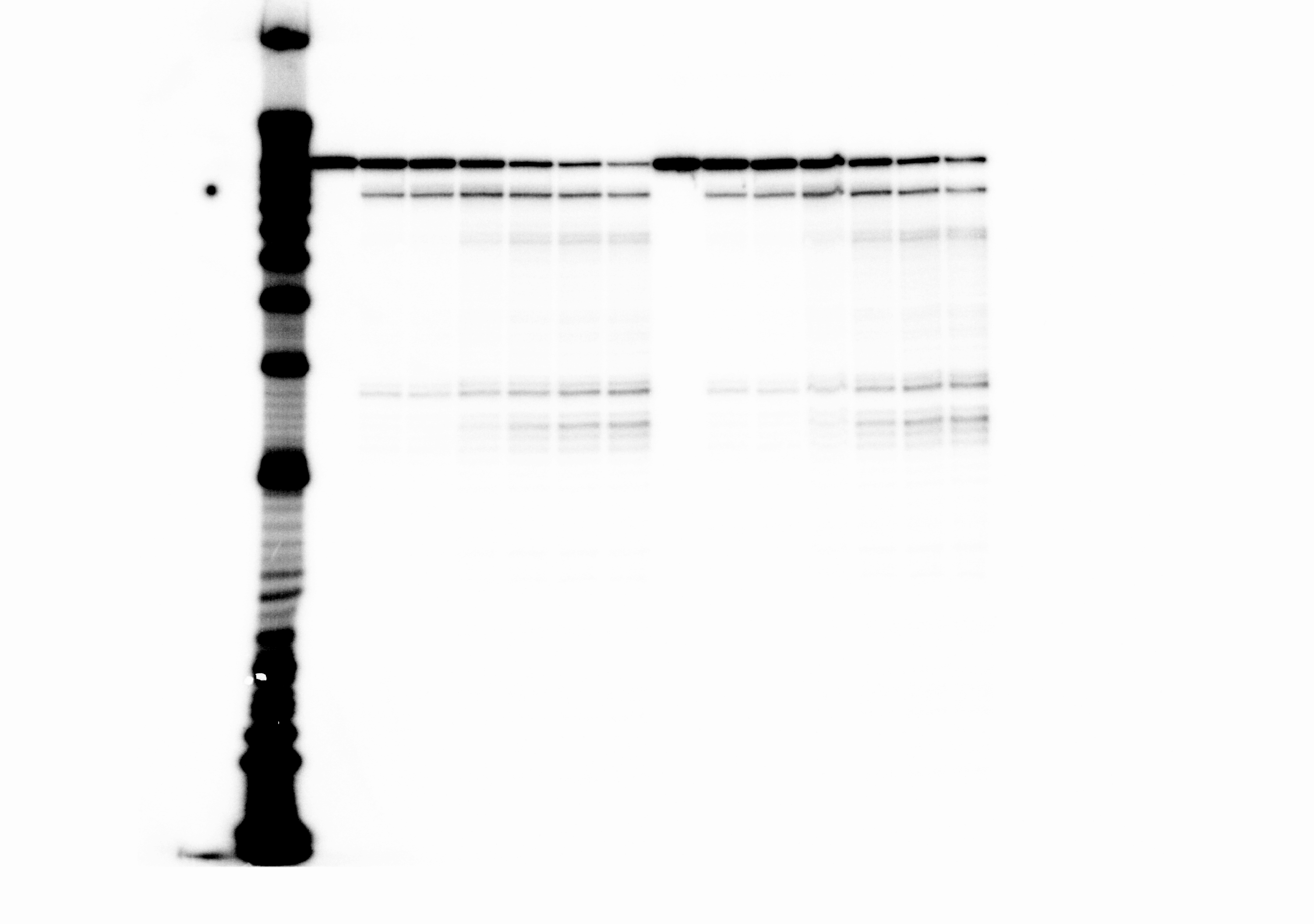

Supplement: Figure 1—figure supplement 3—source data 2. [file elife-93979-fig1-figsupp3-data2.zip › Figure 1 - figure supplement 3 - source data 2.bmp]

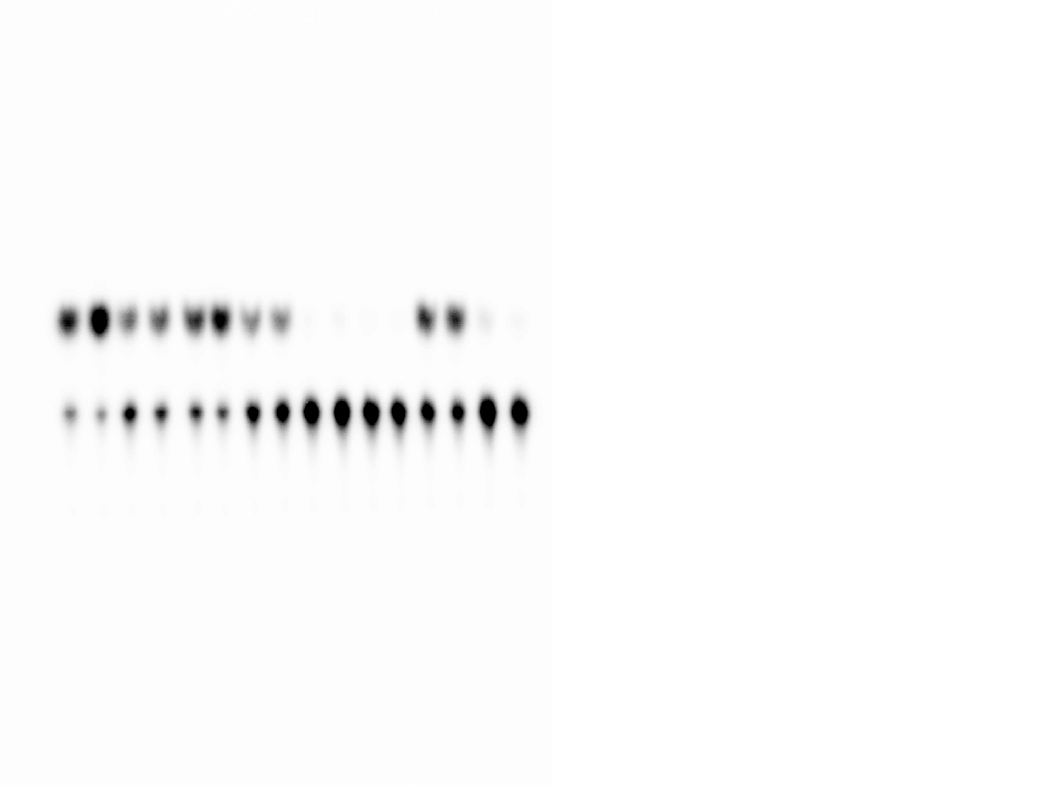

Supplement: Figure 1—figure supplement 3—source data 3. [file elife-93979-fig1-figsupp3-data3.zip › Figure 1 - figure supplement 3 - source data 3.bmp]

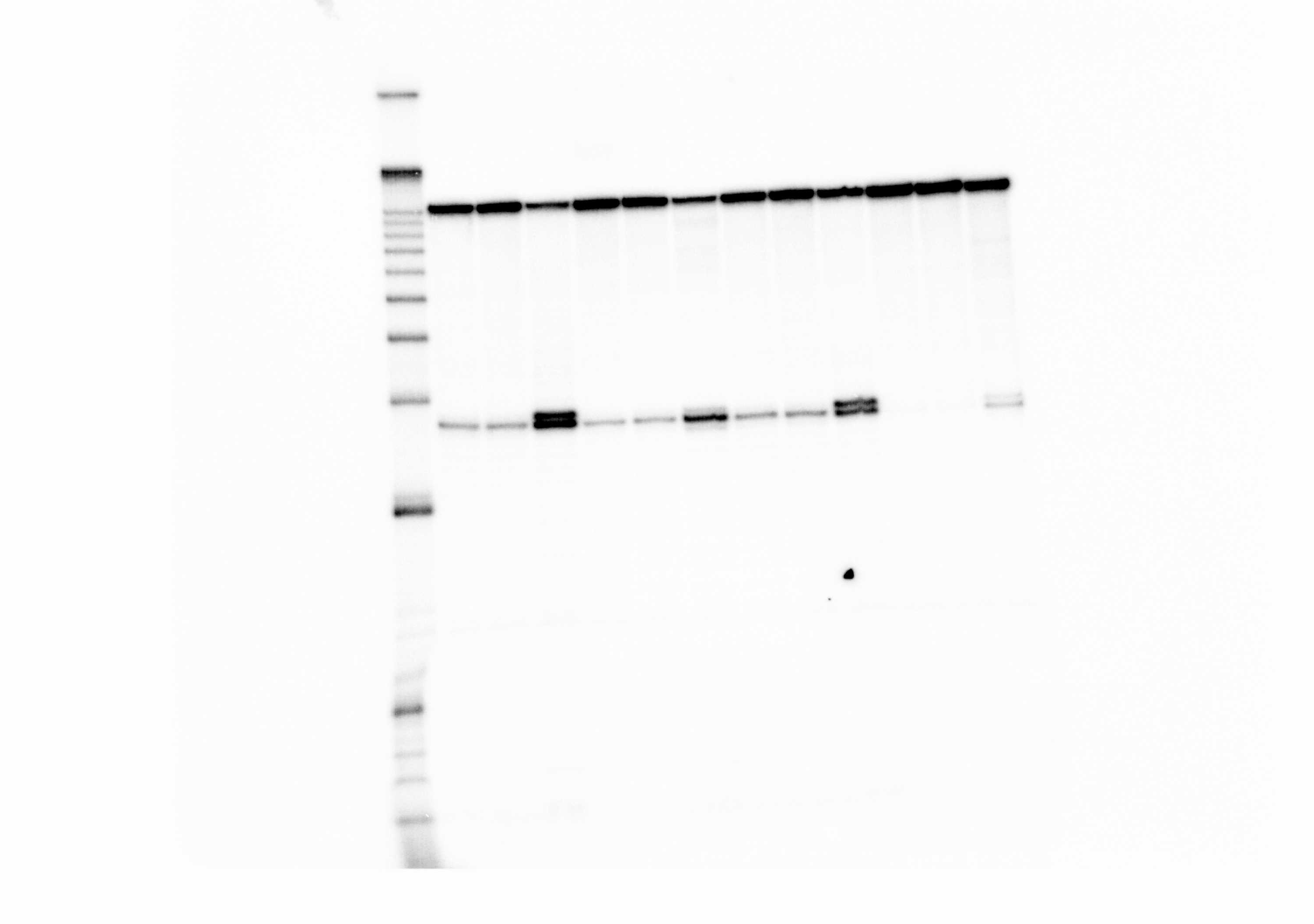

Supplement: Figure 2—source data 1. [file elife-93979-fig2-data1.zip › FIGURE 2 - SOURCE DATA 1.bmp]

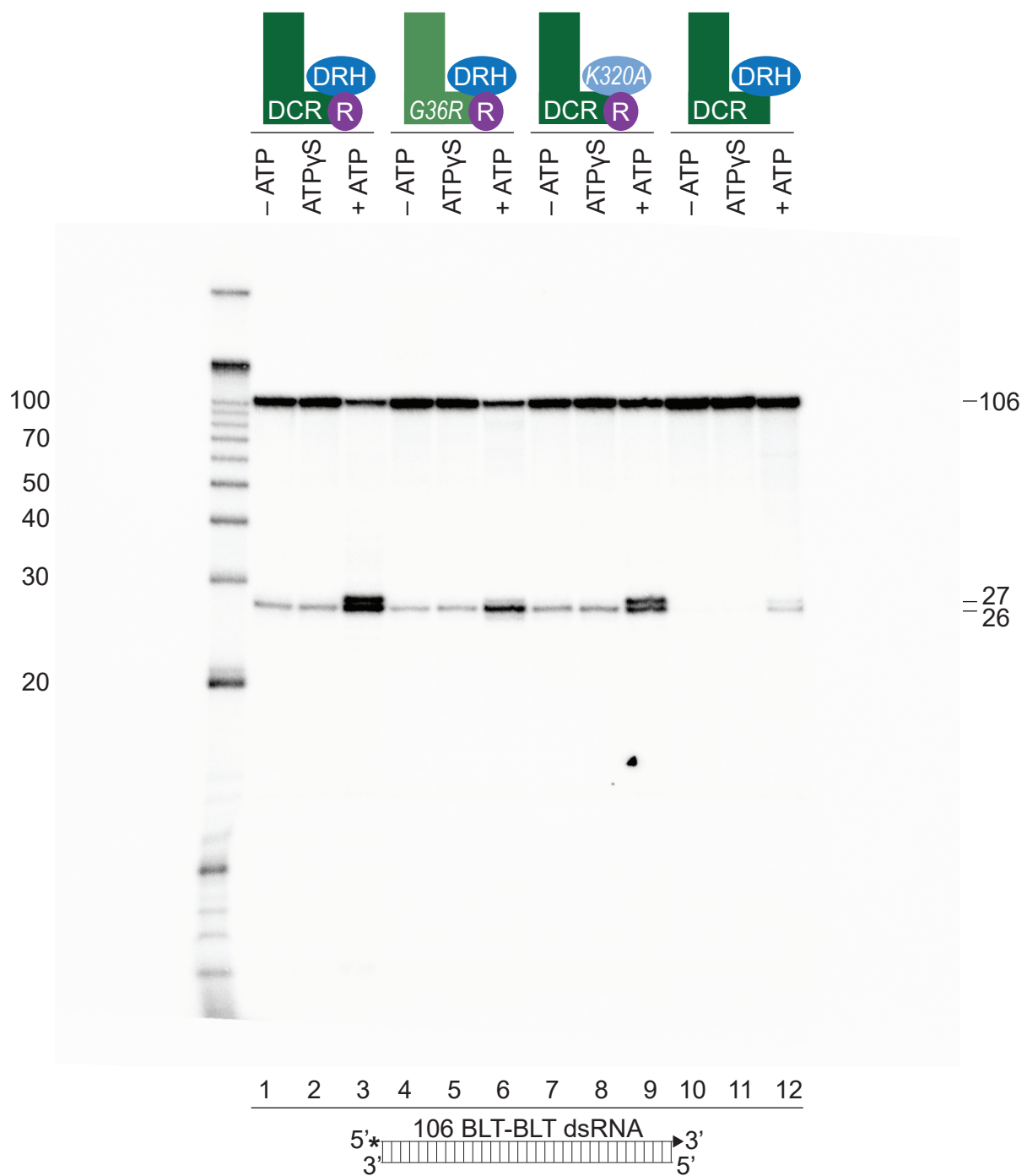

Figure 2 - source data 1: Raw digital image of cleavage phosphorimager plate used in Figure 2A.

Supplement: Figure 2—source data 1. [file elife-93979-fig2-data1.zip › FIGURE 2 - SOURCE DATA 1.pdf]

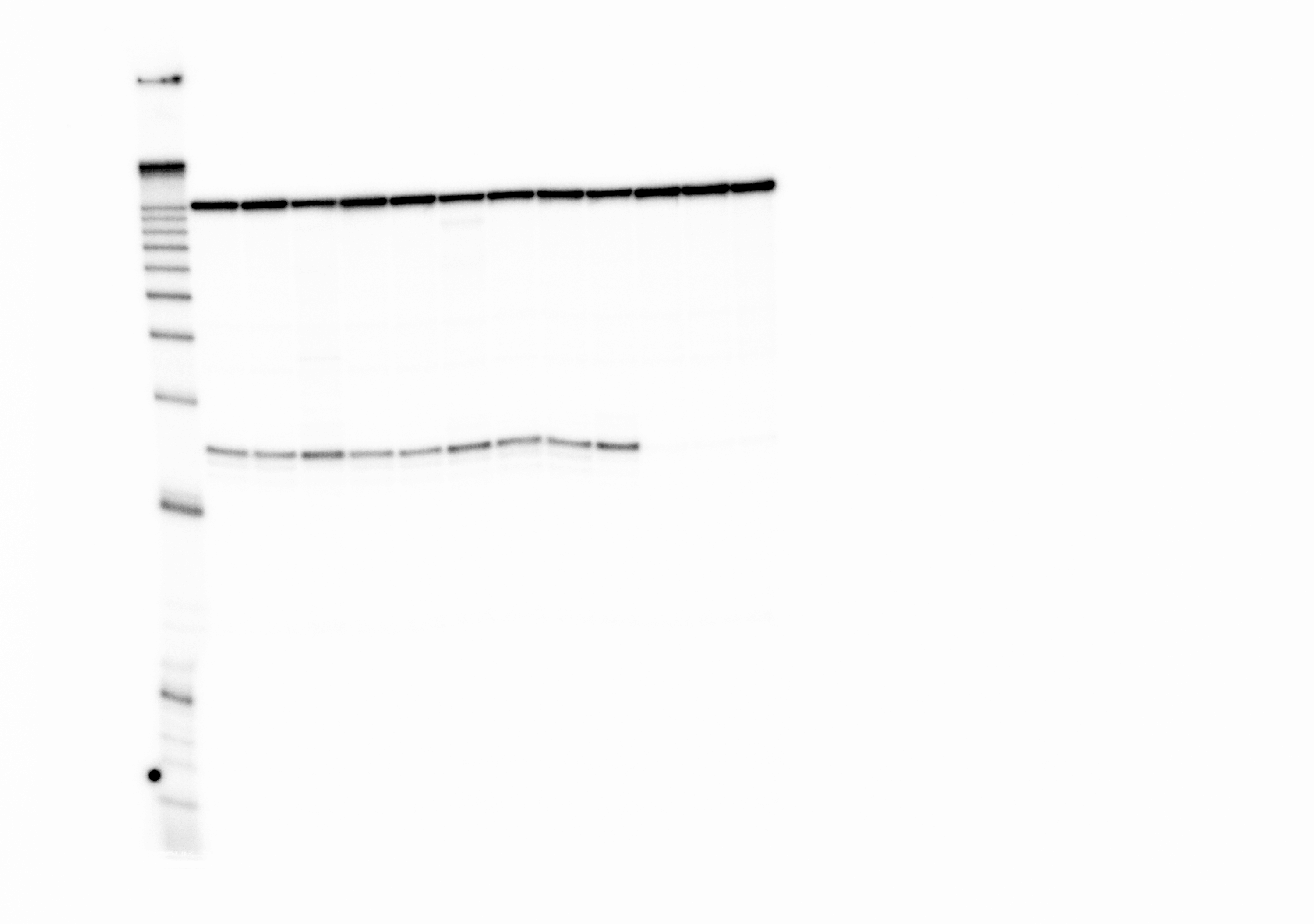

Supplement: Figure 2—source data 2. [file elife-93979-fig2-data2.zip › FIGURE 2 - SOURCE DATA 2.bmp]

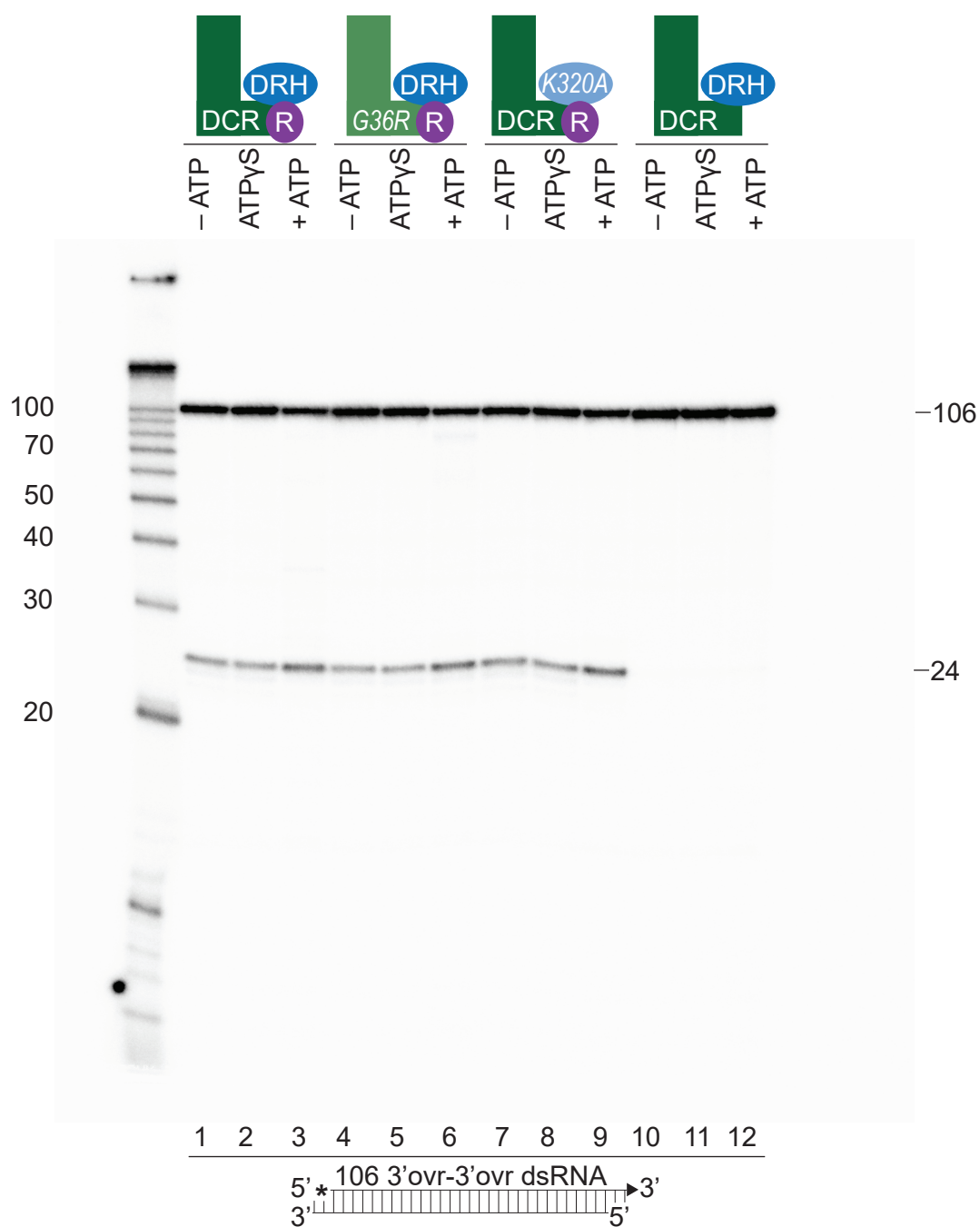

Figure 2 - source data 2: Raw digital image of cleavage phosphorimager plate used in Figure 2B.

Supplement: Figure 2—source data 2. [file elife-93979-fig2-data2.zip › FIGURE 2 - SOURCE DATA 2.pdf]

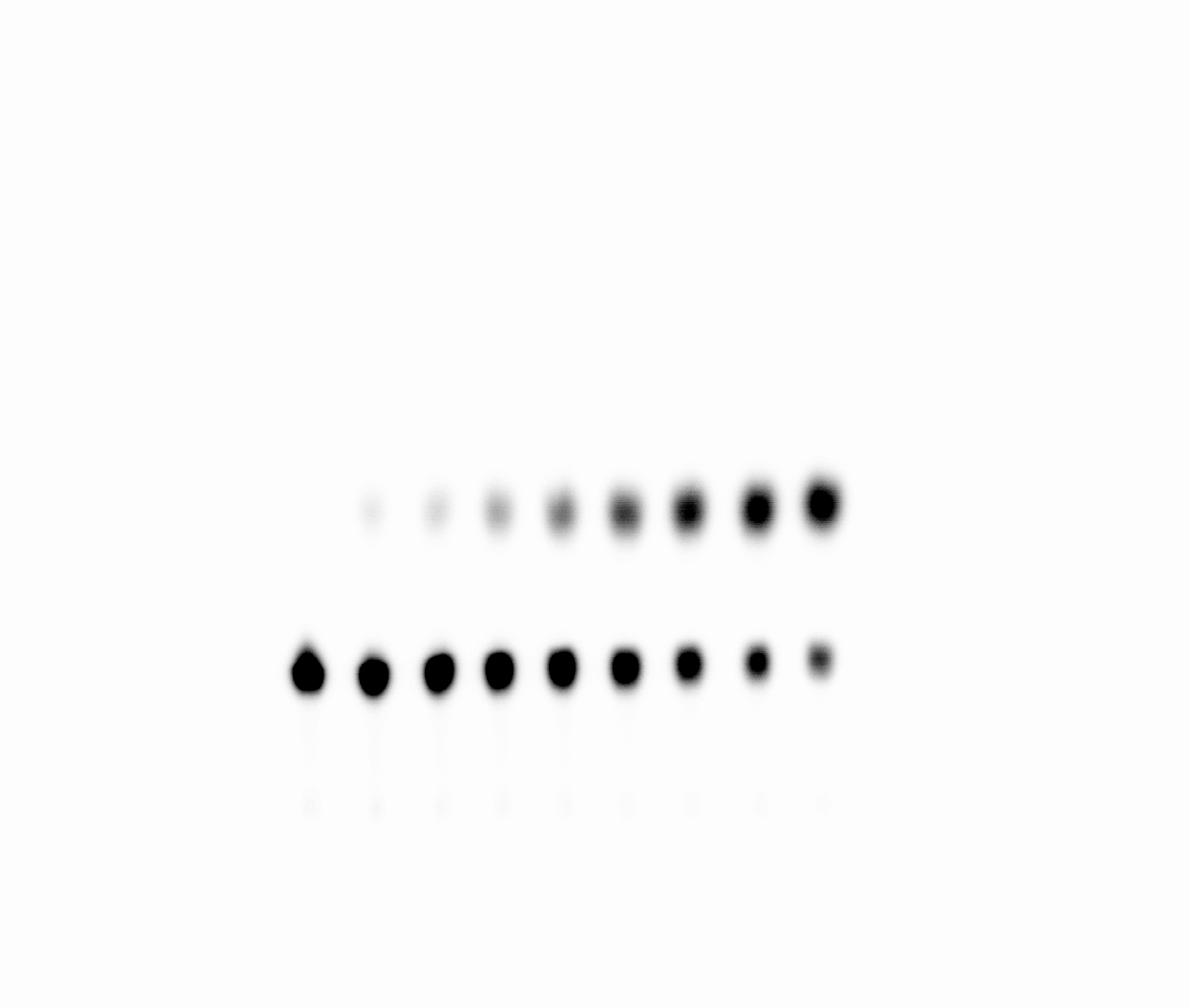

Supplement: Figure 2—source data 4. [file elife-93979-fig2-data4.zip › FIGURE 2 - SOURCE DATA 4.bmp]

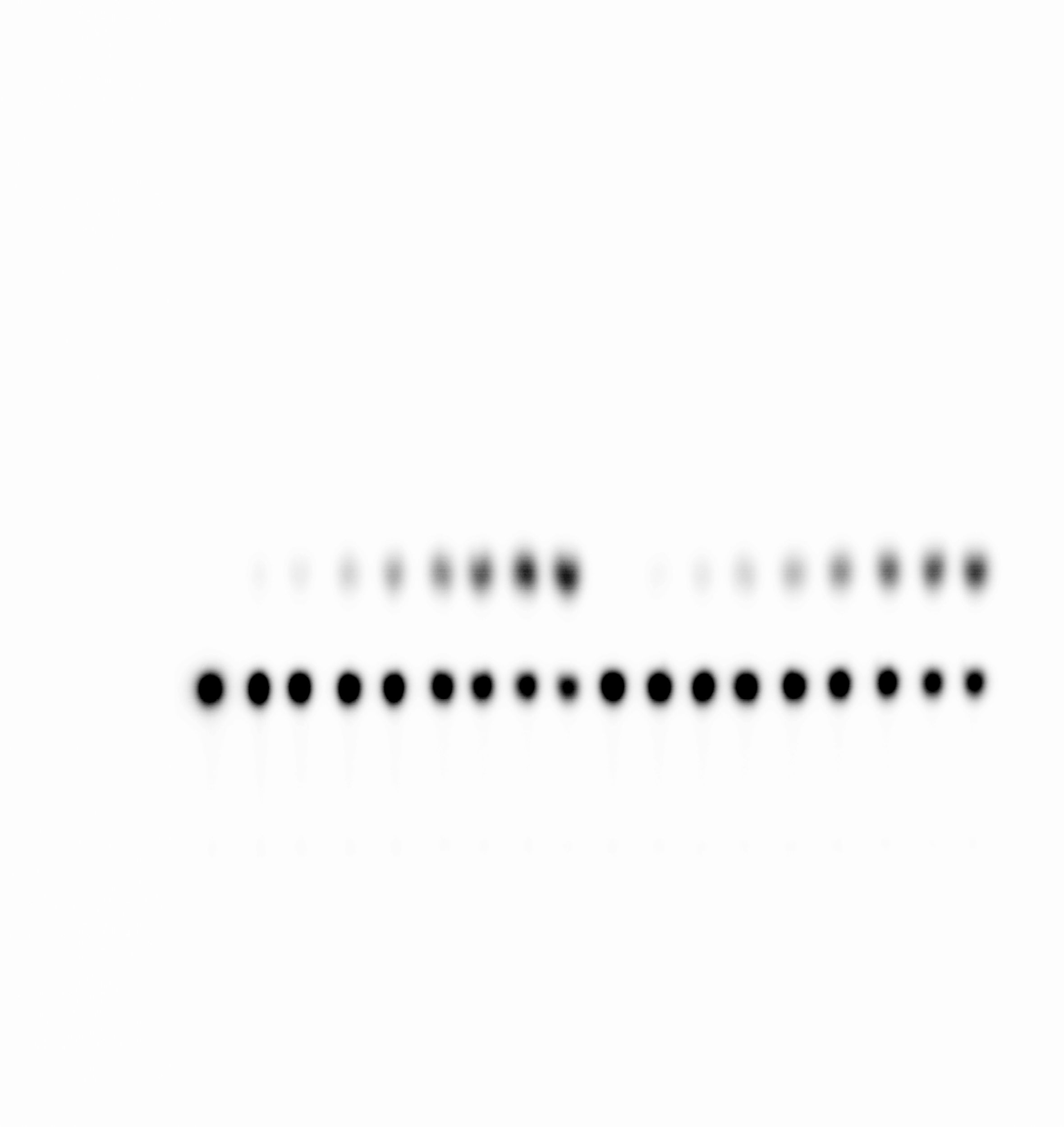

Supplement: Figure 2—source data 5. [file elife-93979-fig2-data5.zip › FIGURE 2 - SOURCE DATA 5.bmp]

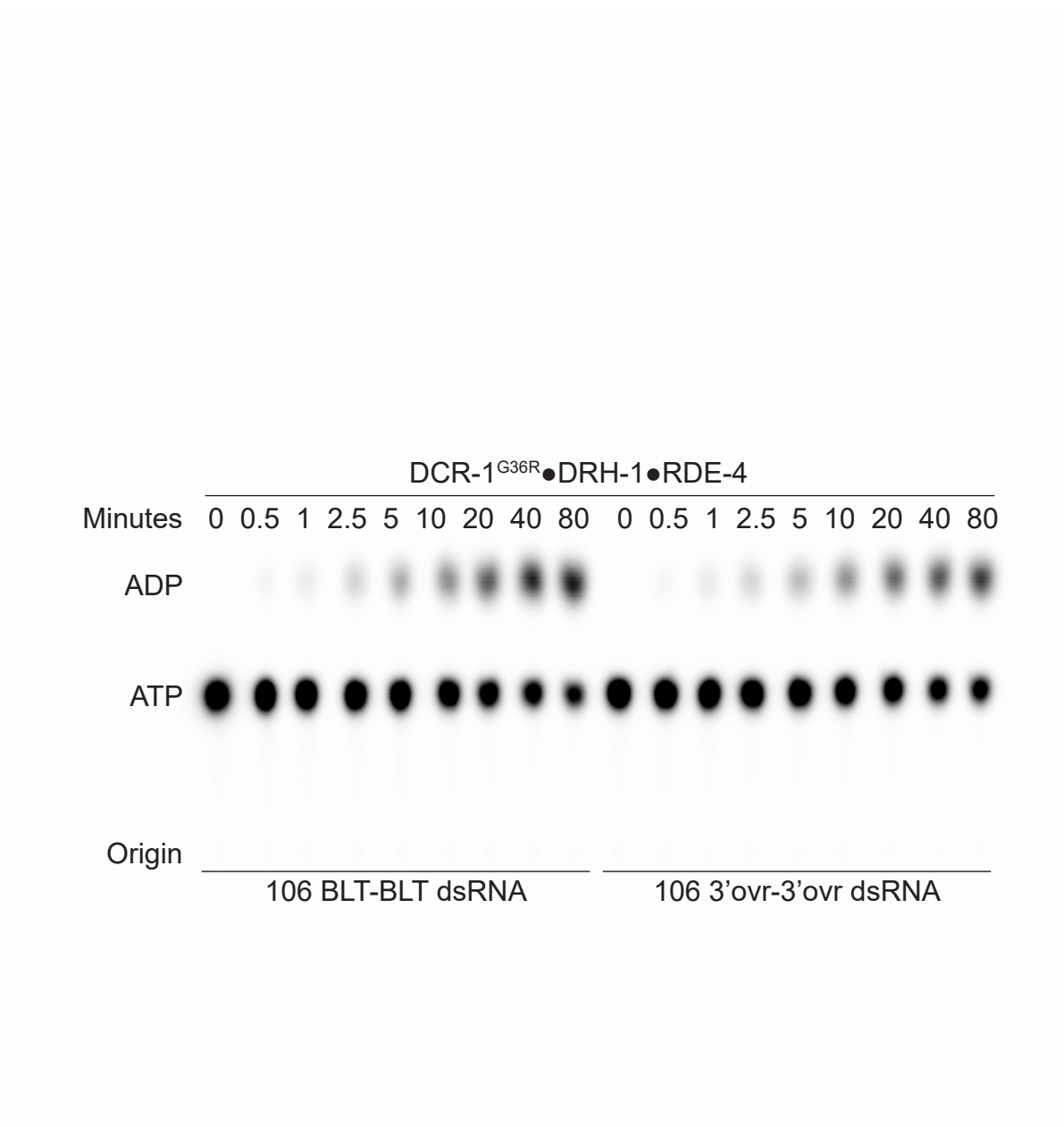

Figure 2 - source data 5: Raw digital image of thin-layer chromatography plate used in Figure 2E.

Supplement: Figure 2—source data 5. [file elife-93979-fig2-data5.zip › FIGURE 2 - SOURCE DATA 5.pdf]

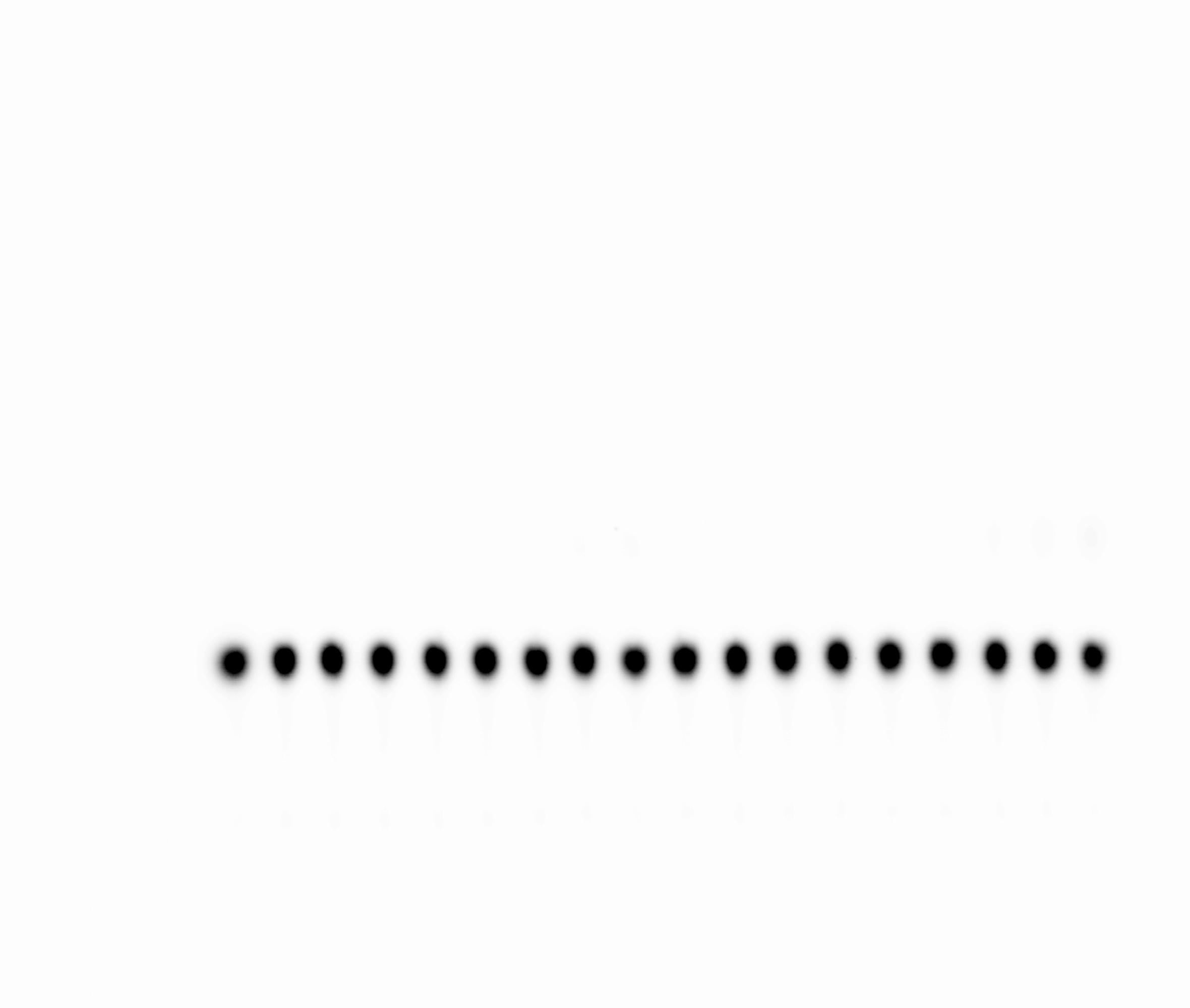

Supplement: Figure 2—source data 6. [file elife-93979-fig2-data6.zip › FIGURE 2 - SOURCE DATA 6.bmp]

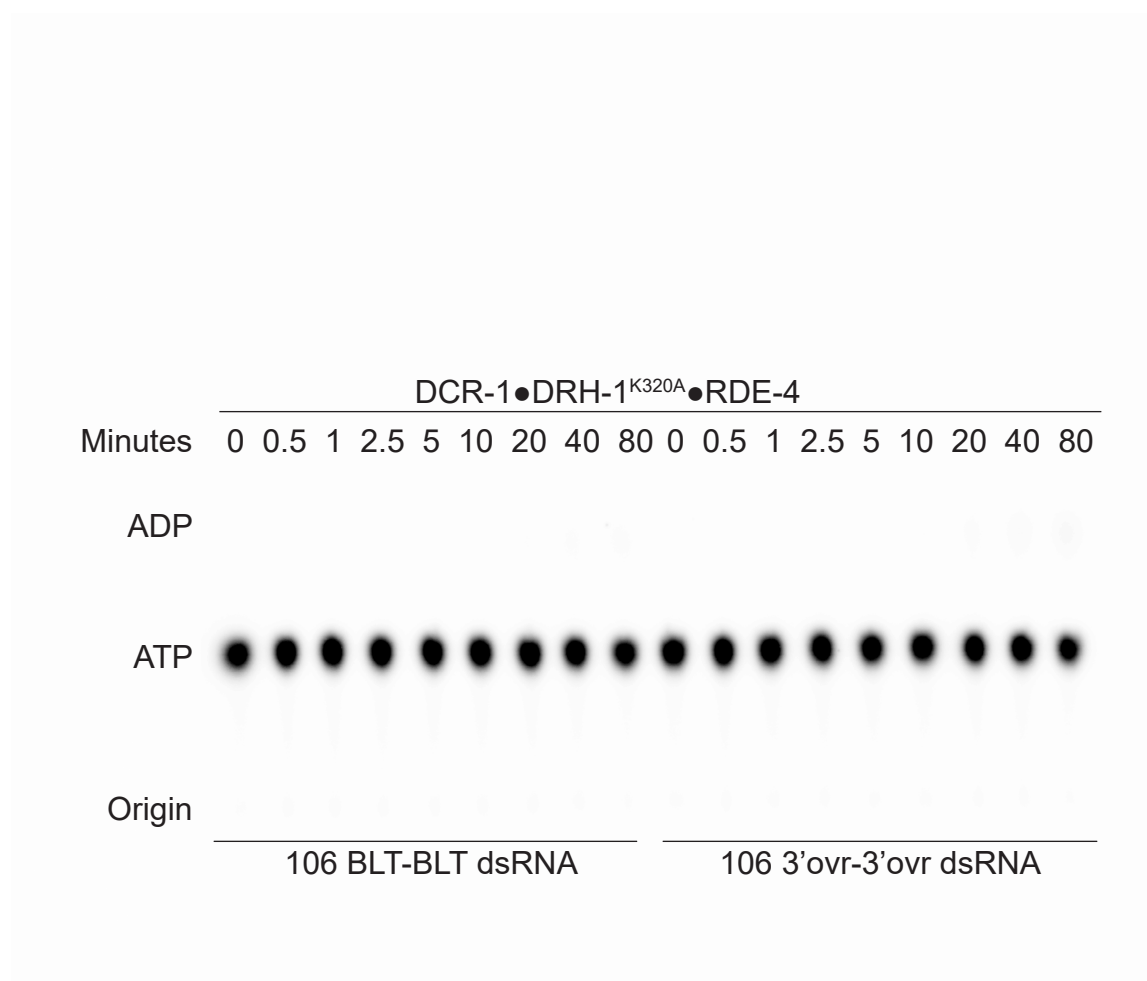

Figure 2 - source data 6: Raw digital image of thin-layer chromatography plate used in Figure 2F.

Supplement: Figure 2—source data 6. [file elife-93979-fig2-data6.zip › FIGURE 2 - SOURCE DATA 6.pdf]

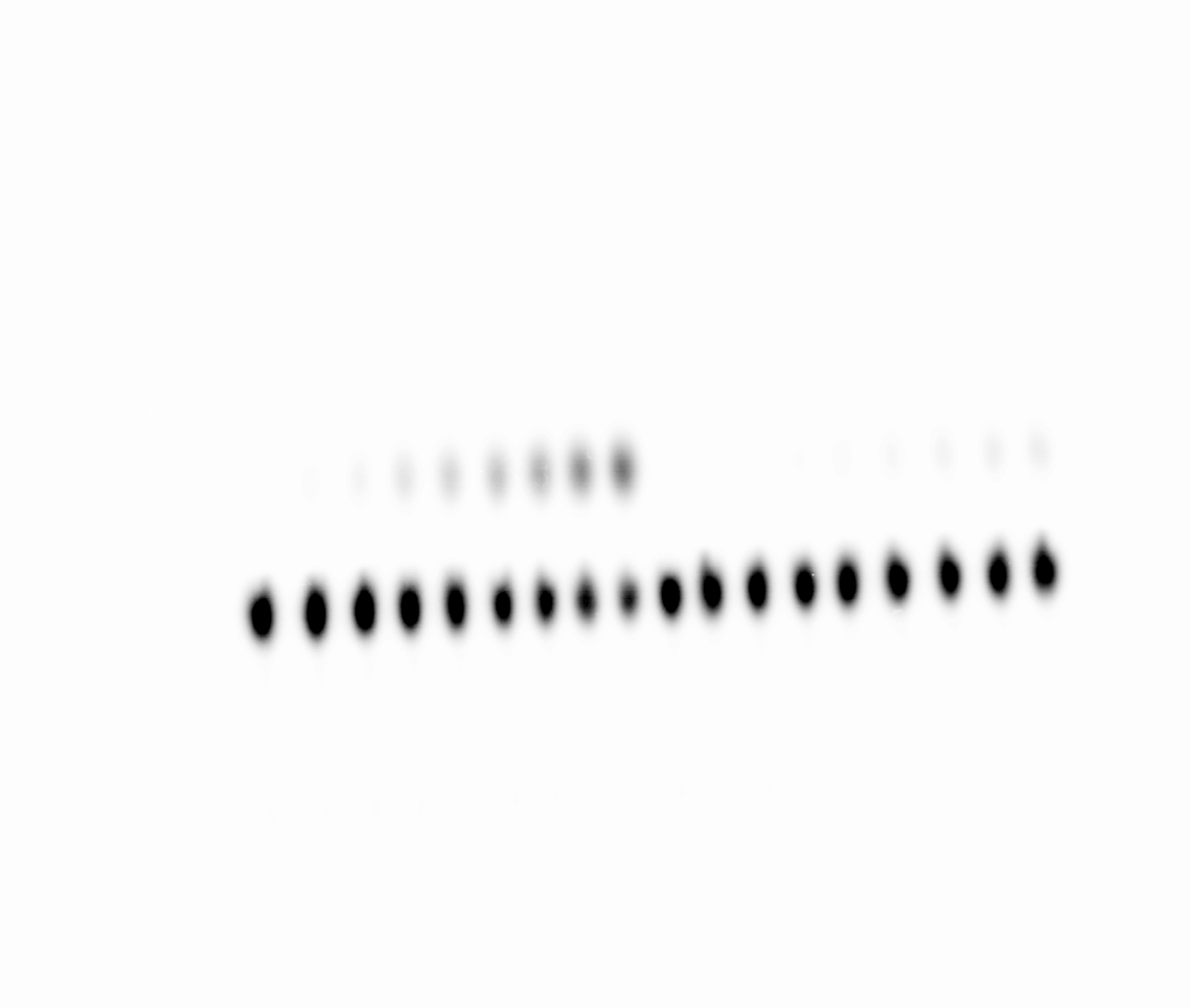

Supplement: Figure 2—source data 7. [file elife-93979-fig2-data7.zip › FIGURE 2 - SOURCE DATA 7.bmp]

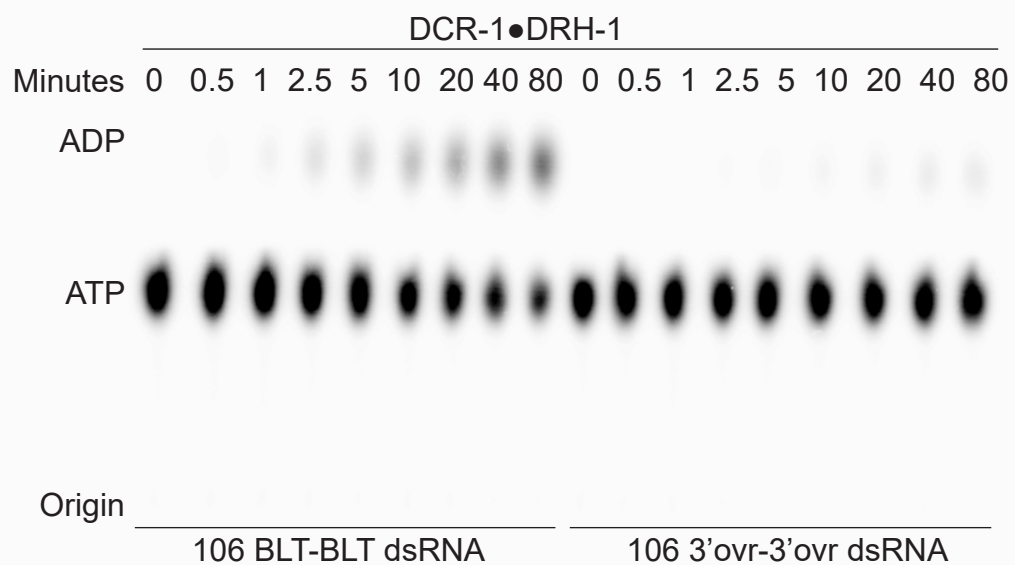

Figure 2 - source data 7: Raw digital image of thin-layer chromatography plate used in Figure 2G.

Supplement: Figure 2—source data 7. [file elife-93979-fig2-data7.zip › FIGURE 2 - SOURCE DATA 7.pdf]

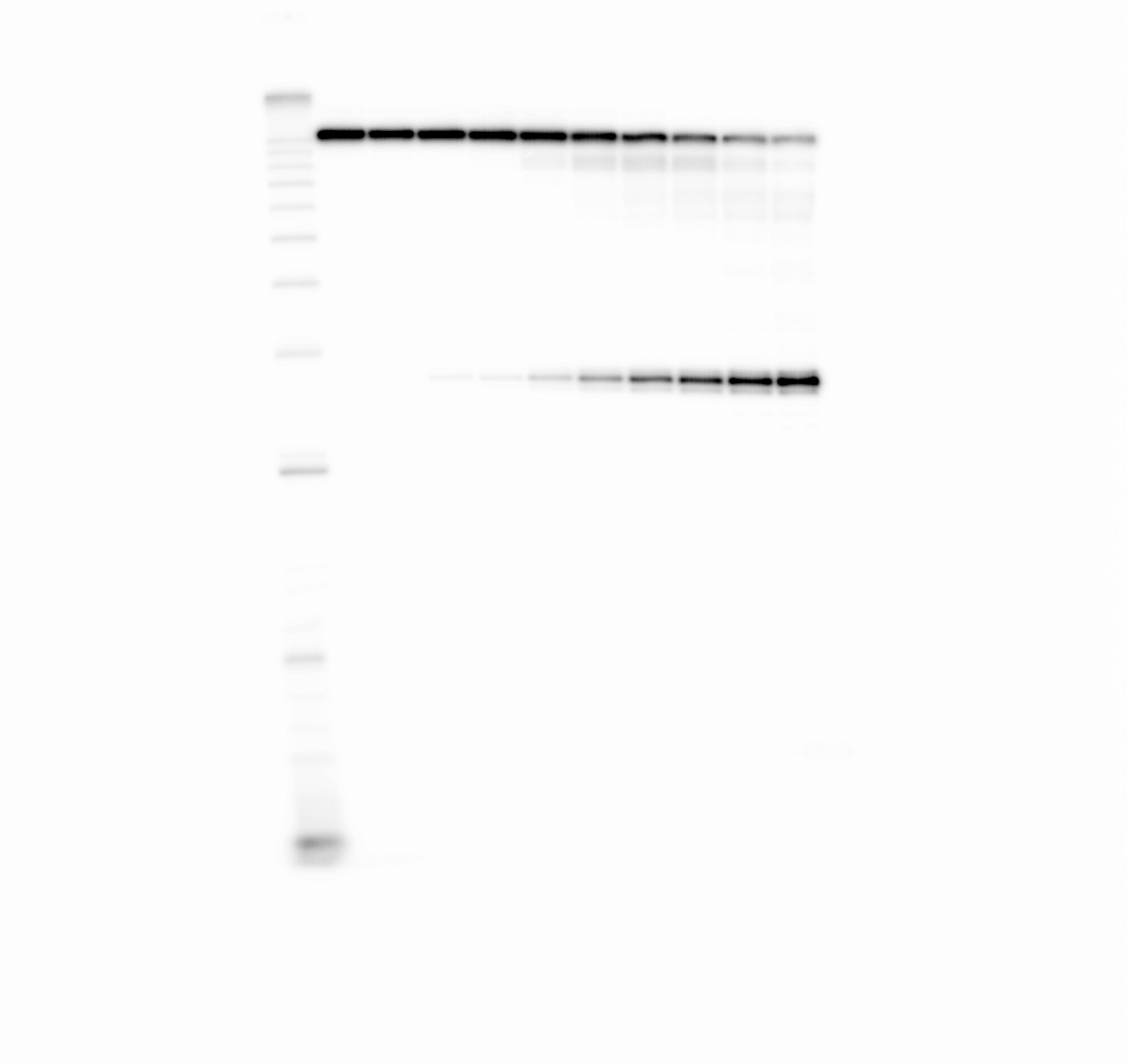

Supplement: Figure 2—figure supplement 1—source data 1. [file elife-93979-fig2-figsupp1-data1.zip › Figure 2 - figure supplement 1 - source data 1.bmp]

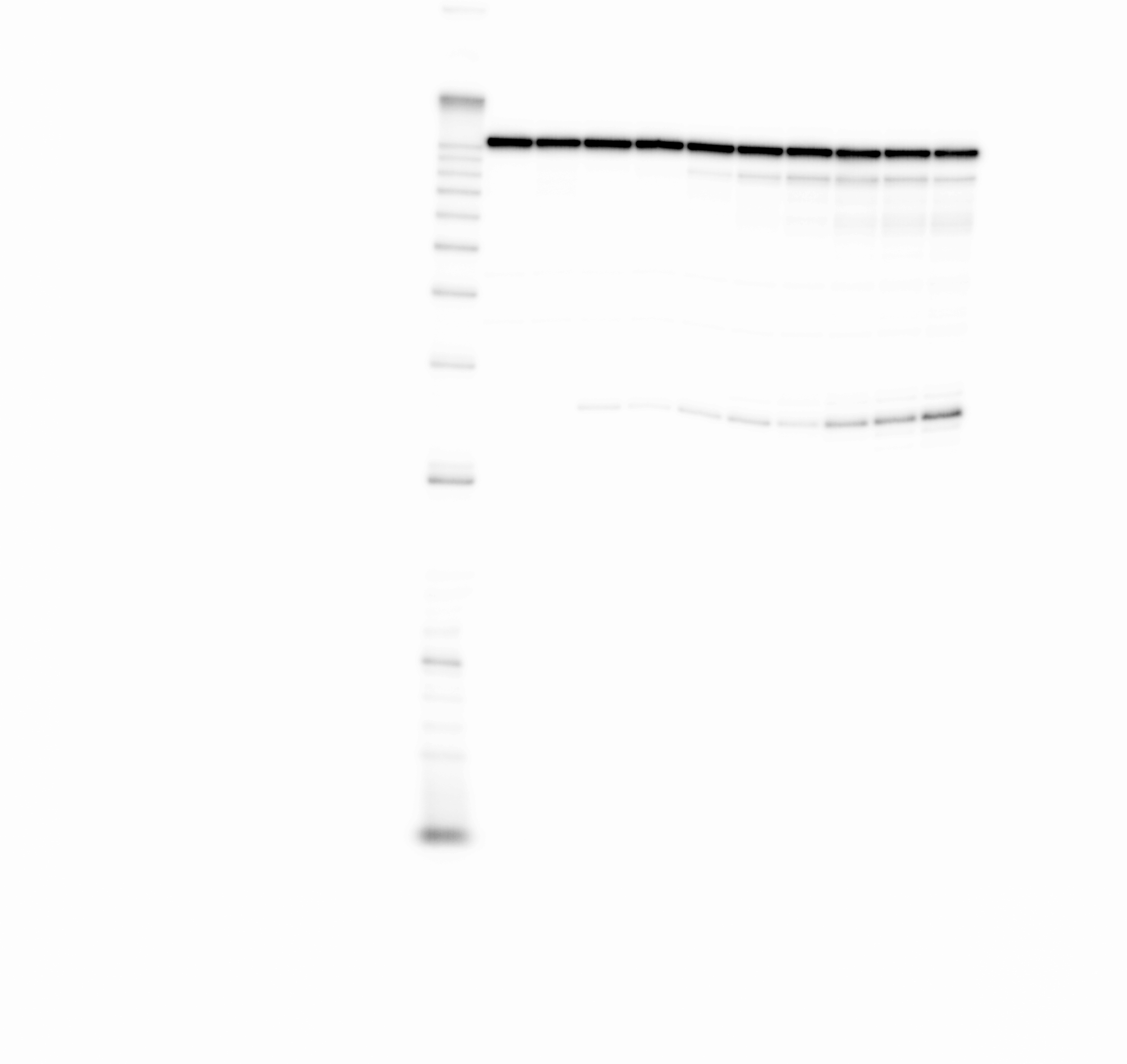

Supplement: Figure 2—figure supplement 1—source data 2. [file elife-93979-fig2-figsupp1-data2.zip › Figure 2 - figure supplement 1 - source data 2.bmp]

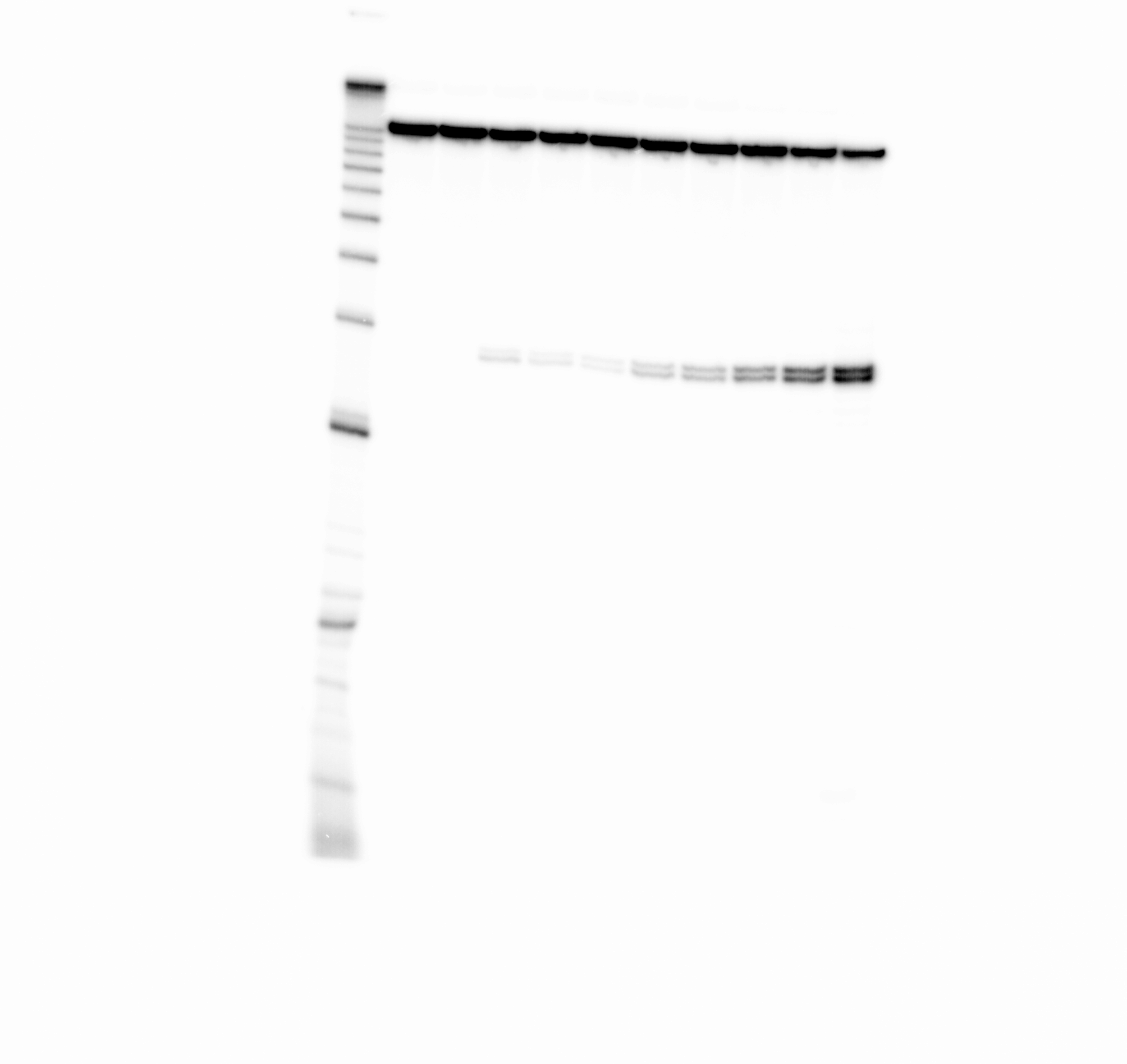

Supplement: Figure 2—figure supplement 1—source data 3. [file elife-93979-fig2-figsupp1-data3.zip › Figure 2 - figure supplement 1 - source data 3.bmp]

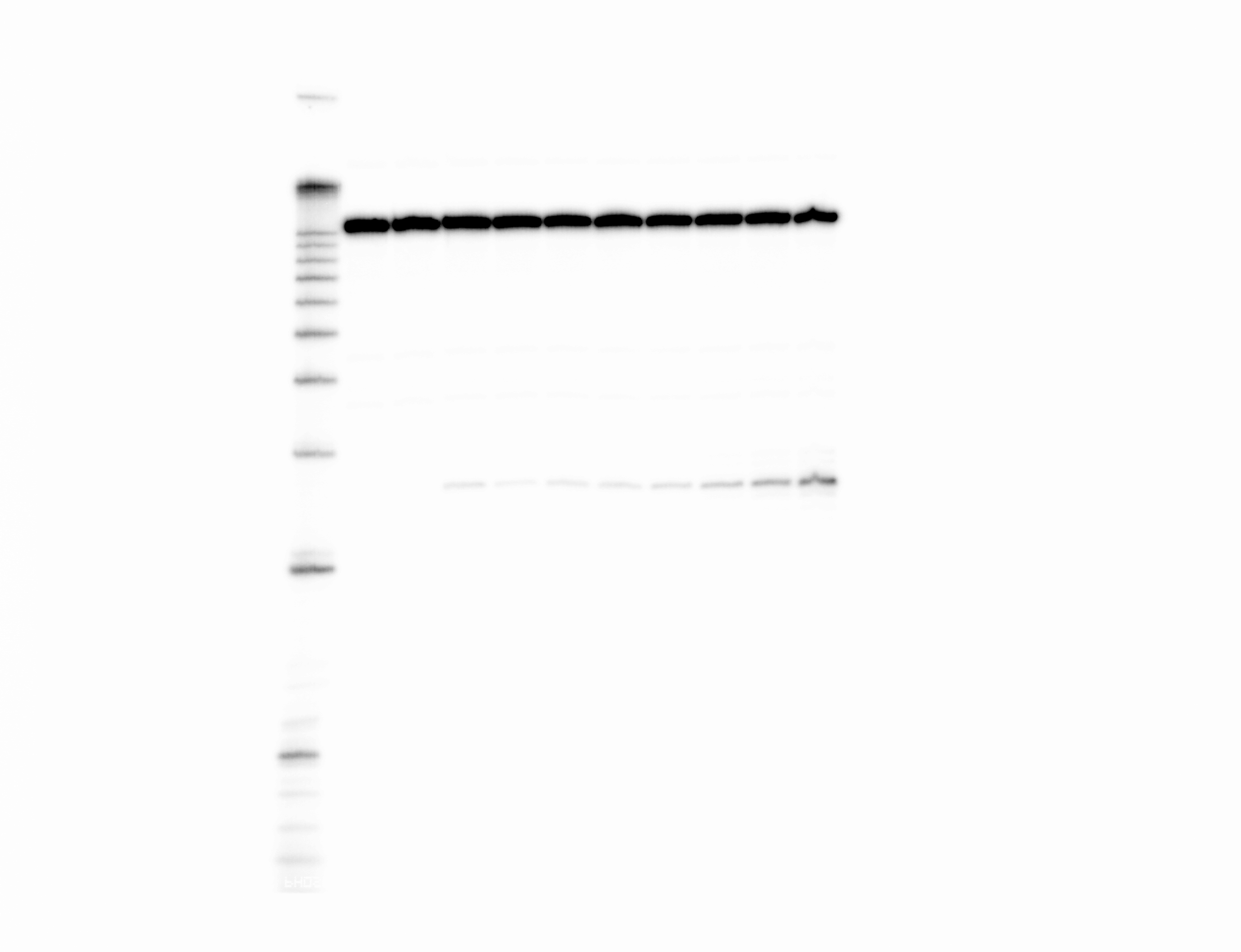

Supplement: Figure 2—figure supplement 1—source data 4. [file elife-93979-fig2-figsupp1-data4.zip › Figure 2 - figure supplement 1 - source data 4.bmp]

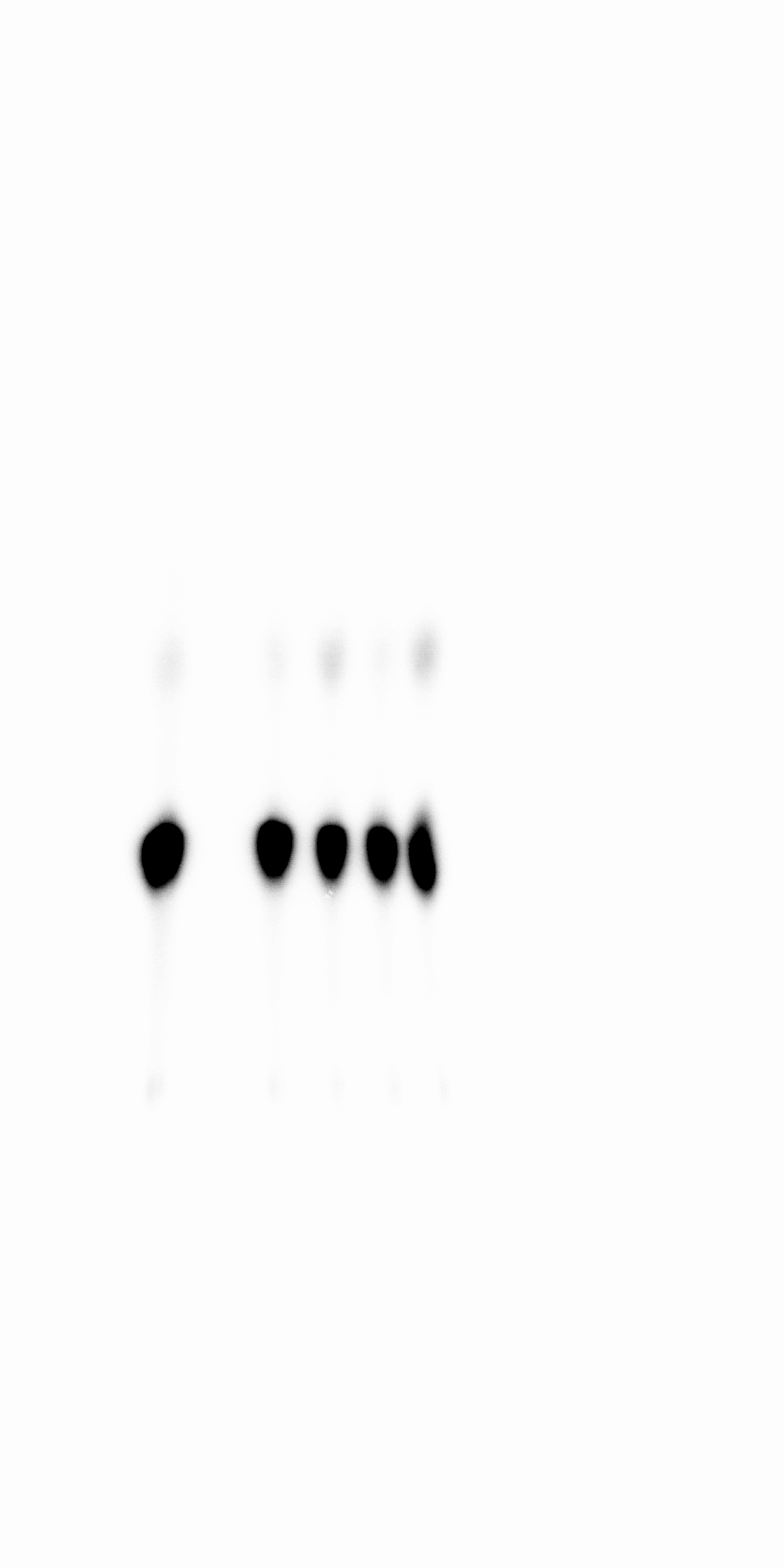

Supplement: Figure 2—figure supplement 1—source data 7. [file elife-93979-fig2-figsupp1-data7.zip › Figure 2 - figure supplement 1 - source data 7.bmp]

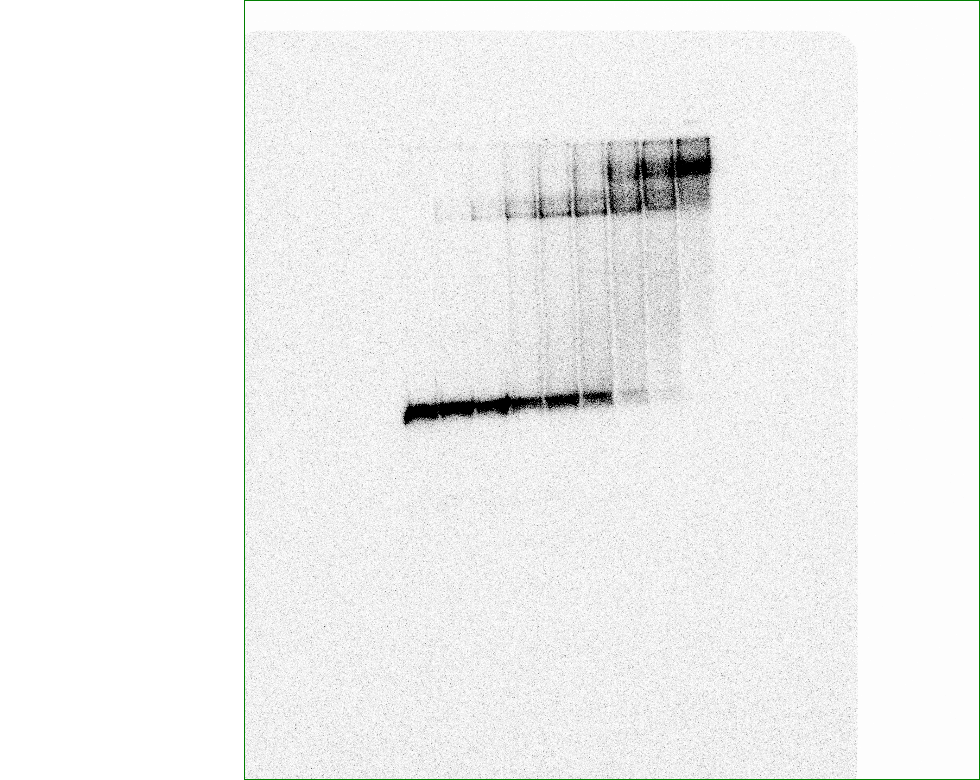

Supplement: Figure 3—source data 1. [file elife-93979-fig3-data1.zip › FIGURE 3 - SOURCE DATA 1.bmp]

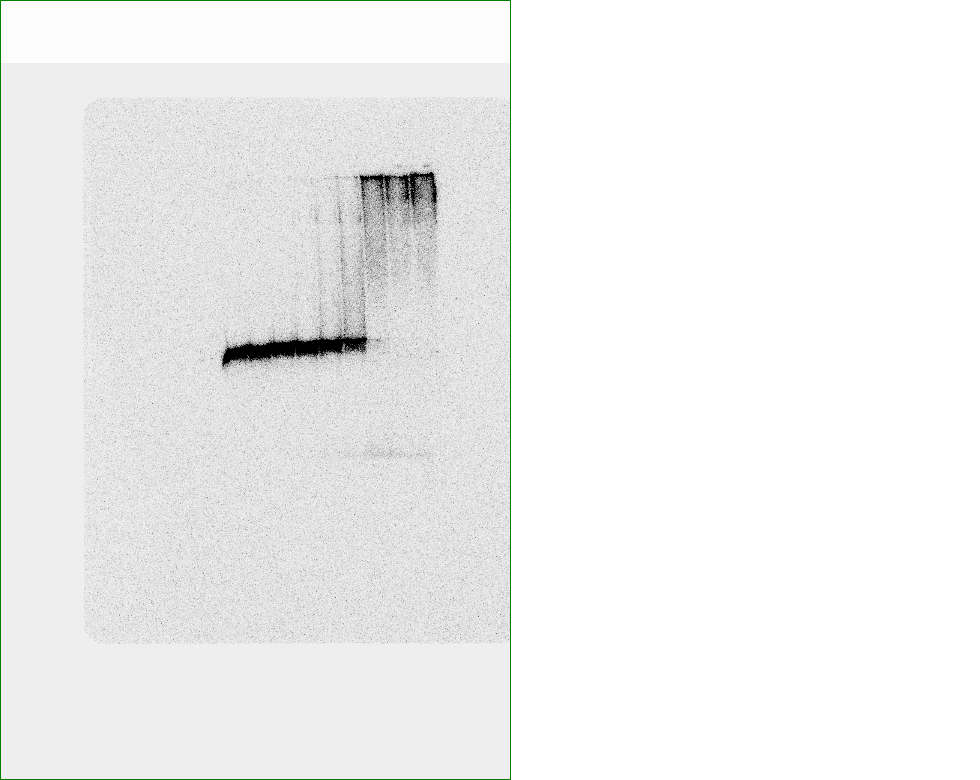

Supplement: Figure 3—source data 2. [file elife-93979-fig3-data2.zip › FIGURE 3 - SOURCE DATA 2.bmp]

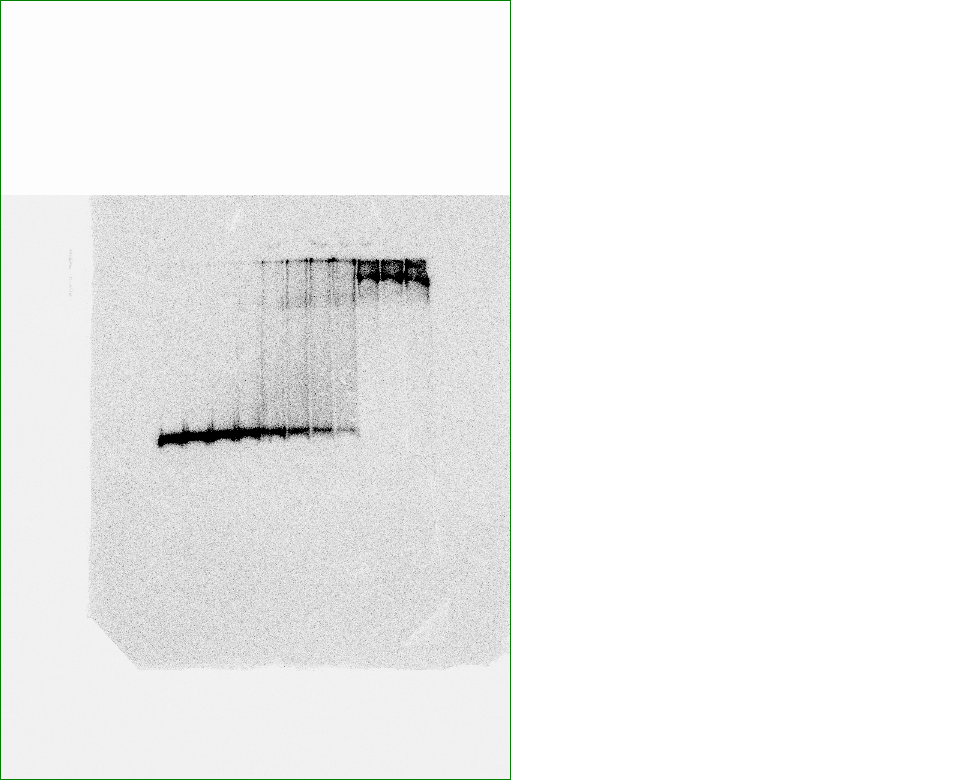

Supplement: Figure 3—source data 3. [file elife-93979-fig3-data3.zip › FIGURE 3 - SOURCE DATA 3.bmp]

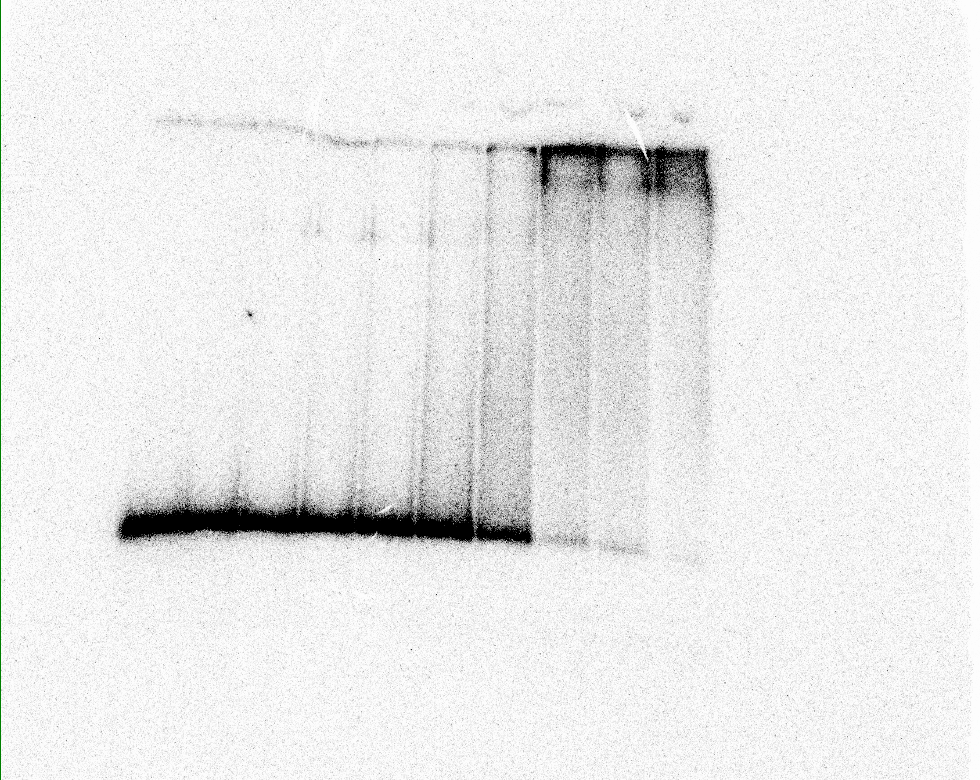

Supplement: Figure 3—source data 4. [file elife-93979-fig3-data4.zip › FIGURE 3 - SOURCE DATA 4.bmp]

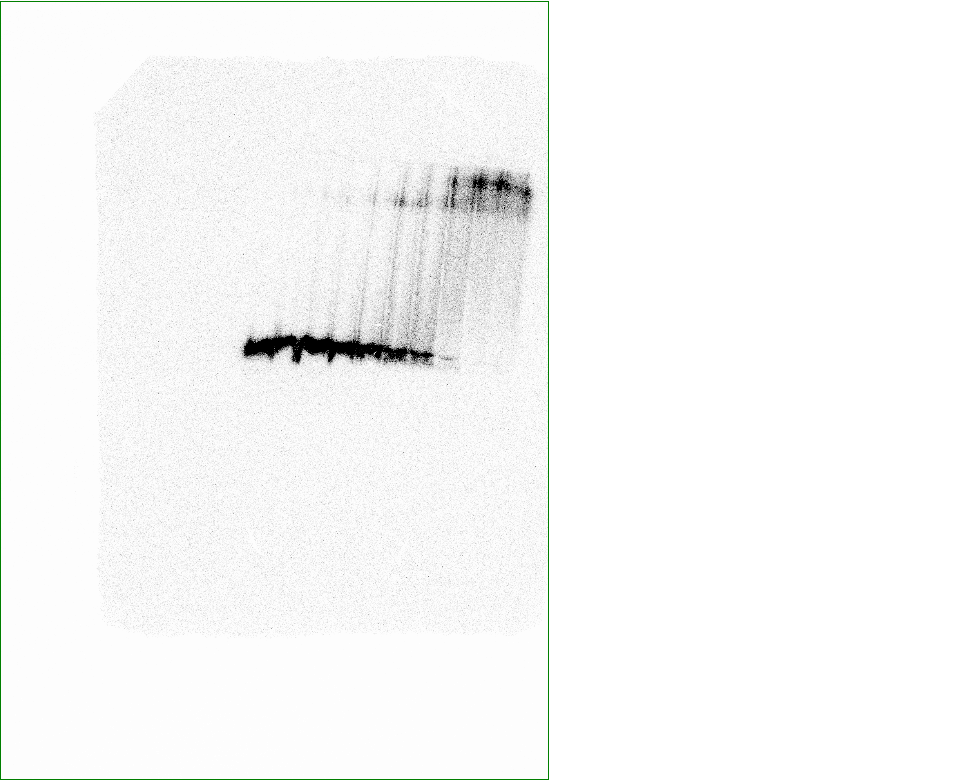

Supplement: Figure 3—source data 5. [file elife-93979-fig3-data5.zip › FIGURE 3 - SOURCE DATA 5.bmp]

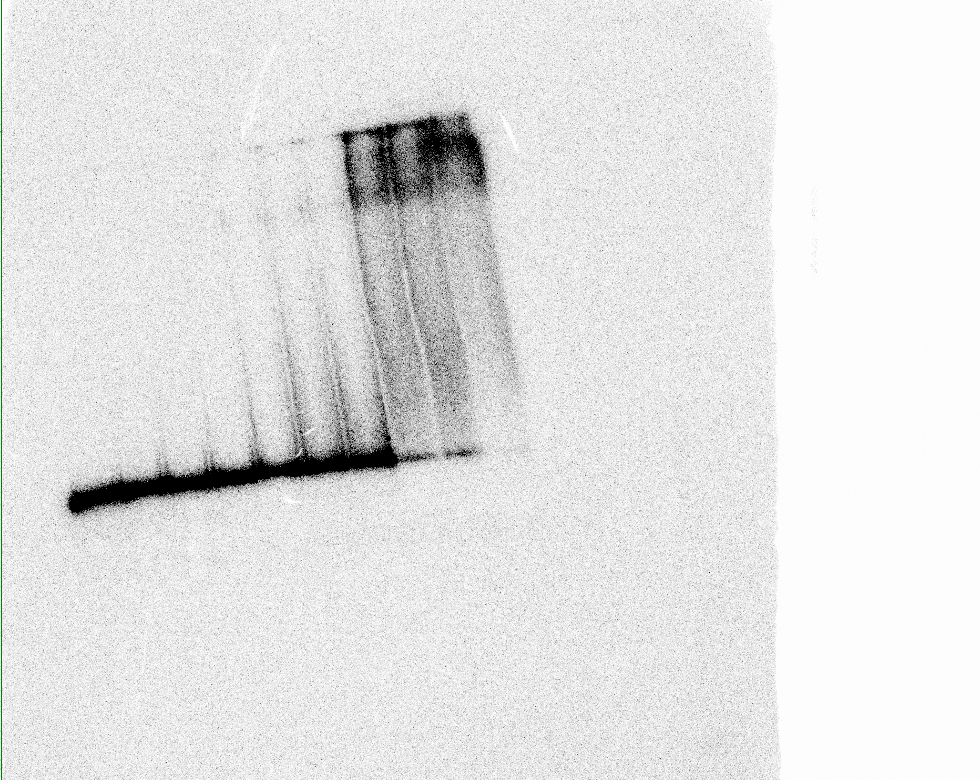

Supplement: Figure 3—source data 6. [file elife-93979-fig3-data6.zip › FIGURE 3 - SOURCE DATA 6.bmp]

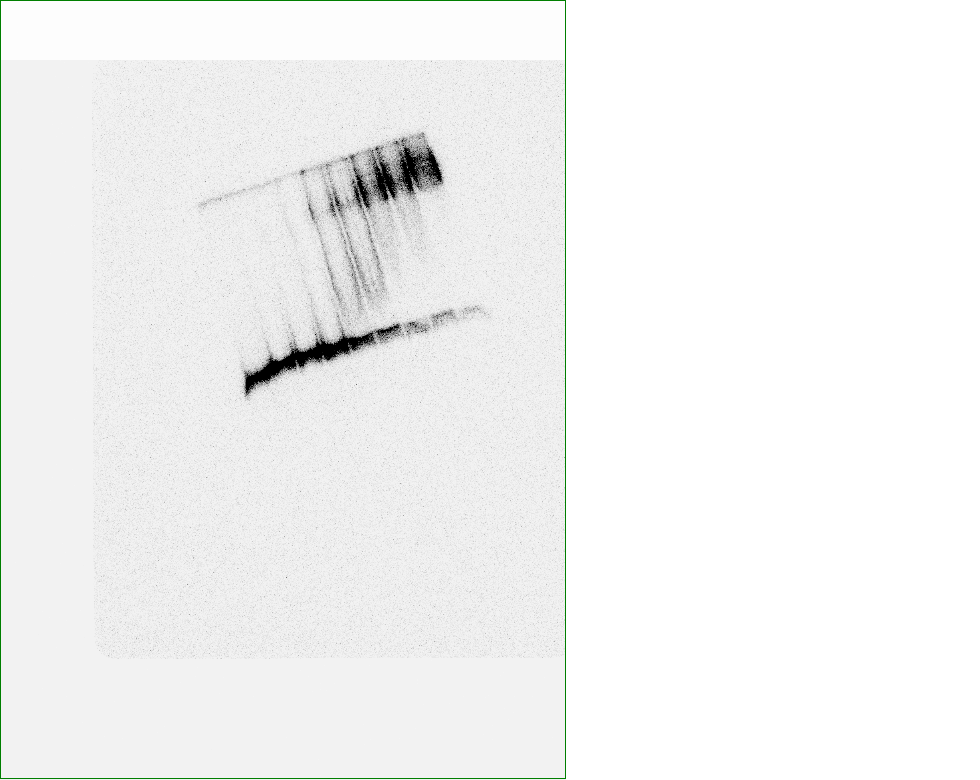

Supplement: Figure 3—source data 7. [file elife-93979-fig3-data7.zip › FIGURE 3 - SOURCE DATA 7.bmp]

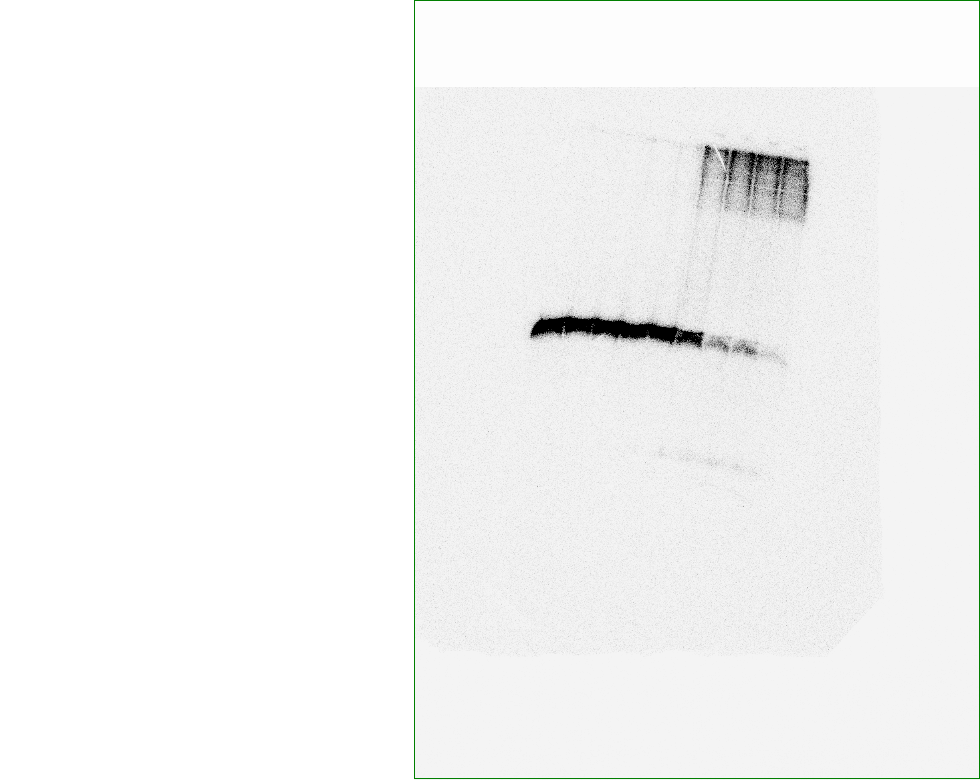

Supplement: Figure 3—source data 8. [file elife-93979-fig3-data8.zip › FIGURE 3 - SOURCE DATA 8.bmp]

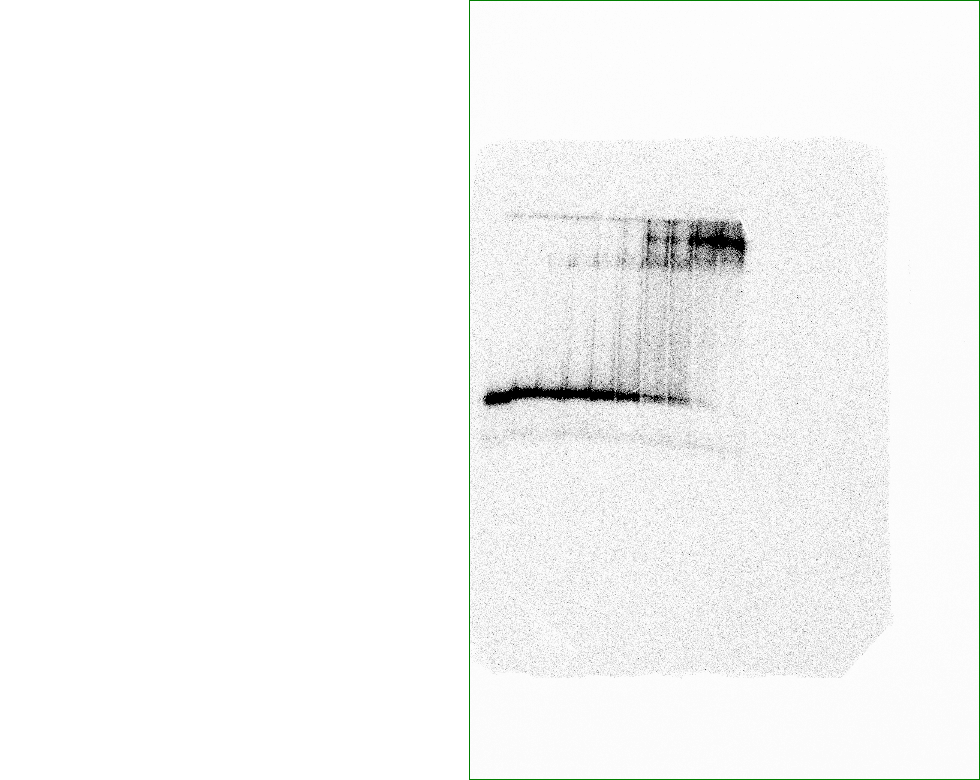

Supplement: Figure 3—figure supplement 1—source data 1. [file elife-93979-fig3-figsupp1-data1.zip › Figure 3 - figure supplement 1 - source data 1.bmp]

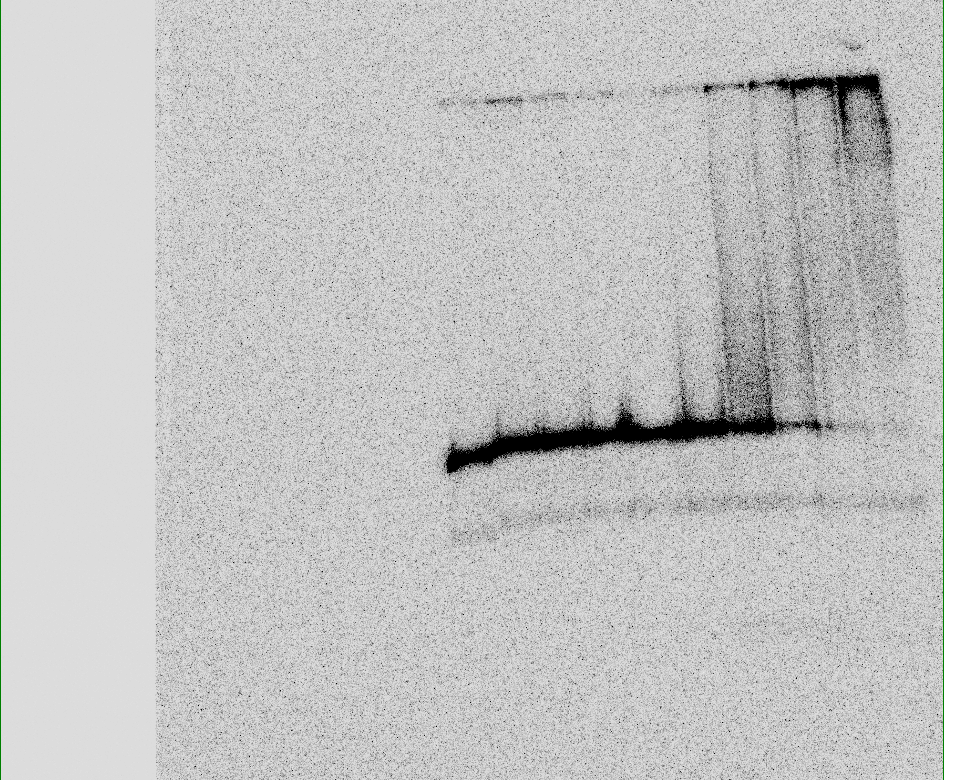

Supplement: Figure 3—figure supplement 1—source data 2. [file elife-93979-fig3-figsupp1-data2.zip › Figure 3 - figure supplement 1 - source data 2.bmp]

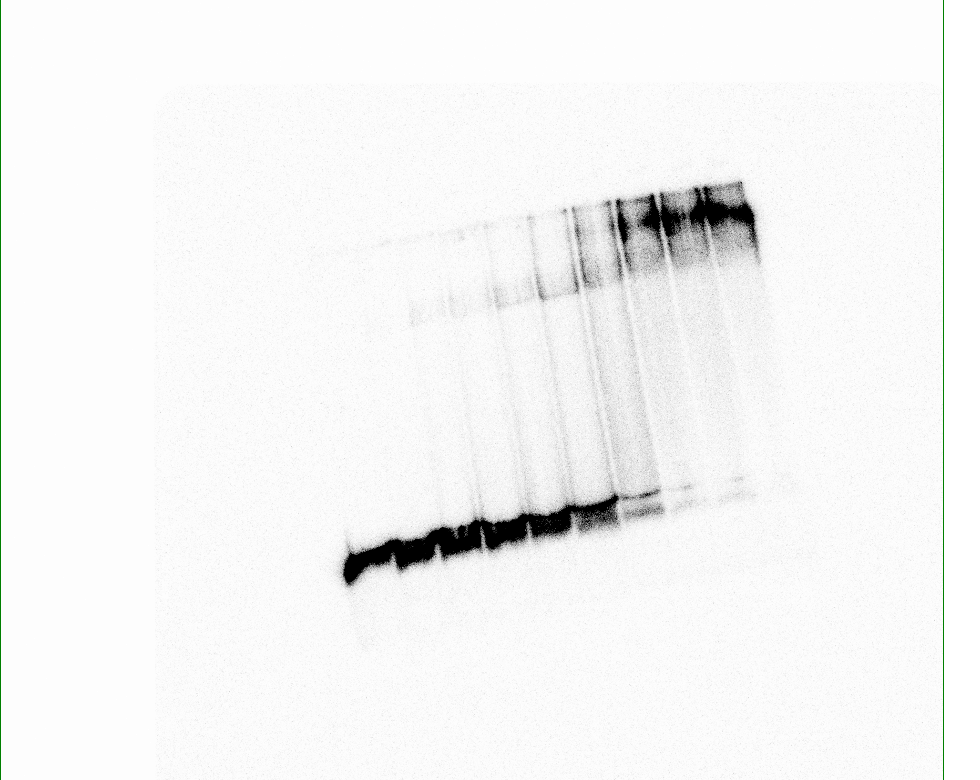

Supplement: Figure 3—figure supplement 1—source data 3. [file elife-93979-fig3-figsupp1-data3.zip › Figure 3 - figure supplement 1 - source data 3.bmp]

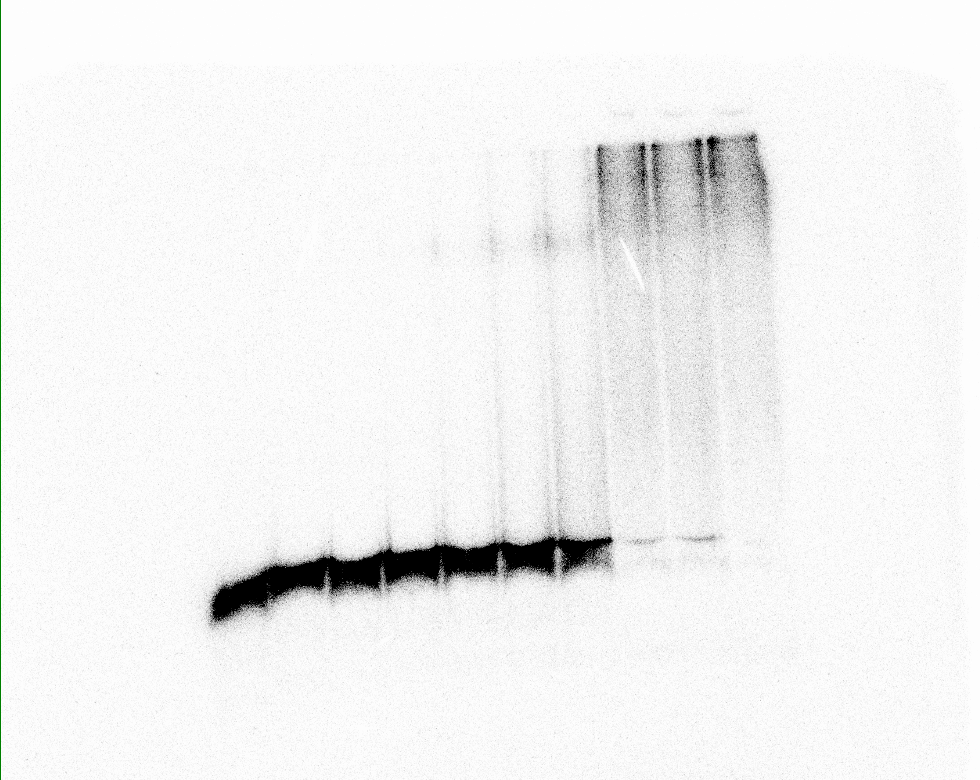

Supplement: Figure 3—figure supplement 1—source data 4. [file elife-93979-fig3-figsupp1-data4.zip › Figure 3 - figure supplement 1 - source data 4.bmp]

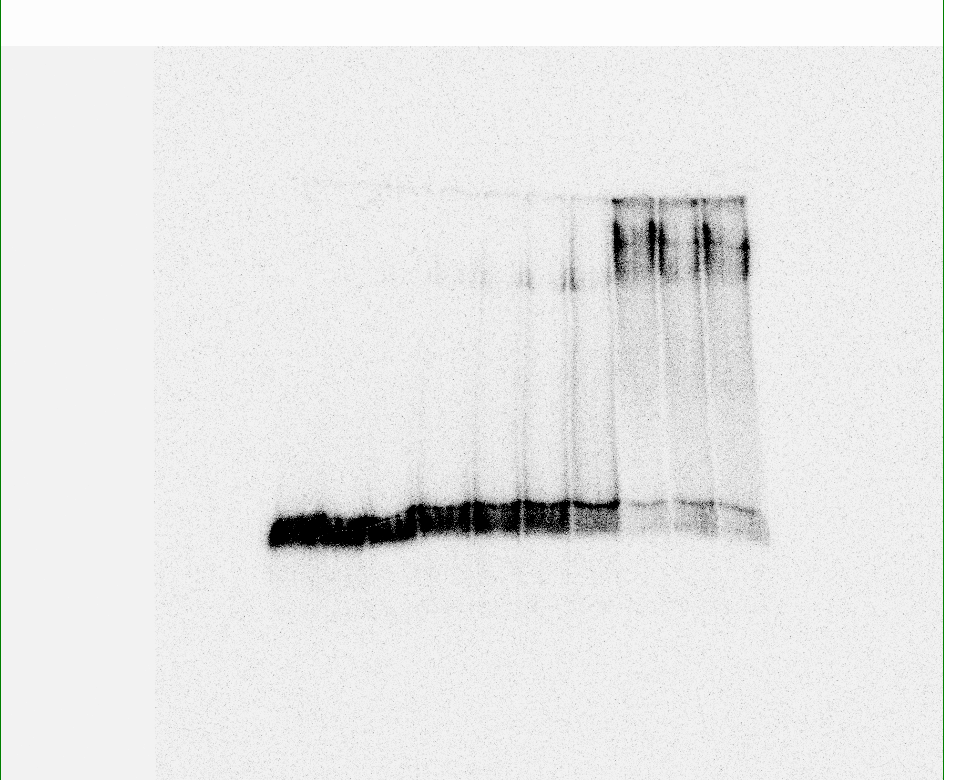

Supplement: Figure 3—figure supplement 1—source data 5. [file elife-93979-fig3-figsupp1-data5.zip › Figure 3 - figure supplement 1 - source data 5.bmp]

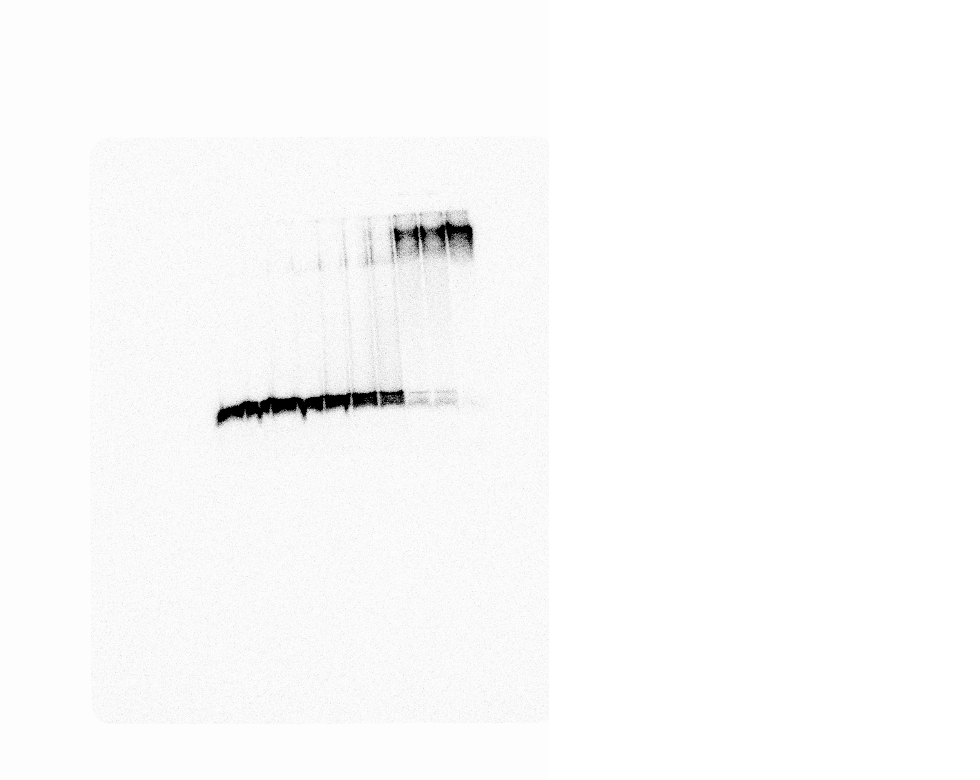

Supplement: Figure 3—figure supplement 1—source data 6. [file elife-93979-fig3-figsupp1-data6.zip › Figure 3 - figure supplement 1 - source data 6.bmp]

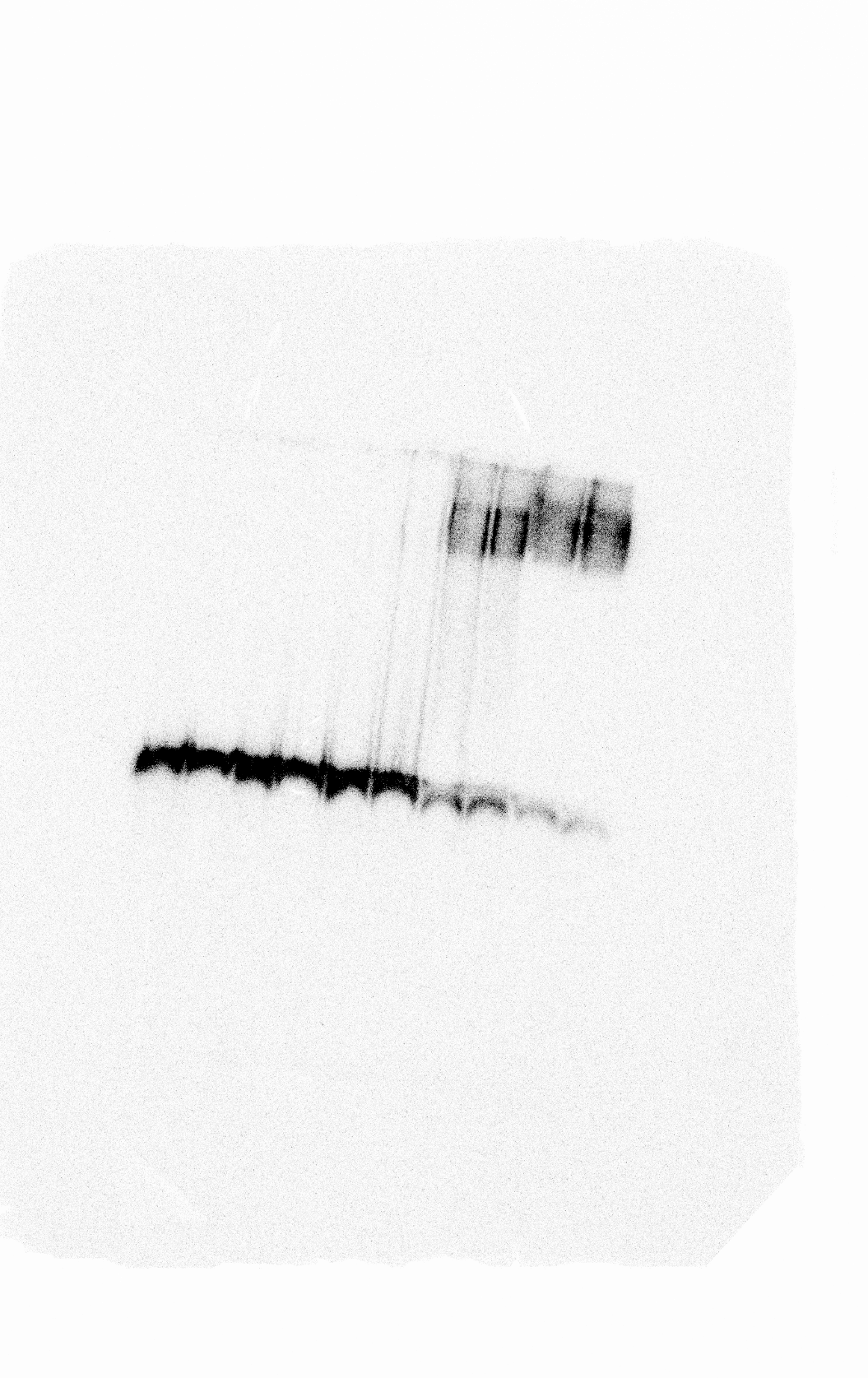

Supplement: Figure 3—figure supplement 1—source data 7. [file elife-93979-fig3-figsupp1-data7.zip › Figure 3 - figure supplement 1 - source data 7.bmp]

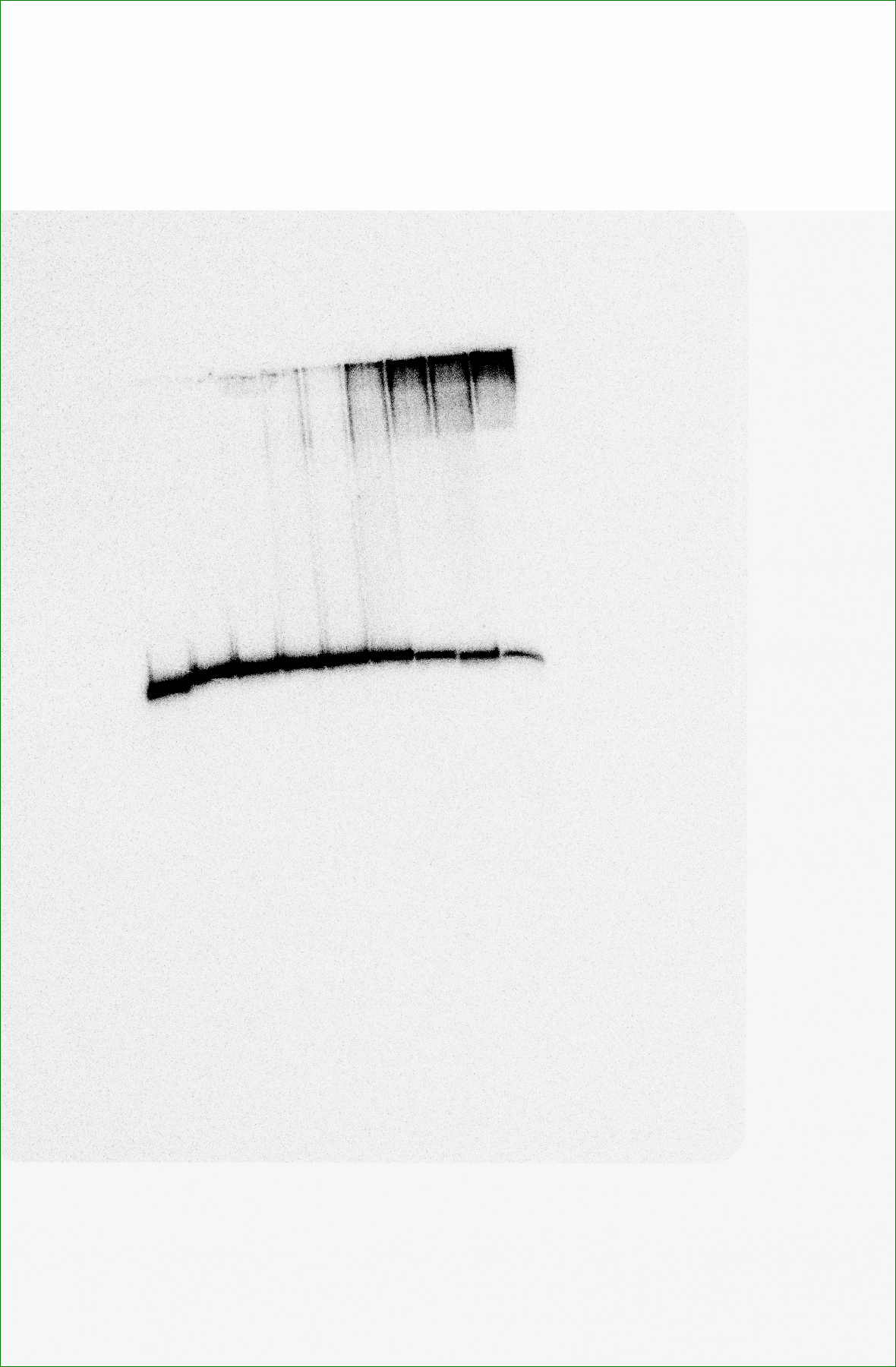

Supplement: Figure 3—figure supplement 1—source data 8. [file elife-93979-fig3-figsupp1-data8.zip › Figure 3 - figure supplement 1 - source data 8.bmp]

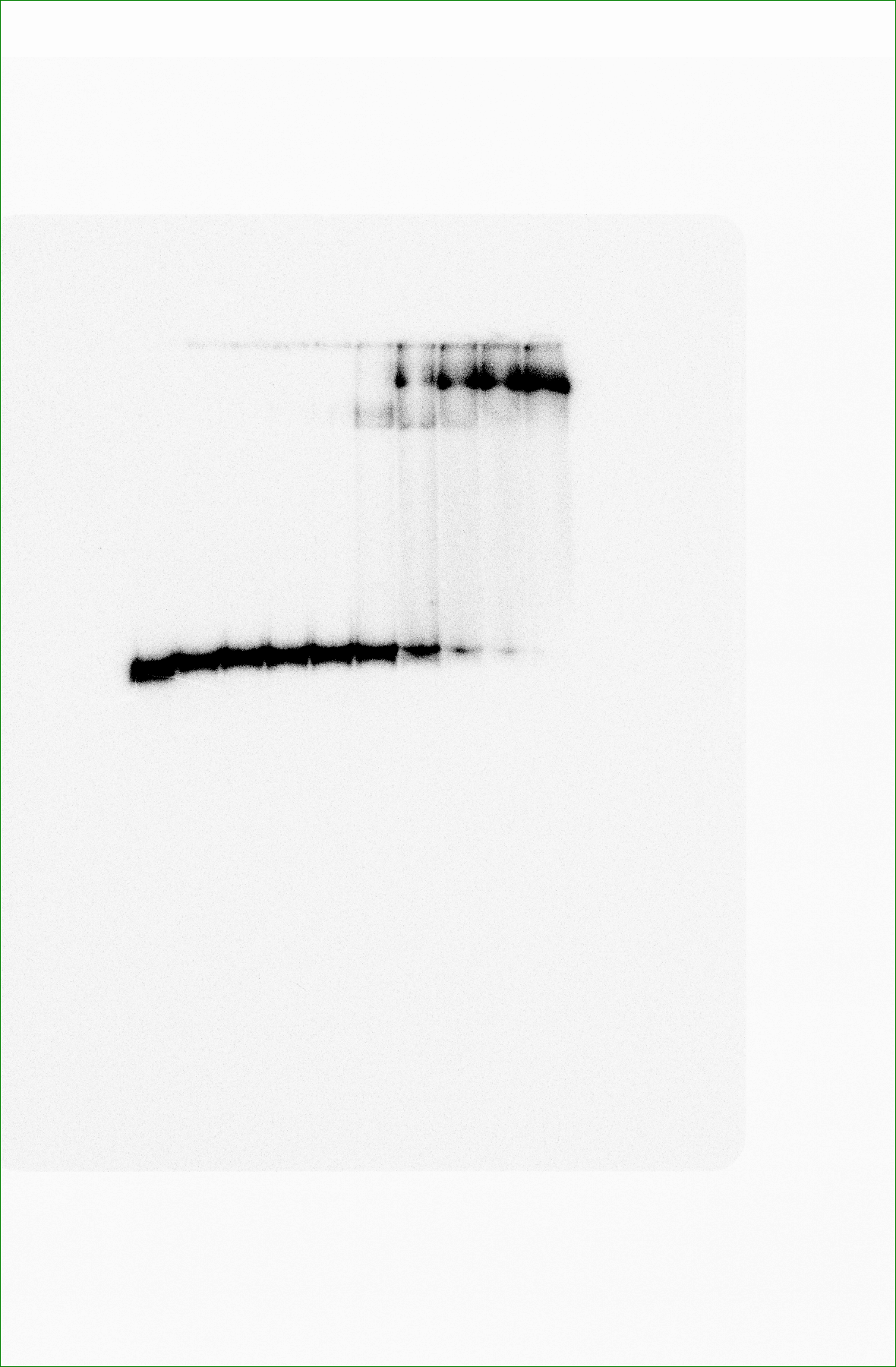

Supplement: Figure 3—figure supplement 2—source data 1. [file elife-93979-fig3-figsupp2-data1.zip › Figure 3 - figure supplement 2 - source data 1.bmp]

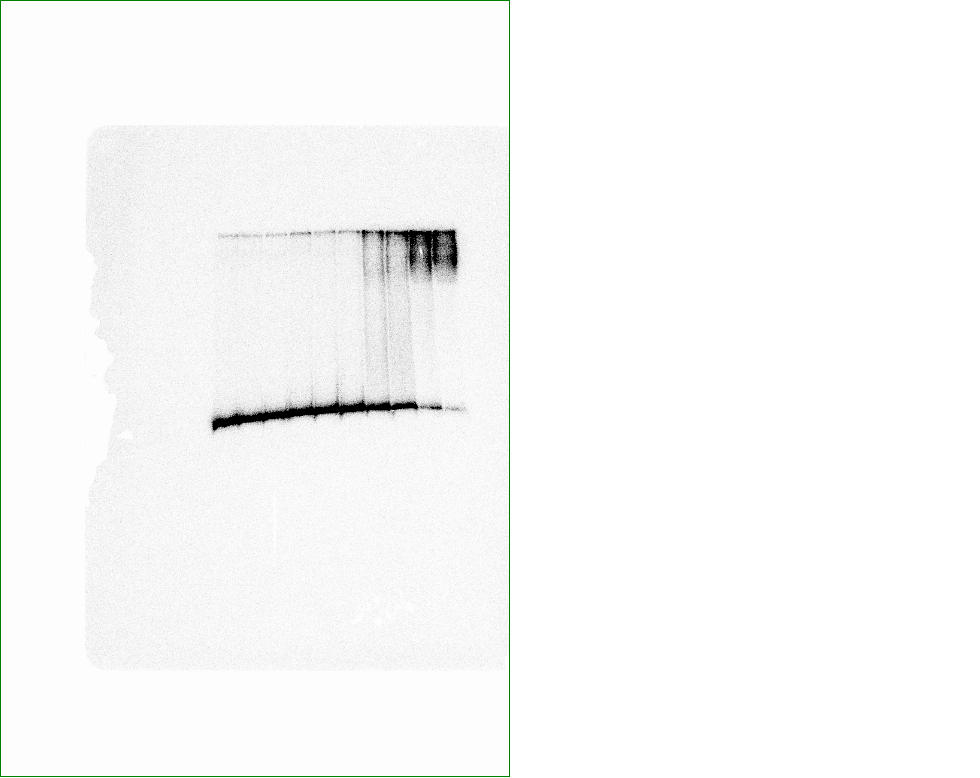

Supplement: Figure 3—figure supplement 2—source data 2. [file elife-93979-fig3-figsupp2-data2.zip › Figure 3 - figure supplement 2 - source data 2.bmp]

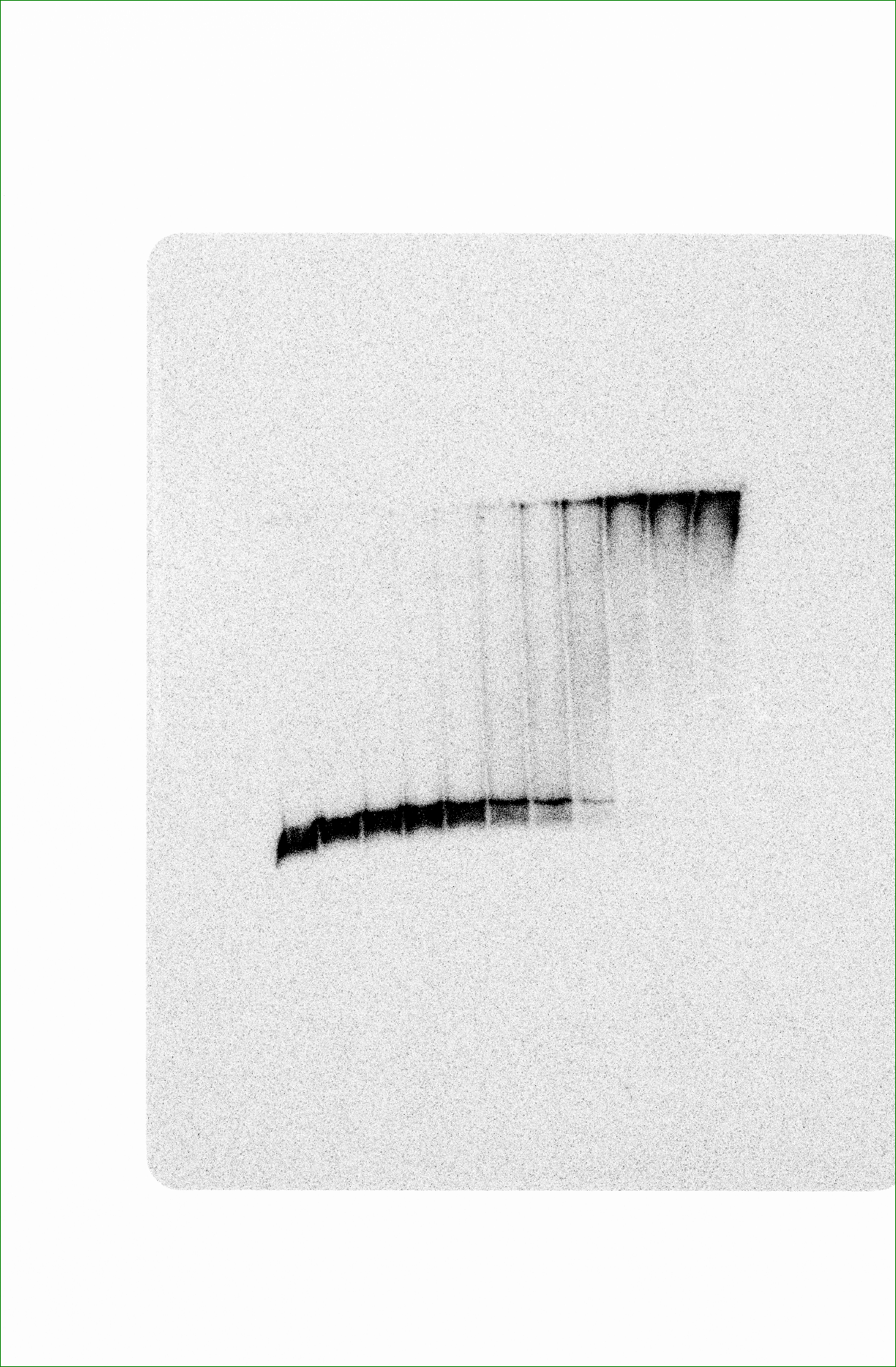

Supplement: Figure 3—figure supplement 2—source data 3. [file elife-93979-fig3-figsupp2-data3.zip › Figure 3 - figure supplement 2 - source data 3.bmp]

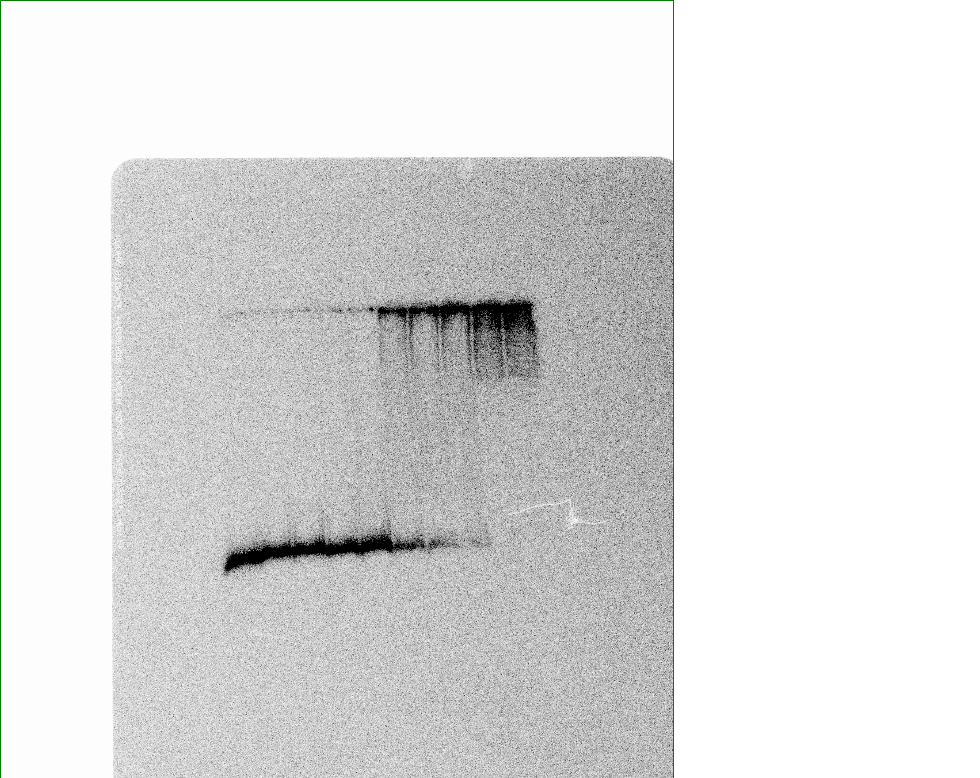

Supplement: Figure 3—figure supplement 2—source data 4. [file elife-93979-fig3-figsupp2-data4.zip › Figure 3 - figure supplement 2 - source data 4.bmp]

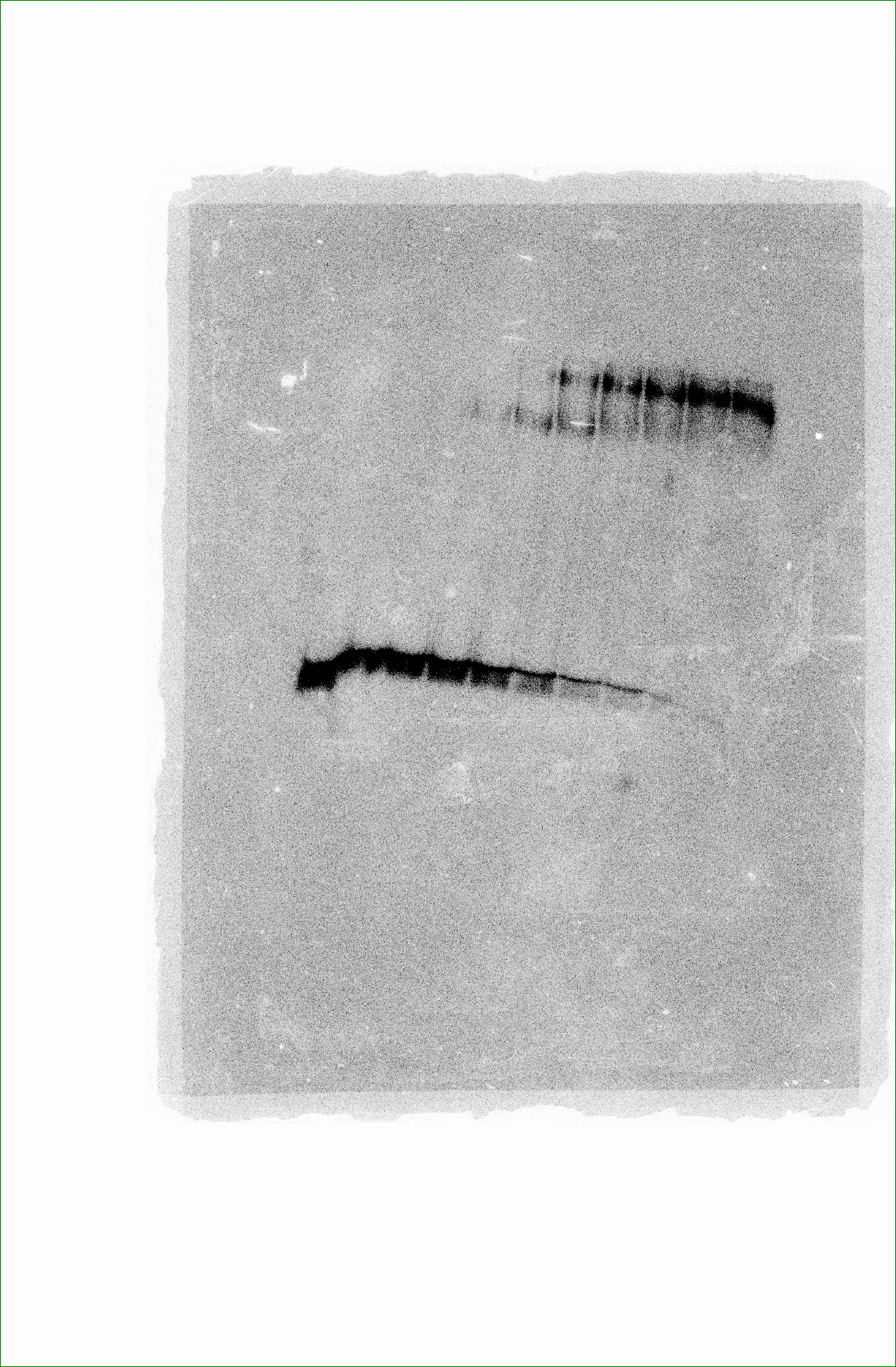

Supplement: Figure 3—figure supplement 2—source data 5. [file elife-93979-fig3-figsupp2-data5.zip › Figure 3 - figure supplement 2 - source data 5.bmp]

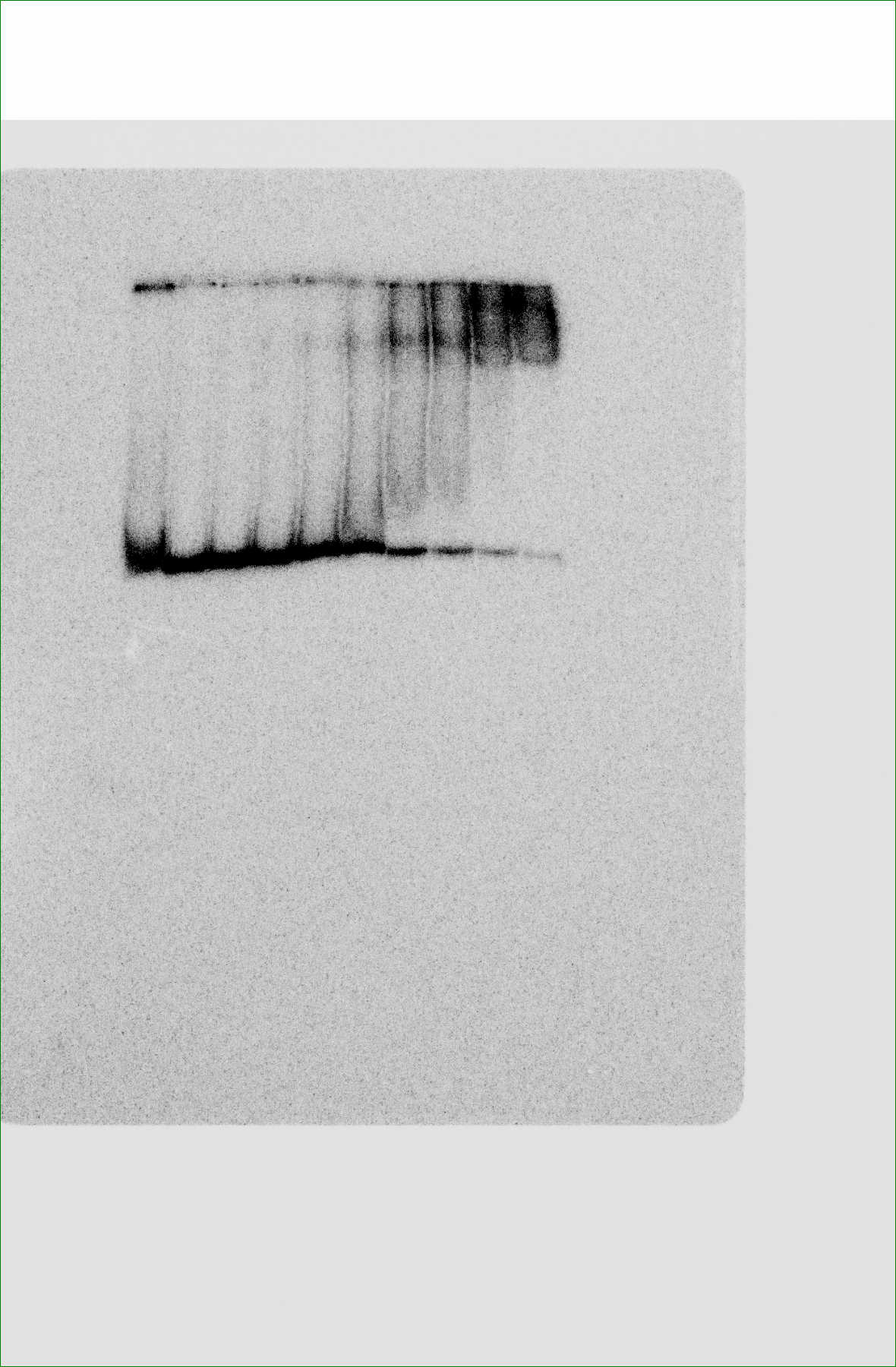

Supplement: Figure 3—figure supplement 2—source data 6. [file elife-93979-fig3-figsupp2-data6.zip › Figure 3 - figure supplement 2 - source data 6.bmp]

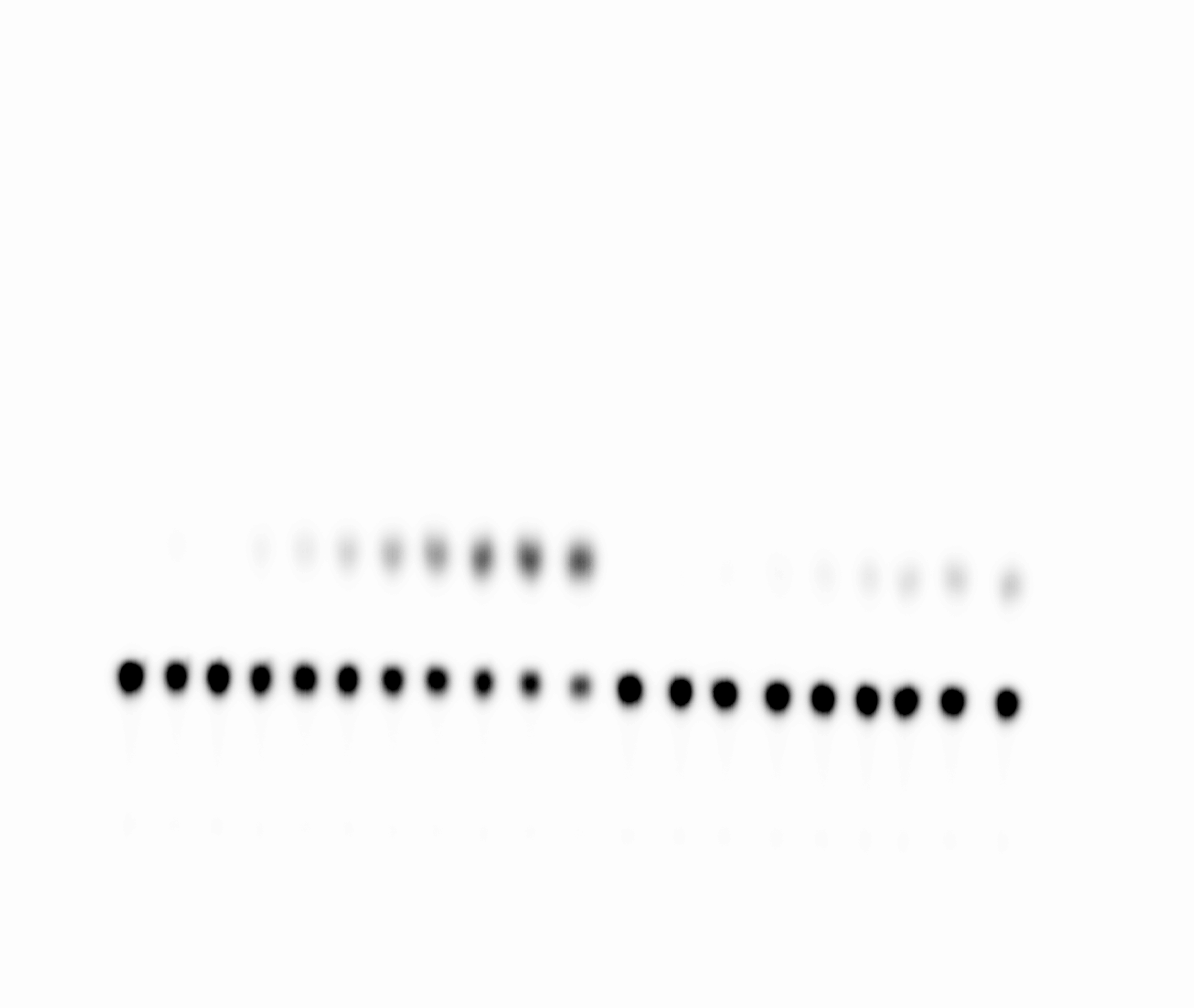

Supplement: Figure 5—source data 1. [file elife-93979-fig5-data1.zip › FIGURE 5 - SOURCE DATA 1.bmp]

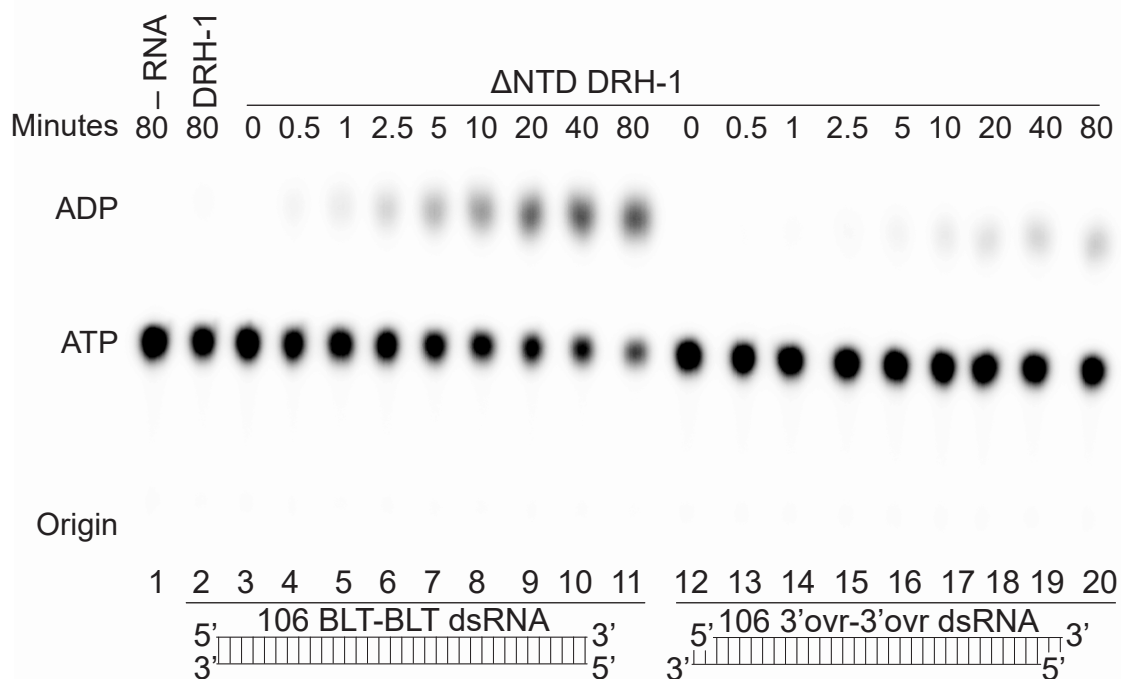

Figure 5 - source data 1: Raw digital image of thin-layer chromatography plate used in Figure 5A.

Supplement: Figure 5—source data 1. [file elife-93979-fig5-data1.zip › FIGURE 5 - SOURCE DATA 1.pdf]

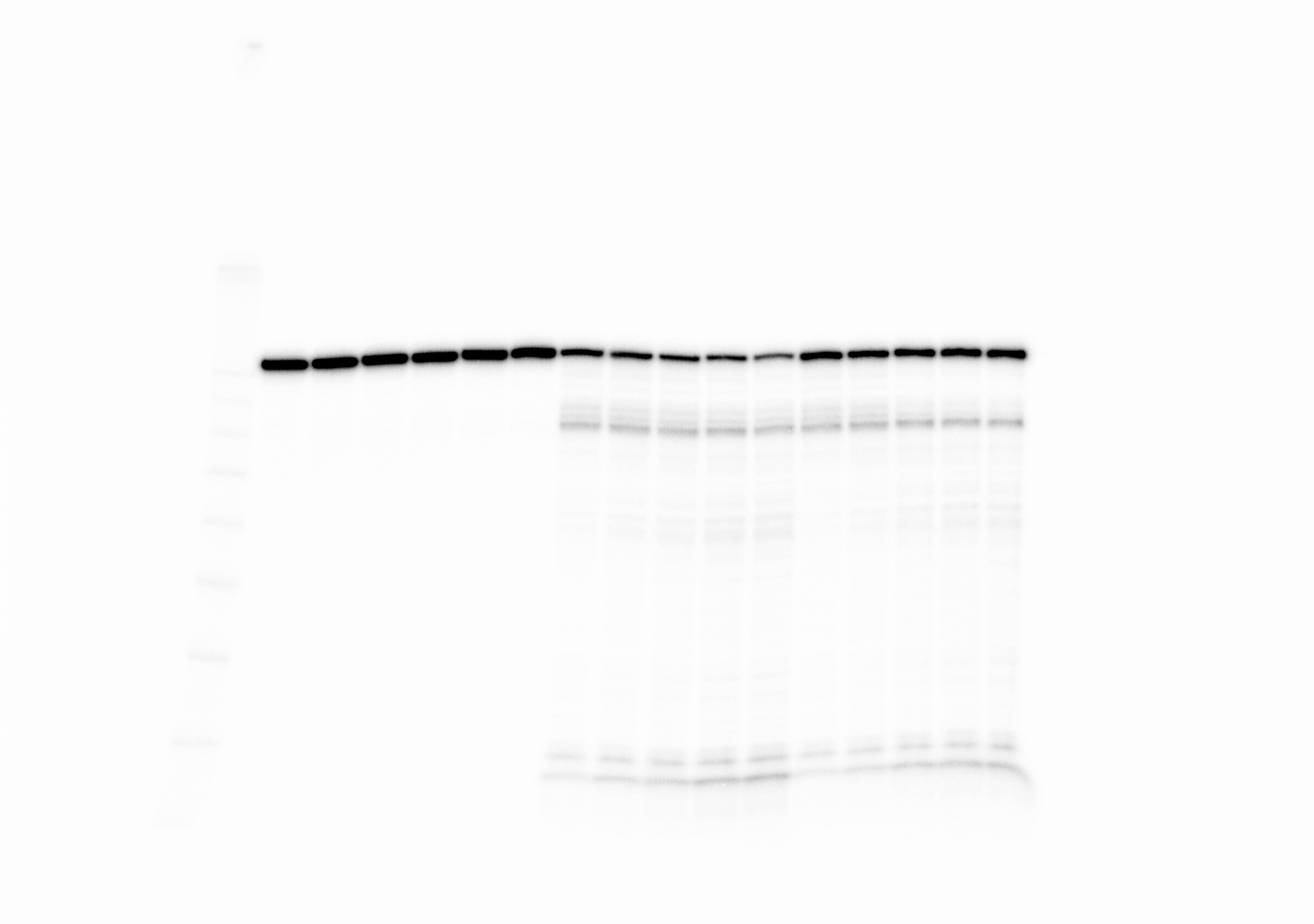

Supplement: Figure 5—source data 2. [file elife-93979-fig5-data2.zip › FIGURE 5 - SOURCE DATA 2.bmp]

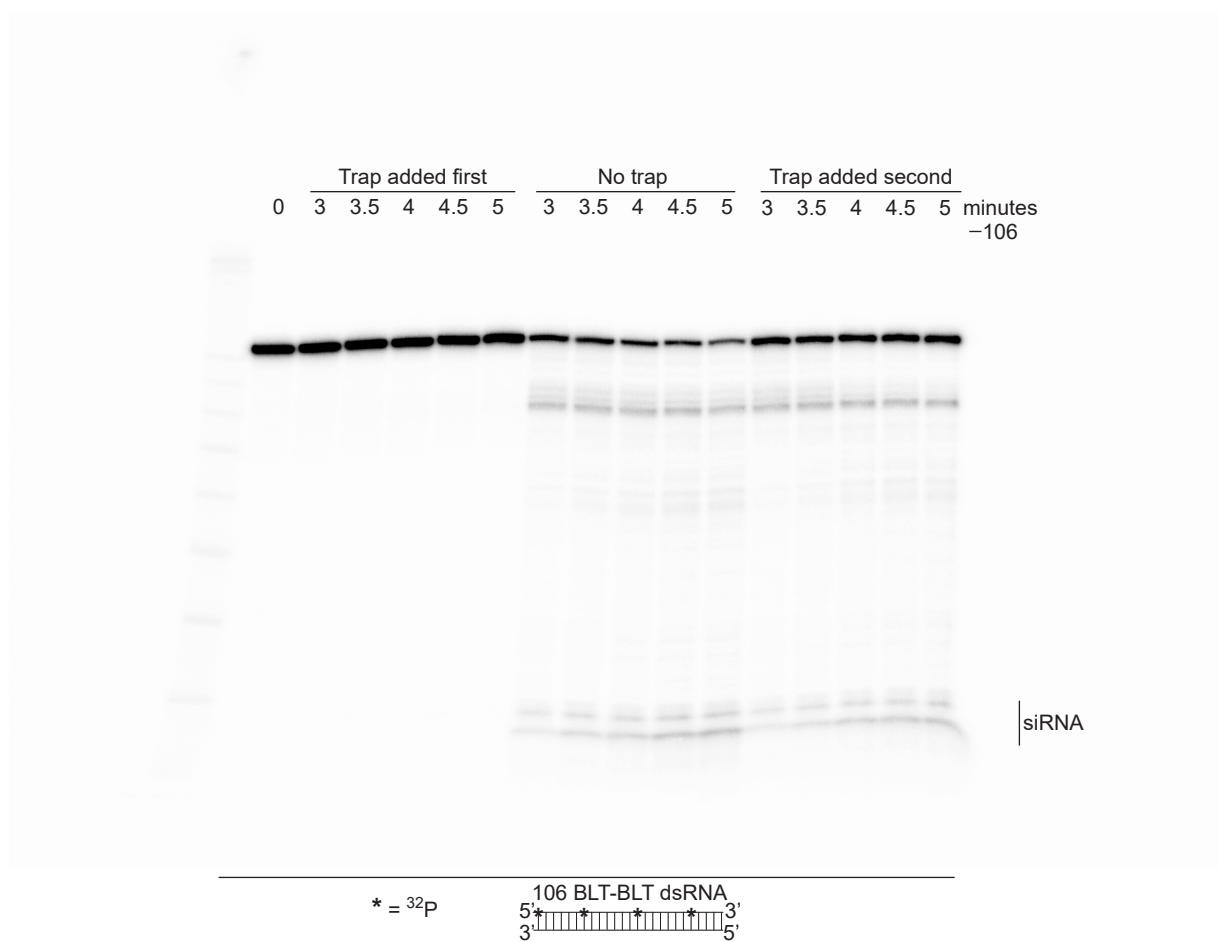

Figure 5 - source data 2: Raw digital image of cleavage phosphorimager plate used in Figure 5C.

Supplement: Figure 5—source data 2. [file elife-93979-fig5-data2.zip › FIGURE 5 - SOURCE DATA 2.pdf]

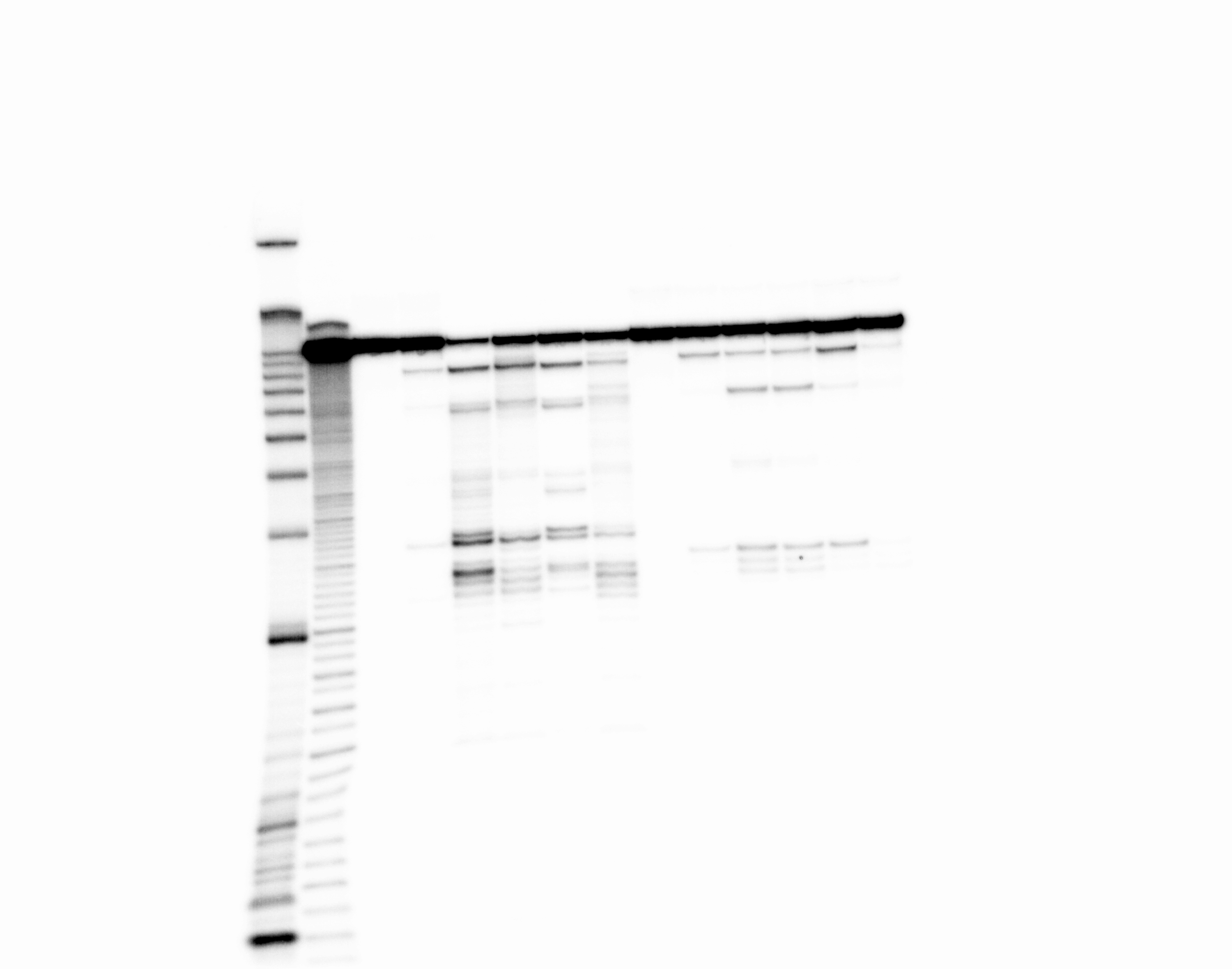

Supplement: Figure 5—source data 3. [file elife-93979-fig5-data3.zip › FIGURE 5 - SOURCE DATA 3.bmp]

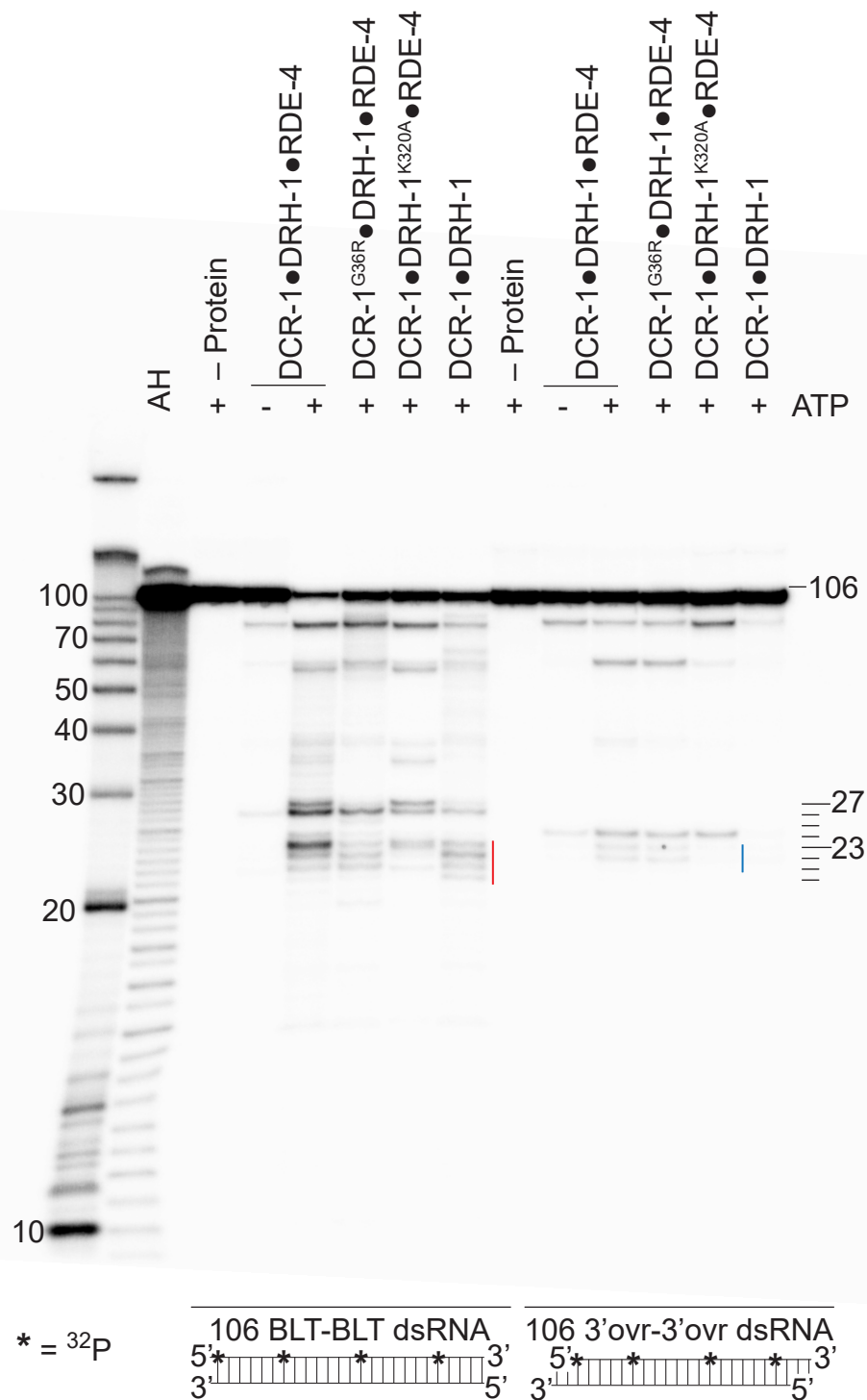

Figure 5 - source data 3: Raw digital image of cleavage phosphorimager plate used in Figure 5E.

Supplement: Figure 5—source data 3. [file elife-93979-fig5-data3.zip › FIGURE 5 - SOURCE DATA 3.pdf]
