# Supplementary material for: Caenorhabditis elegans Dicer acts with the RIG-I-like helicase DRH-1 and RDE-4 to cleave dsRNA
Source: eLife. 2024 May 15;13:RP93979. doi: 10.7554/eLife.93979 (PMC11095941; doi:10.7554/eLife.93979)
Supplement: Figure 1—figure supplement 1—source data 3. [file elife-93979-fig1-figsupp1-data3.zip › Figure 1 - figure supplement 1 - source data 3.pdf]

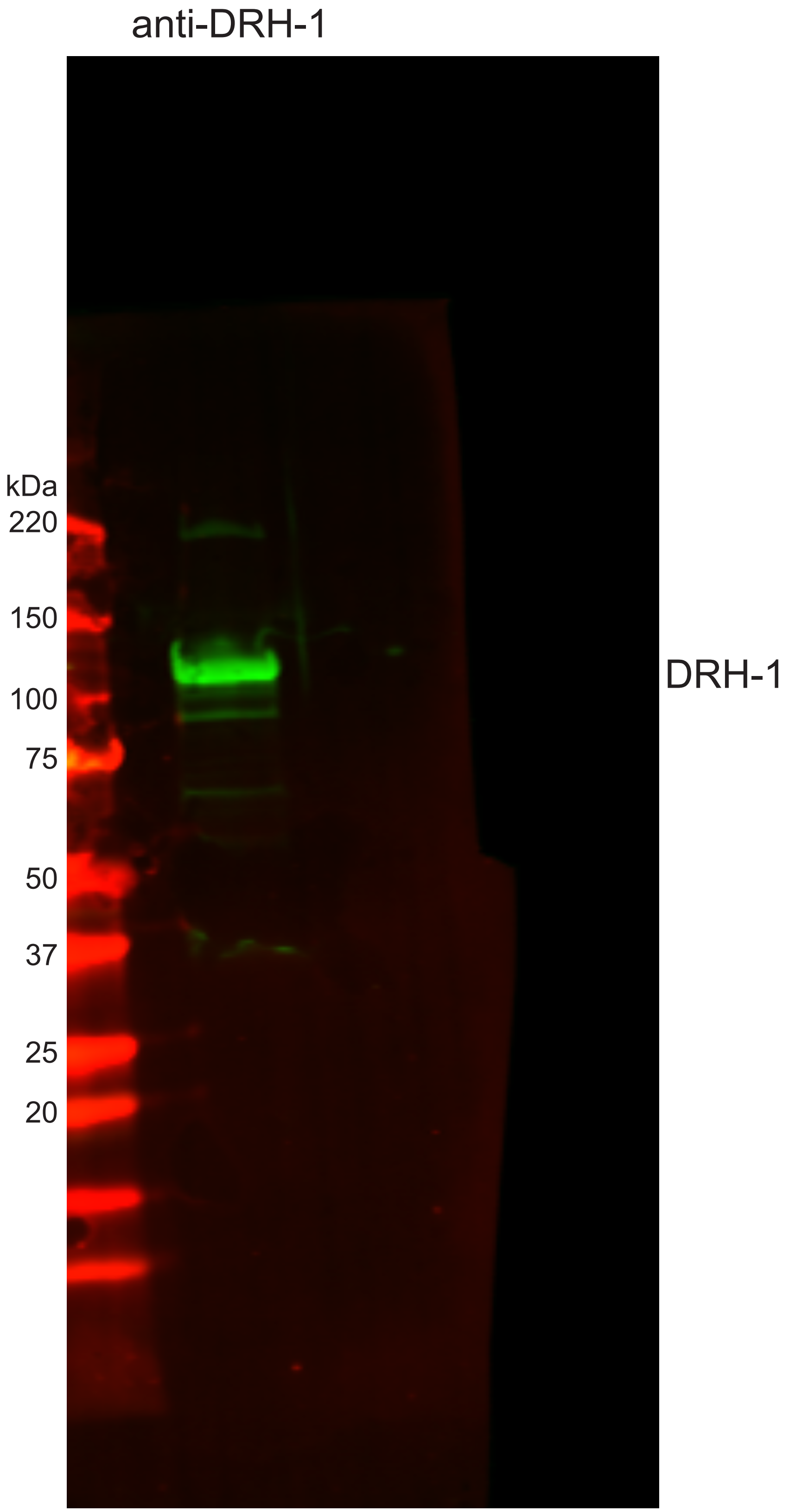

Figure 1 - figure supplement 1 - source data 3: Raw digital image of Western blot used in Figure 1 - figure supplement 1B, middle panel.
