# Supplementary material for: Caenorhabditis elegans Dicer acts with the RIG-I-like helicase DRH-1 and RDE-4 to cleave dsRNA
Source: eLife. 2024 May 15;13:RP93979. doi: 10.7554/eLife.93979 (PMC11095941; doi:10.7554/eLife.93979)
Supplement: Figure 1—figure supplement 2—source data 2. [file elife-93979-fig1-figsupp2-data2.zip › Figure 1 - figure supplement 2 - source data 2.pdf]

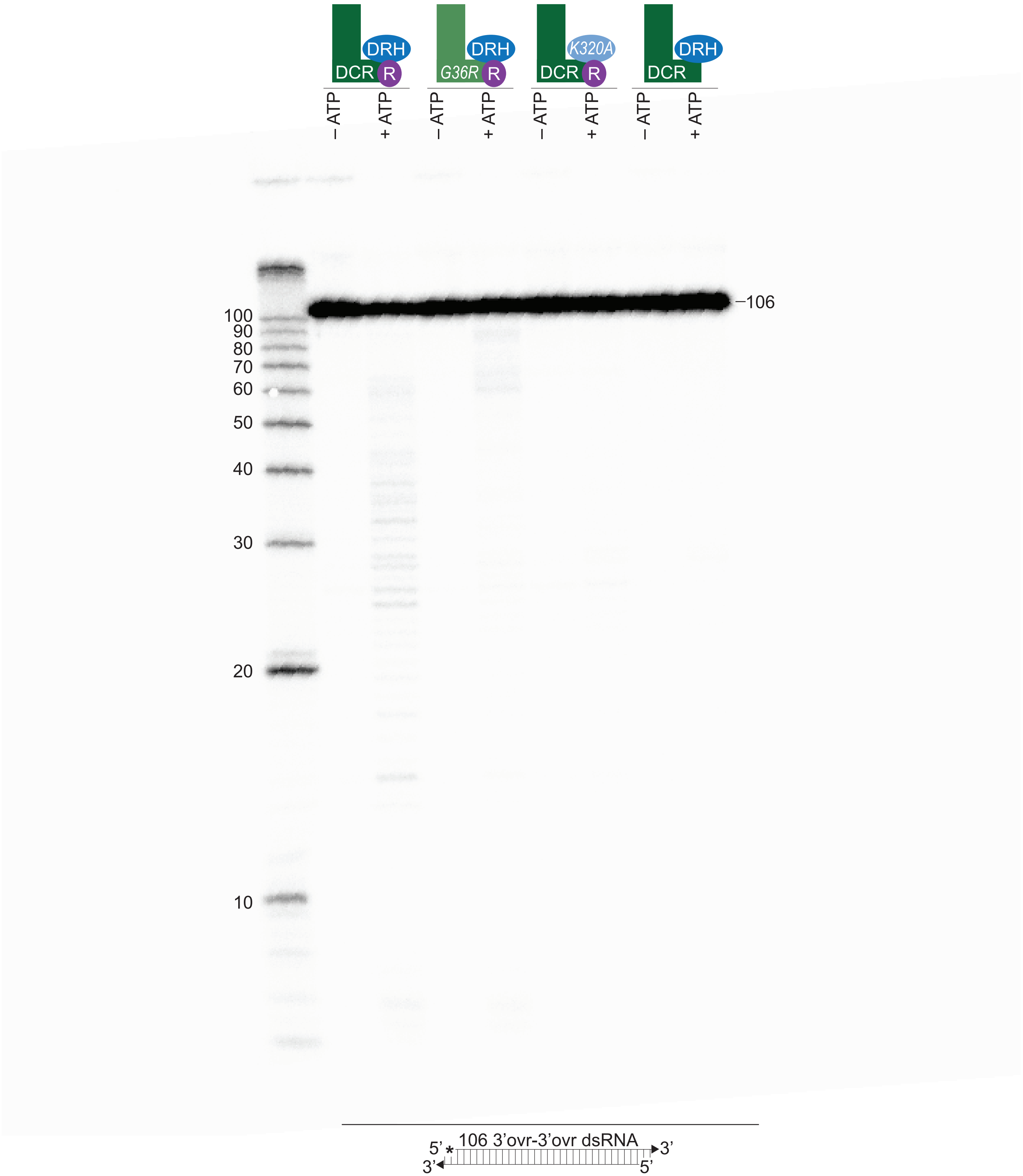

Figure 1 - figure supplement 2 - source data 2: Raw digital image of cleavage phosphorimager plate used in Figure 1 - figure supplement 2B.
