# Supplementary material for: Caenorhabditis elegans Dicer acts with the RIG-I-like helicase DRH-1 and RDE-4 to cleave dsRNA
Source: eLife. 2024 May 15;13:RP93979. doi: 10.7554/eLife.93979 (PMC11095941; doi:10.7554/eLife.93979)
Supplement: Figure 1—figure supplement 3—source data 1. [file elife-93979-fig1-figsupp3-data1.zip › Figure 1 - figure supplement 3 - source data 1.pdf]

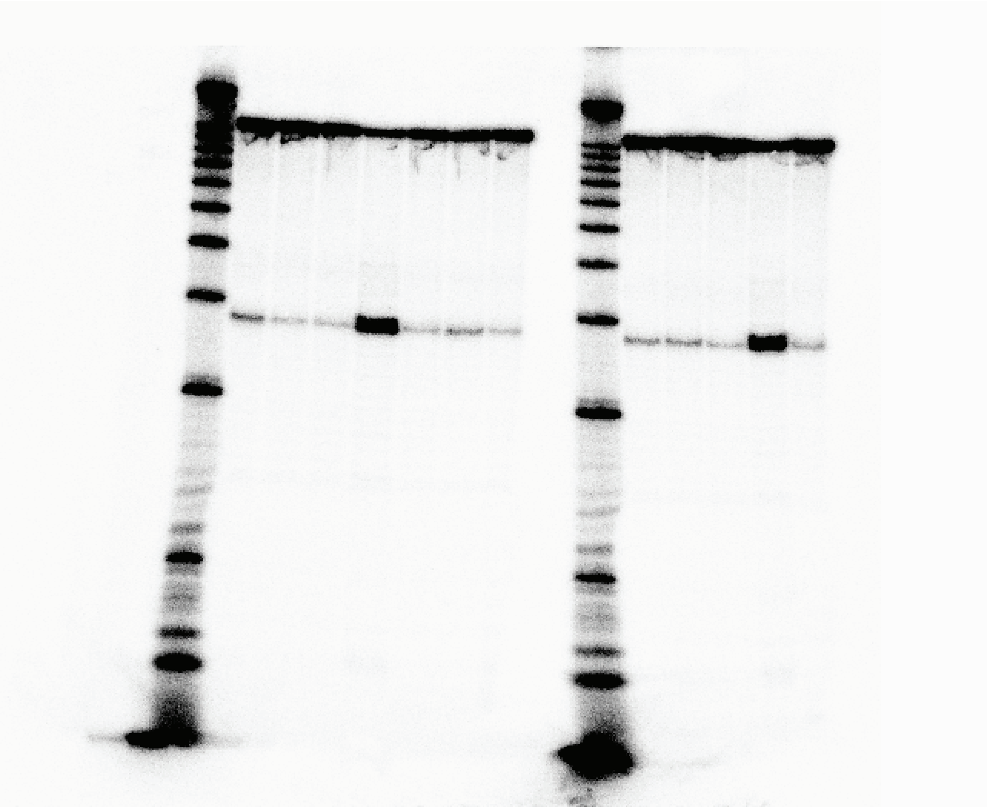

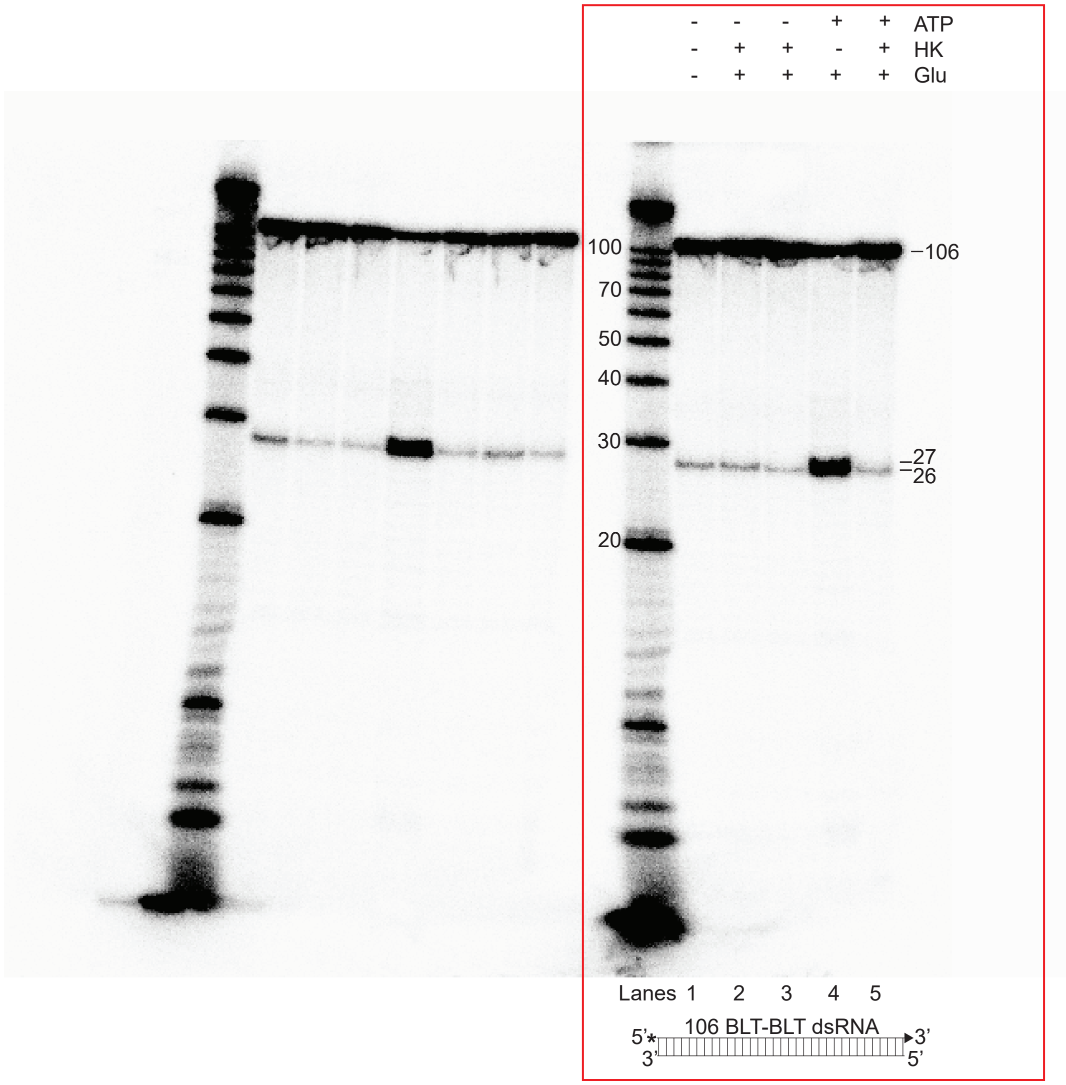

Figure 1 - figure supplement 3 - source data 1: Duplicate raw digital images of cleavage phosphorimager plates. Image on the bottom shows region of the plate used in Figure 1 - figure supplement 3A.
