# Supplementary material for: Caenorhabditis elegans Dicer acts with the RIG-I-like helicase DRH-1 and RDE-4 to cleave dsRNA
Source: eLife. 2024 May 15;13:RP93979. doi: 10.7554/eLife.93979 (PMC11095941; doi:10.7554/eLife.93979)
Supplement: Figure 2—source data 4. [file elife-93979-fig2-data4.zip › FIGURE 2 - SOURCE DATA 4.pdf]

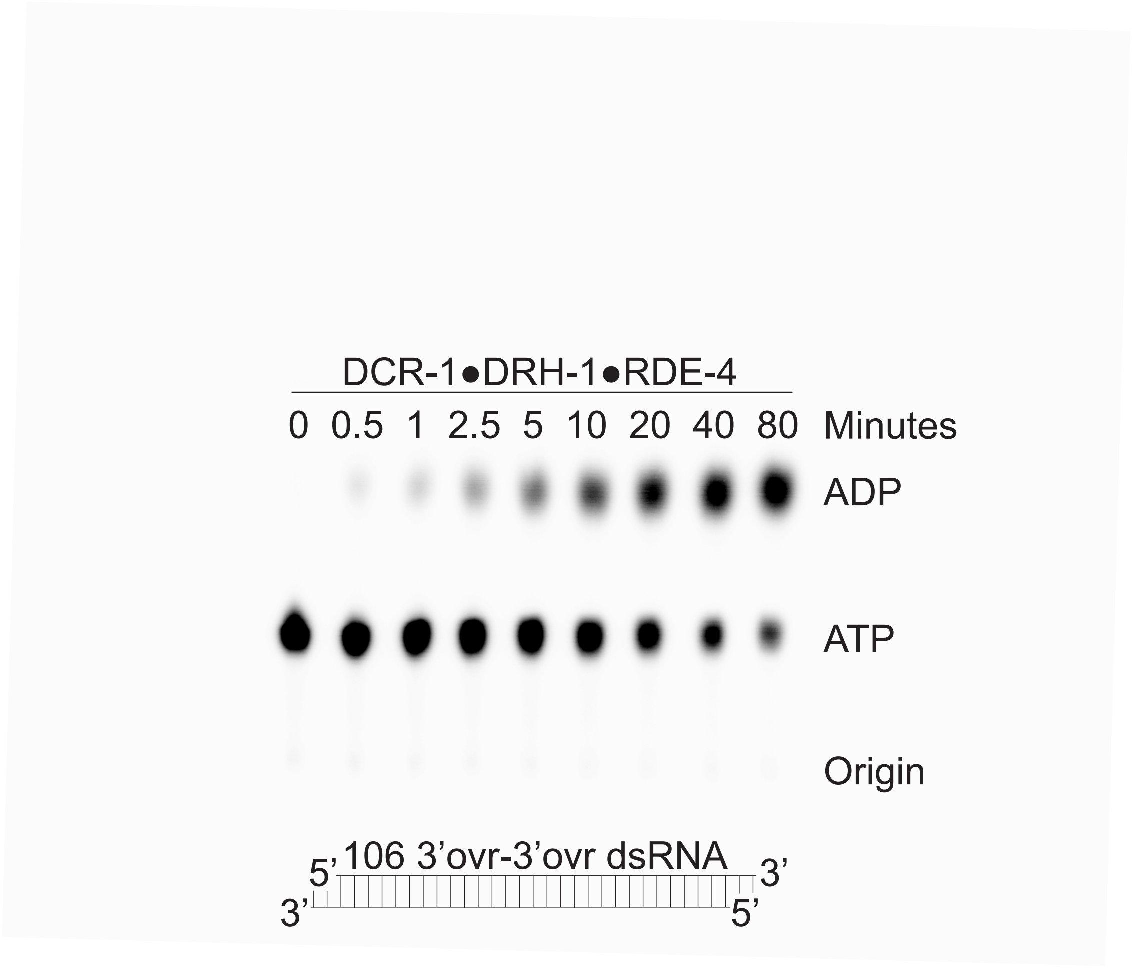

Figure 2 - source data 4: Raw digital image of thin-layer chromatography plate used in Figure 2D, right panel.
