# Supplementary material for: Caenorhabditis elegans Dicer acts with the RIG-I-like helicase DRH-1 and RDE-4 to cleave dsRNA
Source: eLife. 2024 May 15;13:RP93979. doi: 10.7554/eLife.93979 (PMC11095941; doi:10.7554/eLife.93979)
Supplement: Figure 2—figure supplement 1—source data 5. [file elife-93979-fig2-figsupp1-data5.zip › Figure 2 - figure supplement 1 - source data 5.pdf]

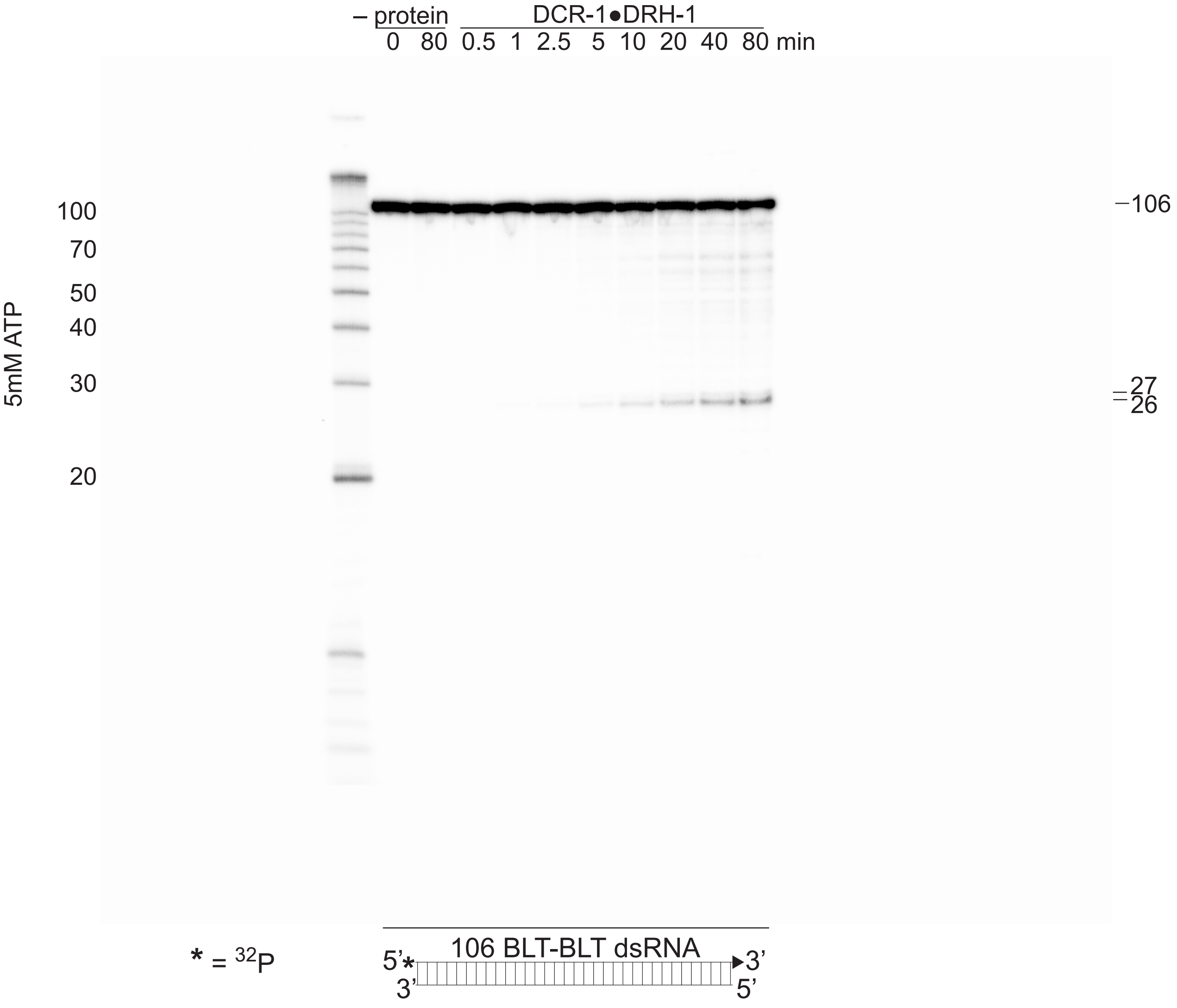

Figure 2 - figure supplement 1 - source data 5: Raw digital image of cleavage phosphorimager plate used in Figure 2 - figure supplement 1C, left panel.
