# Supplementary material for: Caenorhabditis elegans Dicer acts with the RIG-I-like helicase DRH-1 and RDE-4 to cleave dsRNA
Source: eLife. 2024 May 15;13:RP93979. doi: 10.7554/eLife.93979 (PMC11095941; doi:10.7554/eLife.93979)
Supplement: Figure 3—source data 3. [file elife-93979-fig3-data3.zip › FIGURE 3 - SOURCE DATA 3.pdf]

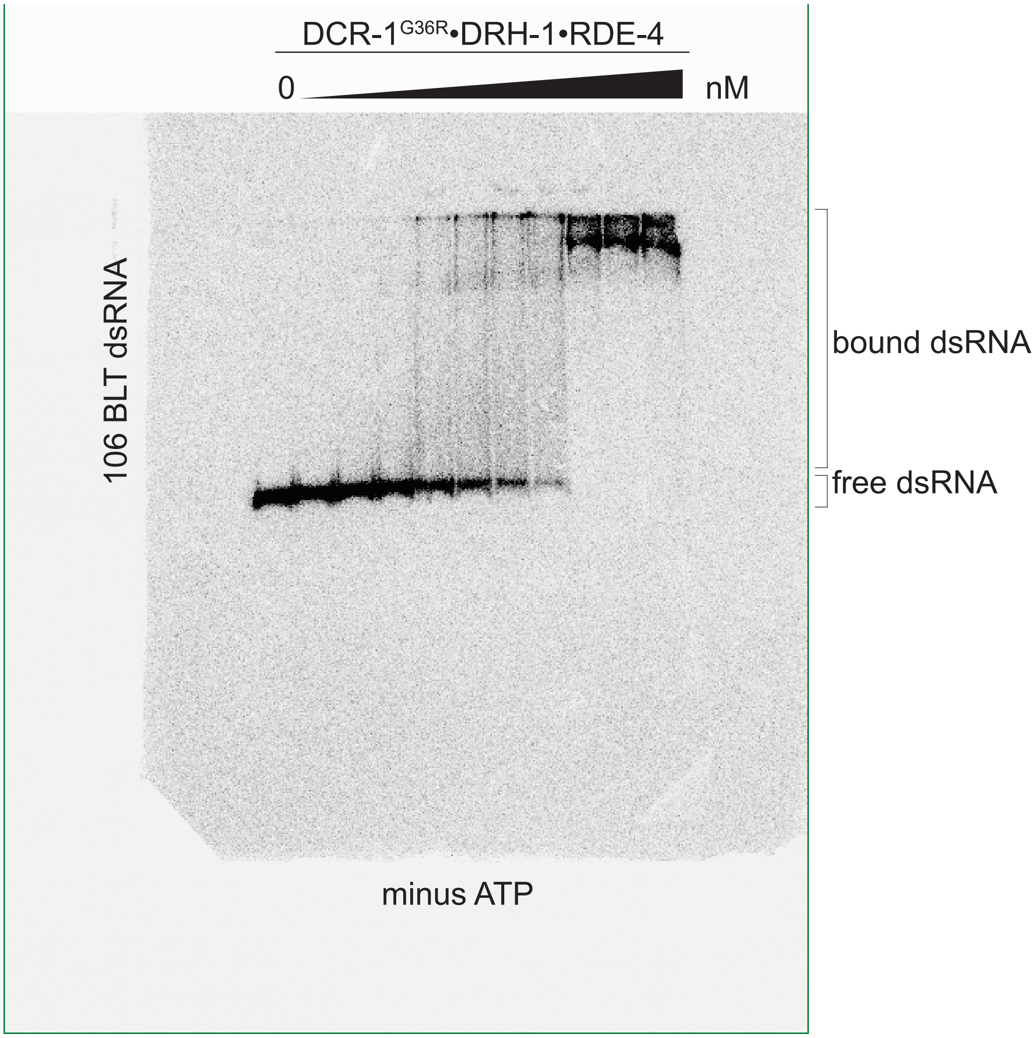

Figure 3 - source data 3: Raw digital image of gel shift phosphorimager plate used in Figure 3B, left panel.
