# Supplementary material for: Caenorhabditis elegans Dicer acts with the RIG-I-like helicase DRH-1 and RDE-4 to cleave dsRNA
Source: eLife. 2024 May 15;13:RP93979. doi: 10.7554/eLife.93979 (PMC11095941; doi:10.7554/eLife.93979)
Supplement: Supplementary file 1. — (a) Summary of Kd values. (b) Cryo-EM data collection, refinement, and validation statistics. [file elife-93979-supp1.docx]

| **Supplementary File 1a:** **Summary of K_d_ values** | | |
| --- | --- | --- |
| **Kd (nM)** | | |
|  | **DCR-1•DRH-1•RDE-4** | **DCR-1•DRH-1** |
| 106 BLT + 5mM ATPγS | 0.86 ± 0.06 | 19.63 ± 1.67 |
| 106 BLT 2’3’ cyclic phosphate on both ends minus ATP | 0.30 ± 0.04 | 5.65 ± 0.71 |
| 106 BLT 2’3’ cyclic phosphate on both ends + 5mM ATP | 0.67 ± 0.09 | 5.06 ± 0.40 |

Values shown are mean ± SD (n = 3).

| **Supplementary File 1b: Cryo-EM data collection, refinement, and validation statistics** | | | |
| --- | --- | --- | --- |
| **Data Collection and processing** | | | |
|  | DRH-1 | Complex bound to one dsRNA | Complex bound to two dsRNA |
| EM Databank Accession ID | EMD-41060 | EMD-43430 | EMD-43431 |
| Microscope | Titan Krios G3 | Titan Krios G3 | Titan Krios G3 |
| Voltage (kV) | 300 | 300 | 300 |
| Detector | Gatan K3 | Gatan K3 | Gatan K3 |
| ­Data collection software | SerialEM | Leginon | SerialEM |
| Nominal magnification | 81,000x | 81,000x | 81,000x |
| Total number of frames | 40 | 40 | 50 |
| Total electron exposure (e^-^/Å) | 38 | 50 | 50 |
| Defocus range (µm) | -1.0 - -2.0 µm | -1.0 - -2.0 µm | -1.0 - -2.0 µm |
| Pixel size (Å) | 0.529 Å | 0.533 Å | 0.394 Å |
| Number of micrographs | 14,602 | 24,705 | 3,866 |
| Final particles | 362,869 | 171,304 | 26,879 |
| Resolution (GSFSC 0.143) | 2.9 Å | 6.1 Å | 7.6 Å |
| **Refinement and validation statistics for 2.9** **Å reconstruction of DRH-1** | | | |
| **Structure** | | | |
| Protein Data Bank Accession ID | 8T5S | | |
| **Symmetry imposed** | | | |
| 3D classification | C1 | | |
| 3D refinement | C1 | | |
| Final particles | 362,869 | | |
| **Map resolution (Å)** | | | |
| FSC 0.143 (unmasked) | 3.6 Å | | |
| FSC 0.143 (masked, corrected) | 2.9 Å | | |
| **Model Refinement** | | | |
| Initial model used (AlphaFold database) | AF-G5EDI8-F1 | | |
| Map correlation coefficient | 0.8 | | |
| **Model composition** | | | |
| Non-hydrogen atoms | 6779 | | |
| Protein residues | 693 | | |
| Nucleotides | 60 | | |
| Ligands (ADP, Mg^2+^, Zn^2+^) | 3 | | |
| **R.m.s. deviations** | | | |
| Bond lengths (Å) | 0.002 | | |
| Bond angles (°) | 0.499 | | |
| **Validation** | | | |
| MolProbity score | 1.72 | | |
| Clashscore | 10.02 | | |
| Poor rotamers (%) | 0.48 | | |
| **Ramachandran plot** | | | |
| Favored (%) | 96.79 | | |
| Allowed (%) | 3.21 | | |
| Disallowed (%) | 0.00 | | |
| C-beta deviations (0.25 Å) | 0.00 | | |
| CaBLAM outliers (%) | 2.81 | | |
