# Supplementary material for: Caenorhabditis elegans Dicer acts with the RIG-I-like helicase DRH-1 and RDE-4 to cleave dsRNA
Source: eLife. 2024 May 15;13:RP93979. doi: 10.7554/eLife.93979 (PMC11095941; doi:10.7554/eLife.93979)
Supplement: Figure 2—figure supplement 1—source data 7. [file Figure 2 - figure supplement 1 - source data 7.pdf › Figure 2 - figure supplement 1 - source data 7.pdf]

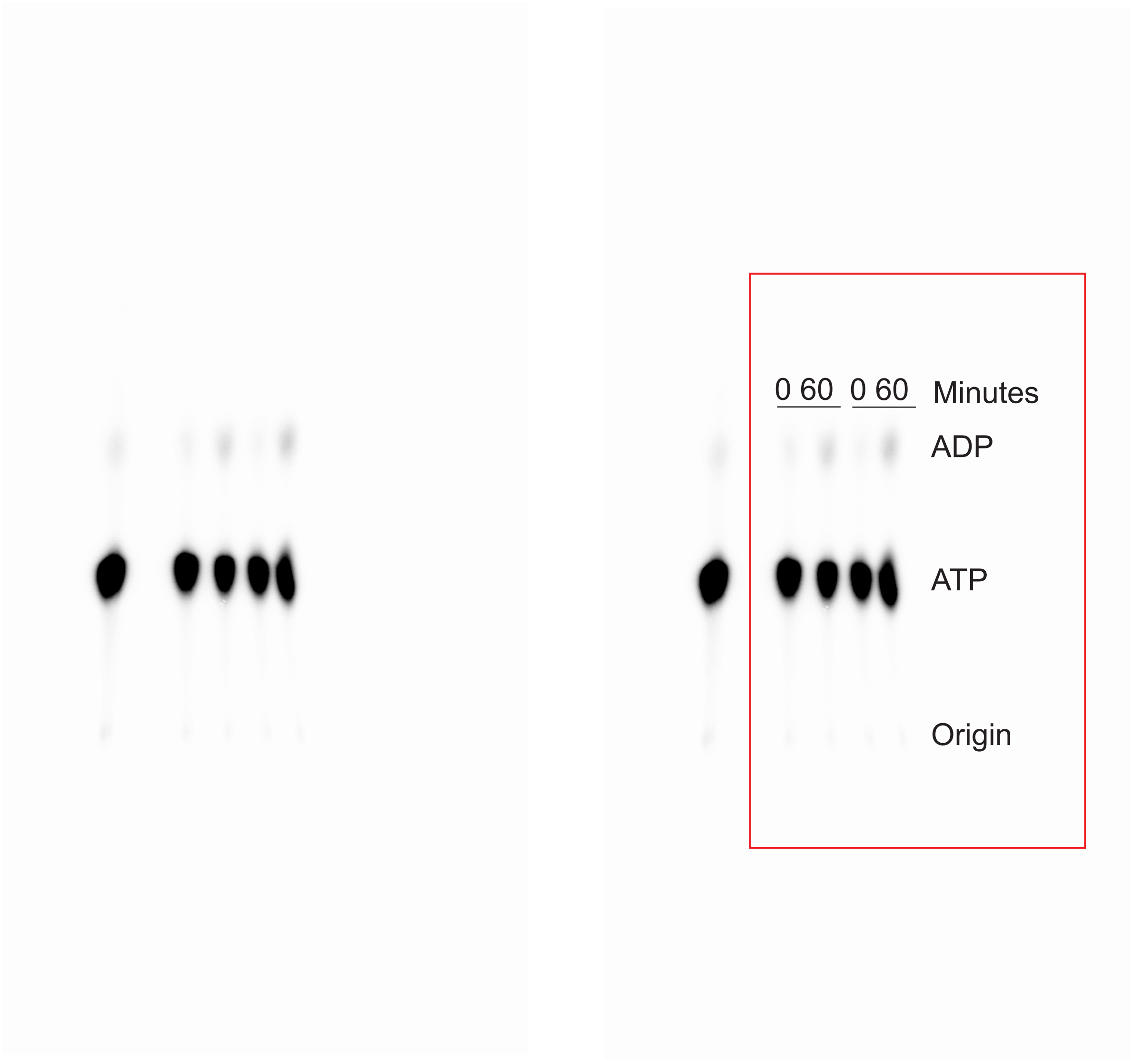

Figure 2 - figure supplement 1 - source data 7: Duplicate raw digital images of thin-layer chromatography plates. Image on the right shows region of the plate used in Figure 2 - figure supplement 1D.
